# The impact of integrating environmental health into medical school curricula: a survey-based study

**DOI:** 10.1186/s12909-020-02458-x

**Published:** 2021-01-08

**Authors:** Benjamin Kligler, Genevieve Pinto Zipp, Carmela Rocchetti, Michelle Secic, Erin Speiser Ihde

**Affiliations:** 1 The Deirdre Imus Environmental Health Center®, Hackensack University Medical Center, Hackensack, NJ USA; 2 Department of Interprofessional Health Sciences & Health Administration, School of Health and Medical Sciences, Seton Hall University, Nutley, NJ USA; 3 Hackensack Meridian School of Medicine, Nutley, NJ USA; 4 Secic Statistical Consulting, Inc., Chardon, OH USA

**Keywords:** Environmental health, Medical education, Prevention, Environmental justice, Health disparities, Climate change

## Abstract

**Background:**

Inclusion of environmental health (EH) in medical education serves as a catalyst for preparing future physicians to address issues as complex as climate change and health, water pollution and lead contamination. However, previous research has found EH education to be largely lacking in U.S. medical education, putting future physicians at risk of not having the expertise to address patients’ environmental illnesses, nor speak to prevention.

**Methods:**

Environmental health (EH) knowledge and skills were incorporated into the first-year medical school curriculum at Hackensack Meridian School of Medicine (Nutley, New Jersey), via a two-hour interactive large group learning module with follow up activities. Students completed the Environmental Health in Med School (EHMS) survey before and after the year 1 EH module. This survey evaluates medical students’ attitudes, awareness and professionalism regarding environmental health. In year 2, students completed the Environmental Health Survey II, which measured students’ perceptions of preparedness to discuss EH with future patients. The research team created both surveys based upon learning objectives that broadly aligned with the Institute of Medicine six competency-based environmental health learning objectives.

**Results:**

36 year 1 students completed both the pre and post EHMS surveys. McNemar’s test was used for paired comparisons. Results identified no statistically significant changes from pre to post surveys, identifying a dramatic ceiling.

When comparing year 2, EHS II pre-survey (*n* = 84) and post-survey (*n* = 79) responses, a statistically significant positive change in students’ self-reported sense of preparedness to discuss environmental health with their patients following the curriculum intervention was noted.

**Conclusions:**

Our conclusion for the EHMS in Year 1 was that the current generation of medical students at this school is already extremely aware of and concerned about the impact of environmental issues on health.

Through the EHS II in Year 2, we found that the six-week environmental health module combining didactic and experiential elements significantly increased medical students’ self-reported sense of preparedness to discuss environmental health issues, including climate change, with their patients.

## Background

The World Health Organization estimates 23% of global disease is attributable to environmental factors, which rises to 25% for children under age five [1]. Inclusion of environmental health (EH) in medical education serves as a catalyst for preparing future physicians to address issues as complex as climate change and health, water pollution and lead contamination in our communities [2–4]. However, previous research has found EH education to be largely lacking in U.S. medical education, putting future physicians at risk of not having the expertise necessary to address patients’ environmental illnesses, nor speak to prevention [4–6]. This is of particular concern for future pediatricians, as children are especially vulnerable given their immature detoxification pathways and tendencies to play close to the ground where contaminants settle [7, 8]. Other vulnerable populations include the elderly, those with pre-existing health conditions and marginalized populations. Environmental health disparities, therefore, fit well under the larger umbrella of social determinants of health, which are garnering increasing attention yet still remain largely omitted from medical school curricula [9–12].

As part of its commitment to addressing social determinants of health in the medical school curriculum, Hackensack Meridian School of Medicine (HMSOM) in Nutley, New Jersey developed, launched and tested a new environmental health module in the required first year curriculum. The goal was to produce an effective and reproducible curriculum which could be utilized by other medical schools to address the gap in environmental health knowledge among physicians.

## Methods

The new curriculum module was incorporated into the Human Dimension (HD), a three-year longitudinal course at the heart of the HMSOM curriculum. Through service-learning experiences and an integrated curriculum, students understand the many Determinants of Health, including the social determinants of health as well as the personal, economic, and environmental determinants. Students are matched to individuals and families from underserved areas, and through longitudinal interactions over the entire core curriculum, they become involved in all aspects of the family’s life to understand drivers of health outcomes, provide education, and navigate community resources. Activities include meetings and calls with individuals and families in their homes and communities as well as participation in multidisciplinary teams in health care, legal, and social services settings.

As part of this effort, the research team created an Environmental Health module delivered via a two-hour interactive large group learning module (lecture) with follow-up activities (a six-week-long student activity highlighting the ways in which the environment and medicine intersect and then a small group discussion session to process that experience). The lecture was titled, “Environmental Health: What Do Physicians Need to Know?” and given by BK. Lecture topics included the health effects of exposures to common toxins, avoiding carcinogens and endocrine disruptors, choosing healthier food and personal care products, the health impacts of climate change, and identifying populations at most risk for environmental health issues. The lecture included two small group discussion periods on questions of how to communicate effectively with patients regarding environmental health and how to begin to make changes in one’s own behaviors. The activity had two components; first, students were asked to download the “Healthy Living” app produced by Environmental Working Group, which provides details on the environmental safety of a wide range of foods and personal care products. Students were asked to explore their diet and personal care choices by identifying 5–10 items and using the app to assess the relative safety of those products from an environmental health perspective. In addition, students were provided with a set of assessment tools to use to understand the potential environmental exposure risk of their HD family. The EH module is delivered midway through the first year of the HD curriculum, and both small group discussions and the community-based work with the HD family is overseen by the HD course faculty who have received faculty development sessions on this topic.

The EH module was given during two subsequent years at the medical school. In year 1, the 15-question Environmental Health in Med School (EHMS) survey was given in January 2019 and the post was given in March 2019. This tool was developed by ESI, GPZ and BK. While there were approximately 60 first-year students invited to take the survey, there were 36 subjects who had data on both the pre and post surveys and were included in the statistical analysis.

For statistical analysis of the EHMS, data were summarized for each question both pre and post with counts and percentages. Responses on the pre survey were compared to those on the post survey using McNemar’s test of agreement. For statistical purposes, results were collapsed as follows, excluding neutrals: True = Definitely true/Somewhat true; False = Definitely false/Somewhat false; Agree = Strongly agree/Agree; Disagree = Strongly disagree/Disagree.

In year 2, students took a newly-created 4-question Environmental Health Survey II (EHS II) before their EH module in November 2019 and again as a post survey in March 2020. This tool was created by BK, who gave the EH lecture in the large group learning module. A total of 84 students took the pre survey, and 79 took the post.

The two surveys were designed to help inform subsequent revisions to the module content to ensure learning objectives were met. These objectives broadly align with the six competency-based environmental health learning objectives from the Institute of Medicine [13].

The Year 1 EHMS survey was designed to evaluate medical students’ attitudes, awareness and professionalism regarding environmental health. Each of the three constructs that were measured had five corresponding questions in the survey, for a total of 15 questions. A Delphi panel of five experts with backgrounds in curriculum design were asked to rate each question based on three criteria: Is the question appropriate for this survey, is the question clearly written, and is the question in the correct sequence? Each of these had yes/no response options followed by “If no, what are your suggestions for improvement?”

A total of two rounds of the Delphi process were conducted until at least 80% consensus was reached on each question. To control the validity issues associated with the Delphi technique, the survey questions were revised to provide clarity where needed. Based on this first survey, a new survey (EHS II) was created for Year 2 of new, incoming medical students. This brief survey was designed to measure students’ perceptions of preparedness to discuss EH with future patients as opposed to their attitudes, awareness, and professionalism. There were no tests of reliability or validity for this EHSII. The questions were: 1) I feel prepared to advise my patients on strategies for minimizing exposure to pesticides and other environmental toxins in their diet. 2) I feel prepared to advise my patients on strategies for minimizing exposure to environmental toxins in their household and personal care products. 3) I know enough to advocate in my community to try to reduce the health-related impact of environmental toxins on air and water quality. 4) I feel prepared to discuss the specific impacts of climate change on human health. Response options were organized into a 5-point Likert scale ranging from “totally disagree” to “totally agree.” For the EHSII, the Wilcoxon signed-rank test was used to ascertain whether the EH module was effective in promoting medical students’ preparedness to discuss environmental health with their patients.

Data from both surveys was collected non-anonymously using a secure system. To protect participants’ confidentiality, only the HMSOM Institutional Effectiveness and Assessment team had access to identifying information. They provided de-identified data to the research team, including unique identifiers created solely for this project to link the pre and post survey responses. The pre and post surveys fell under the written consent already obtained from the students for LongMED, a database that supports an epidemiologic, longitudinal, outcomes-focused approach to the study of medical education. LongMED contains protected, linked data tied to medical students at the HMSOM.

All students completed the pre and post surveys as part of the curriculum, but the HMSOM sent the research team only the responses for the students who consented to participate in LongMED. Students’ interaction with the instructor and their grade in the HD course were not affected in any way by taking or declining to take the pre and post survey. All first-year medical students (approximately 60 in Year 1 and 85 in Year 2) at the HMSOM, who consented to use LongMED and attended the HD Module, were invited to participate. The final study protocol, survey and data collection tool were approved by the Institutional Review Board (IRB) at Hackensack University Medical Center, Hackensack, New Jersey, USA.

The specific content of the lecture including PowerPoint slides as well as detailed descriptions of the other elements in the EH module are available as Additional files 1 and 2 to this paper.

## Results

For the EHMS in Year 1, 36 subjects had data on both the Pre and Post surveys and thus were eligible for the paired comparisons. In the 2 × 2 cross-tabulations, most subjects did not change their answers at all or significantly from pre to post. For example, for Q1, 4 subjects answered true on the Pre survey and all 4 of those answered false on the Post survey; 25 subjects answered false on the Pre survey and 23 of those answered false on the Post survey, while 2 of them answered true on the Post survey. Although 4 switched from true to false and 2 switched from false to true, the differences in these changes were not statistically significant, *p* = 0.41.

Therefore, no statistically significant changes from Pre to Post were found, using the standard two-tailed alpha-level of 0.05. Results of the pre and post for the first survey suggested a dramatic ceiling effect on students’ responses to the EHMS survey, making it difficult to detect any change from before to after the intervention.

**Fig. 1:**
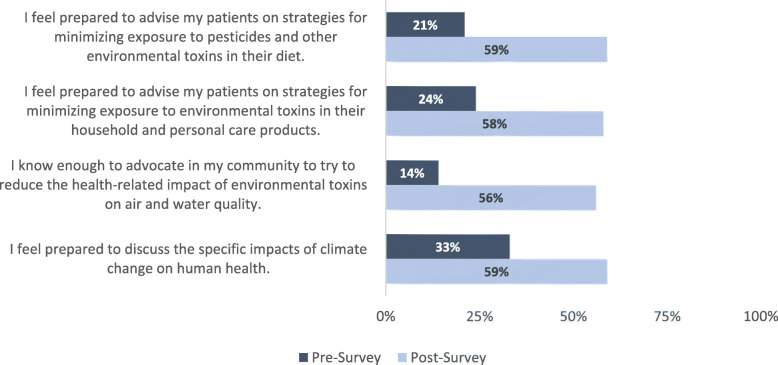
Percentage of respondents in each administration that responded ‘Totally Agree’ or ‘Somewhat Agree’ to the four EHS II questions, *n* = 84 on pre-survey (dark blue) and *n* = 79 on post-survey (light blue)

For the Year 2 survey (EHS II), Fig. 1 demonstrates the percentage of respondents across the 5-point Likert scale ranging from “totally disagree” to “totally agree” for the four questions on the pre-survey (84 respondents) and on the post-survey (79 respondents). As reported in Table 1, the Wilcoxon test showed a statistically significant positive change for each question posed in students’ self-reported sense of preparedness to discuss environmental health with their patients following the curriculum intervention.

**Table 1:** EHS II Means (SD) Analysis, Pre-Survey (*n* = 84) v. Post-Survey (*n* = 79)

Responses	Pre-Survey	Post-Survey	Wilcoxon Signed-Rank Test ***P***-Value
I feel prepared to advise my patients on strategies for minimizing exposure to pesticides and other environmental toxins in their diet.	2.31 (1.086)	3.46 (0.958)	<0.001
I feel prepared to advise my patients on strategies for minimizing exposure to environmental toxins in their household and personal care products.	2.48 (1.081)	3.47 (0.918)	<0.001
I know enough to advocate in my community to try to reduce the health-related impact of environmental toxins on air and water quality.	2.27 (0.986)	3.39 (1.005)	<0.001
I feel prepared to discuss the specific impacts of climate change on human health.	2.80 (1.210)	3.46 (1.095)	<0.001

*SD* Standard Deviation

Totally Agree = 5, Somewhat Agree = 4, Neither Agree nor Disagree = 3, Somewhat Disagree = 2, Totally Disagree = 1

## Discussion

As early as 1995, the Institute of Medicine (IOM) recommended six competency-based EH learning objectives for medical students, as shown in Table 2:

**Table 2:** Institute of Medicine Competency Based Environmental Health Learning Objectives for Medical Students

IOM Competency Based EH Learning Objectives	
1. understand the influence of the environment and environmental agents on human health based on knowledge of relevant epidemiologic, toxicologic, and exposure factors	
2. recognize the signs, symptoms, diseases, and sources of exposure relating to common environmental agents and conditions	
3. elicit an appropriately detailed environmental exposure history, including a work history, from all patients	
4. identify and access the informational, clinical, and other resources available to help address patient and community environmental health problems and concerns	
5. discuss environmental risks with their patients and provide understandable information about risk-reduction strategies in ways that exhibit sensitivity to patients’ health beliefs and concerns	
6. understand the ethical and legal responsibilities of seeing patients with environmental and occupational health problems or concerns [13] (p.3)	

Despite these recommendations, though, and the growing awareness of the health impacts of climate change and environmental degradation, there is no standard requirement to include EH coursework in medical schools. To our knowledge this is the first evaluation and description of an environmental health curriculum intervention which demonstrates significant change in students’ sense of preparedness to discuss EH with their patients.

Although several medical schools do offer some exposure to EH, including Harvard and Mount Sinai, there is a general sense that finding time in the curriculum for this subject matter is still difficult [14]. Climate change in particular is apparently being overlooked: a survey of the Curriculum Inventory of the American Association of Medical Colleges in 2017 revealed no explicit mention of climate change in any medical school curriculum [15] though efforts to increase climate change in medical curricula now exist [14]. The lack of such curriculum continues to lead to a sense of unpreparedness in providers to discuss this area with patients: a 2010 survey of pediatric providers reported a lack of training in environmental history taking and low self-efficacy in how to manage patients’ exposures [7]. A similar survey of pediatric oncologists found that over 90% of respondents felt that “more knowledge of the associations between environmental exposures and childhood cancer would be helpful in addressing these issues with patients” [16].

Integrating EH material into existing courses - as we have done at the HMSOM - may be more feasible than restructuring an already dense curriculum to include dedicated EH courses [5, 17]. Distance-based education and engagement of basic science faculty to teach some competencies may help alleviate the shortage of faculty trained in environmental medicine [5]. A new multimedia e-book developed by Miller et al. and endorsed by the Centers for Disease Control and Prevention (CDC) - “A Story of Health” - is an excellent resource as well, especially where local faculty expertise in EH may be lacking [18]. Finally, it is also important to combine didactic with experiential and small-group learning as we have done, particularly when the goal is to impact students’ sense of preparedness and self-efficacy around discussing EH with patients. Experience discussing this issue with colleagues and experimenting with actually making behavioral changes in their own lives are also critical.

## Limitations

A limitation in this study is the use of self-assessed outcomes rather than objective observation. This could be addressed by incorporating EH concepts into a standardized patient assessment experience where those resources are available. A second limitation of this study is that we present results of the preparedness survey from only one cadre of medical students. Further research on the impact of incorporating environmental health into required curriculum is needed, especially on the reproducibility of this particular module in increasing competency for medical student learners in this important area both at HMSOM and at other medical schools that may utilize this curriculum.

## Conclusions

The EHMS in Year 1 suggested the current generation of medical students at this school is already extremely aware of and concerned about the impact of environmental issues on health. Analysis of the EHS II in Year 2 showed that a relatively brief six-week environmental health module combining didactic and experiential elements can significantly increase medical students’ self-reported sense of preparedness to discuss environmental health issues, including climate change, with their patients.

## Supplementary Information

**Additional file 1.**

**Additional file 2.**

## Data Availability

The de-identified data sets generated and analyzed during the current study will be made available from the corresponding author, on reasonable request, to any scientist wishing to use them for non-commercial purposes. The authors are also happy to share the detailed content of the presentations and small group activities included in this module.

## Abbreviations

CDC: Centers for Disease Control and Prevention
EH: Environmental health
EHMS: Environmental Health in Medical School
EHS II: Environmental Health Survey II
HMSOM: Hackensack Meridian School of Medicine
HD: Human Dimension
