# Supplementary material for: The impact of integrating environmental health into medical school curricula: a survey-based study
Source: BMC Med Educ. 2021 Jan 8;21:40. doi: 10.1186/s12909-020-02458-x (PMC7796639; doi:10.1186/s12909-020-02458-x)
Supplement: Supplementary file 1 — Additional file 1. [file 12909_2020_2458_MOESM1_ESM.pptx]

## Slide 1
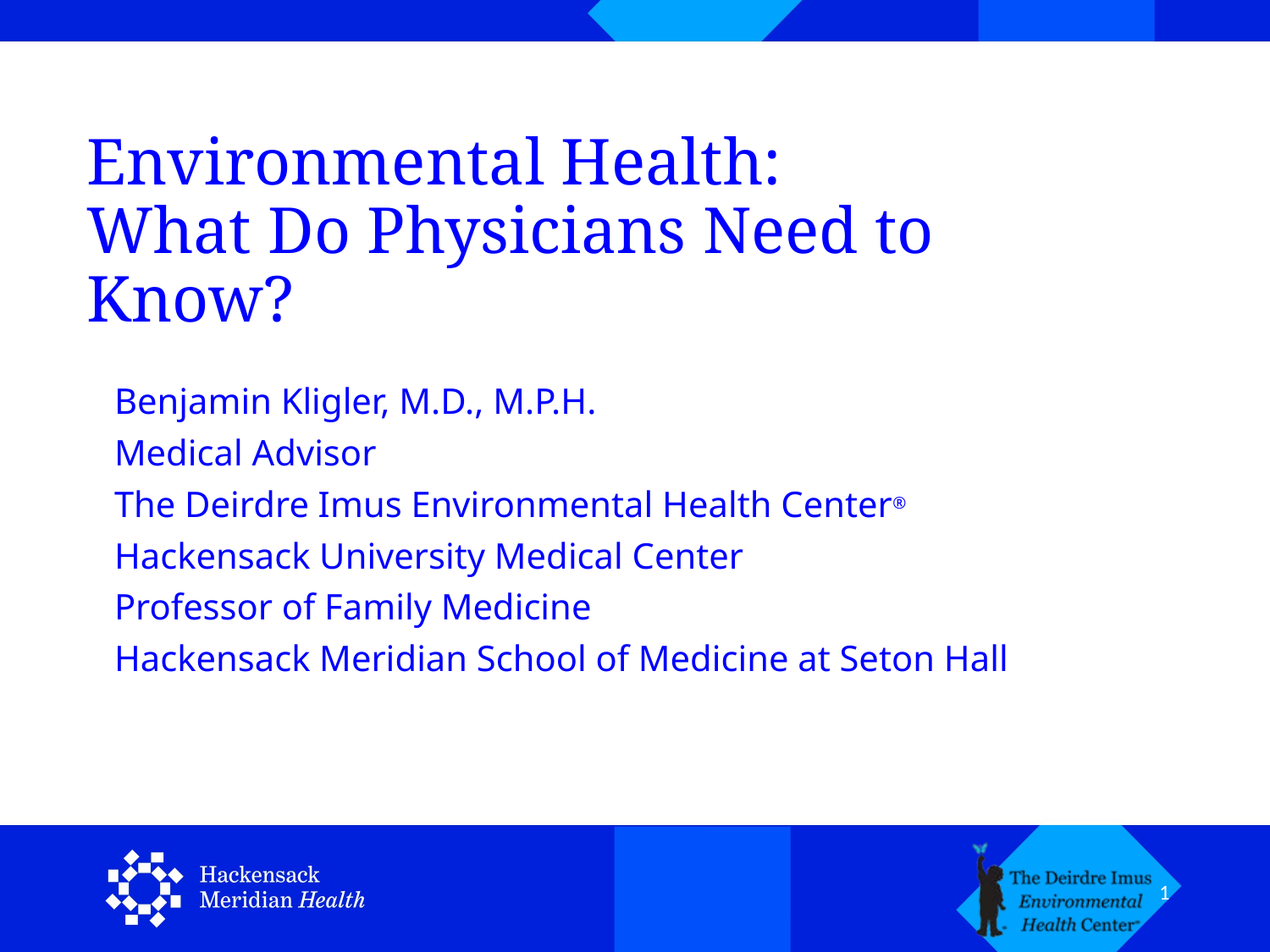

Environmental Health:
What Do Physicians Need to Know?
Benjamin Kligler, M.D., M.P.H.
Medical Advisor
The Deirdre Imus Environmental Health Center®
Hackensack University Medical Center
Professor of Family Medicine
Hackensack Meridian School of Medicine at Seton Hall
1

## Slide 2
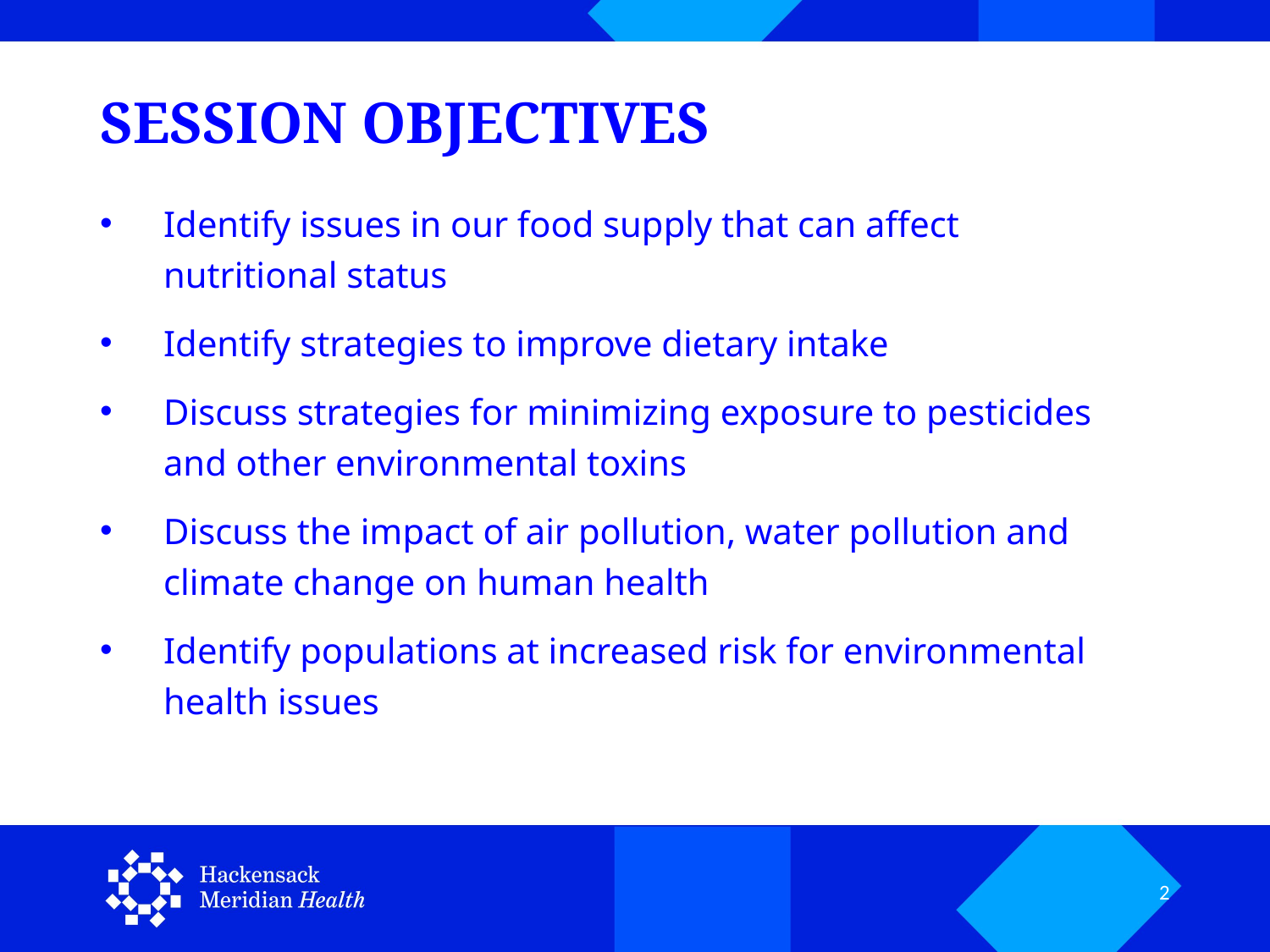

SESSION OBJECTIVES
Identify issues in our food supply that can affect nutritional status
Identify strategies to improve dietary intake
Discuss strategies for minimizing exposure to pesticides and other environmental toxins
Discuss the impact of air pollution, water pollution and climate change on human health
Identify populations at increased risk for environmental health issues
2

## Slide 3
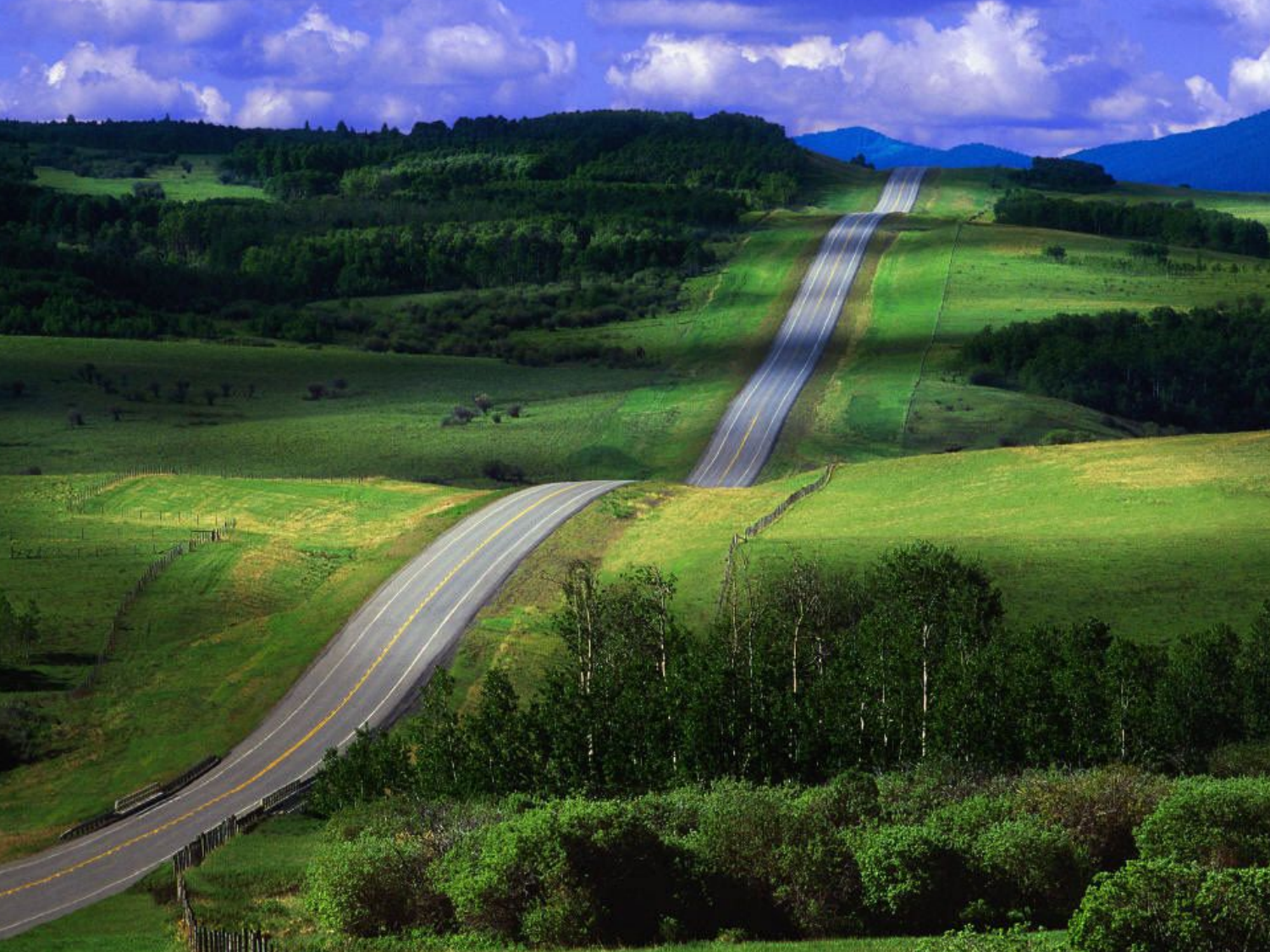

3

## Slide 4
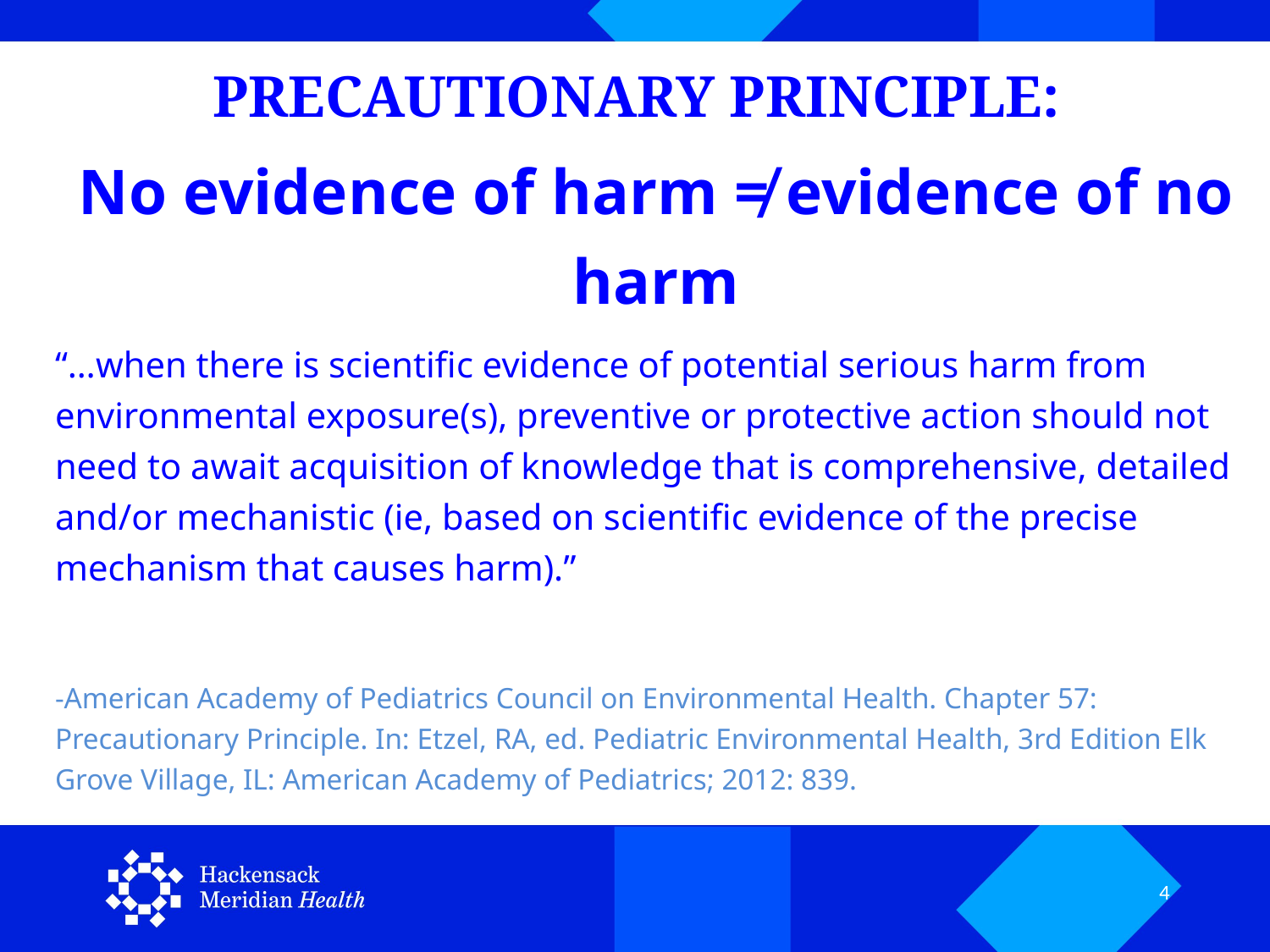

PRECAUTIONARY PRINCIPLE:
No evidence of harm ≠ evidence of no harm
“…when there is scientific evidence of potential serious harm from environmental exposure(s), preventive or protective action should not need to await acquisition of knowledge that is comprehensive, detailed and/or mechanistic (ie, based on scientific evidence of the precise mechanism that causes harm).”
-American Academy of Pediatrics Council on Environmental Health. Chapter 57: Precautionary Principle. In: Etzel, RA, ed. Pediatric Environmental Health, 3rd Edition Elk Grove Village, IL: American Academy of Pediatrics; 2012: 839.
4

## Slide 5
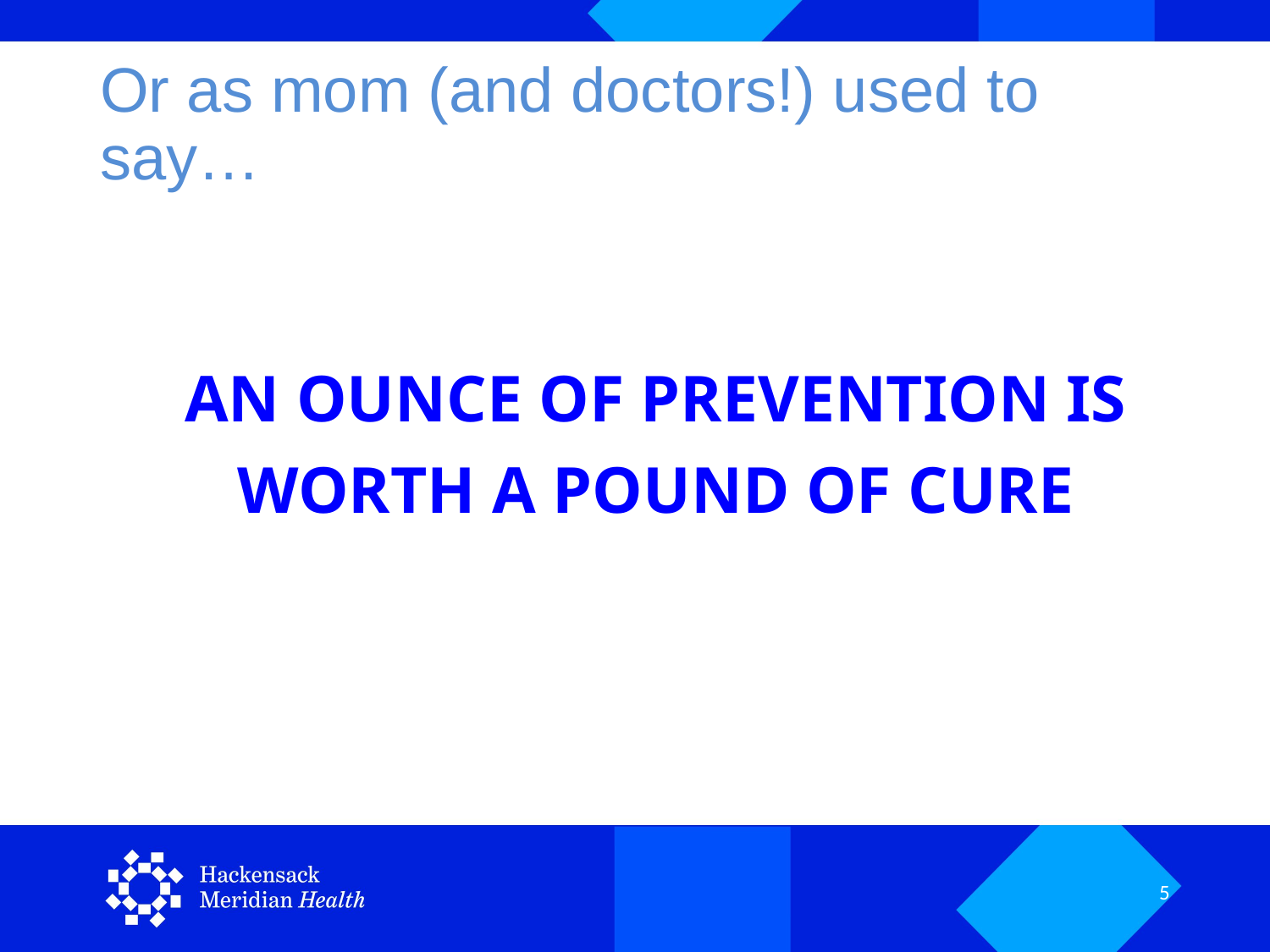

Or as mom (and doctors!) used to say…
AN OUNCE OF PREVENTION IS WORTH A POUND OF CURE
5

## Slide 6
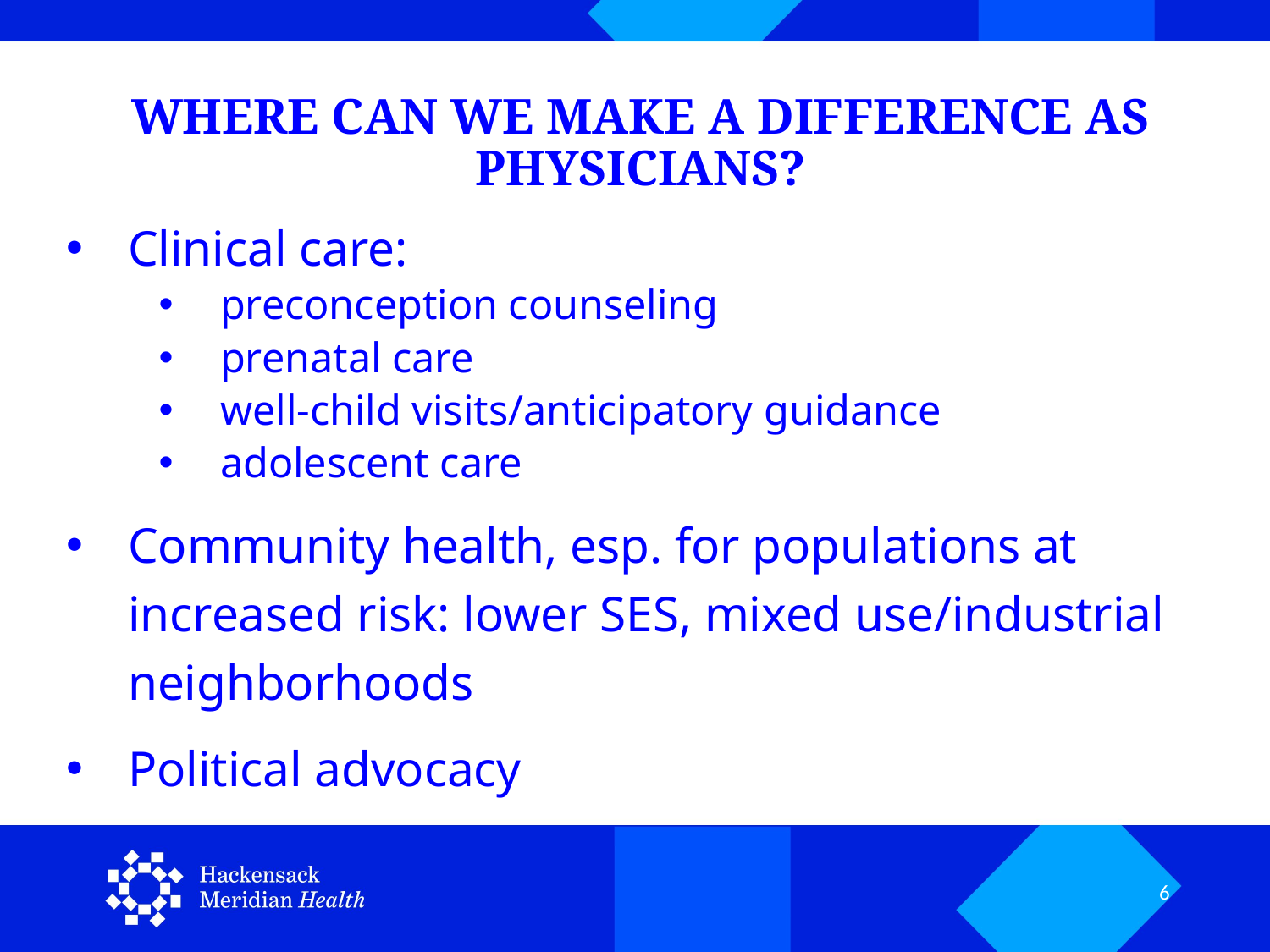

# WHERE CAN WE MAKE A DIFFERENCE AS PHYSICIANS?
Clinical care:
preconception counseling
prenatal care
well-child visits/anticipatory guidance
adolescent care
Community health, esp. for populations at increased risk: lower SES, mixed use/industrial neighborhoods
Political advocacy
6

## Slide 7
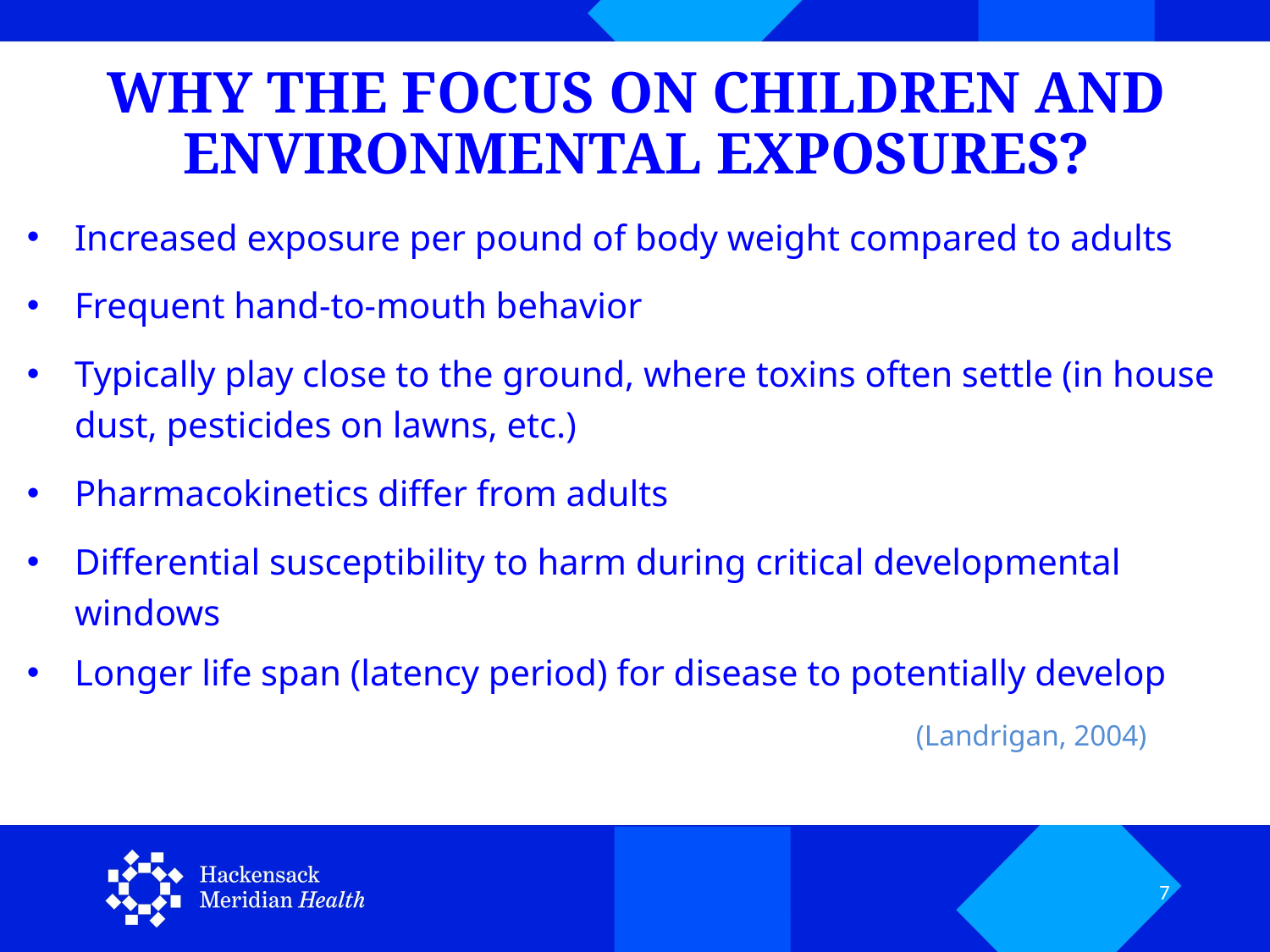

WHY THE FOCUS ON CHILDREN AND ENVIRONMENTAL EXPOSURES?
Increased exposure per pound of body weight compared to adults
Frequent hand-to-mouth behavior
Typically play close to the ground, where toxins often settle (in house dust, pesticides on lawns, etc.)
Pharmacokinetics differ from adults
Differential susceptibility to harm during critical developmental windows
Longer life span (latency period) for disease to potentially develop
							(Landrigan, 2004)
7

## Slide 8
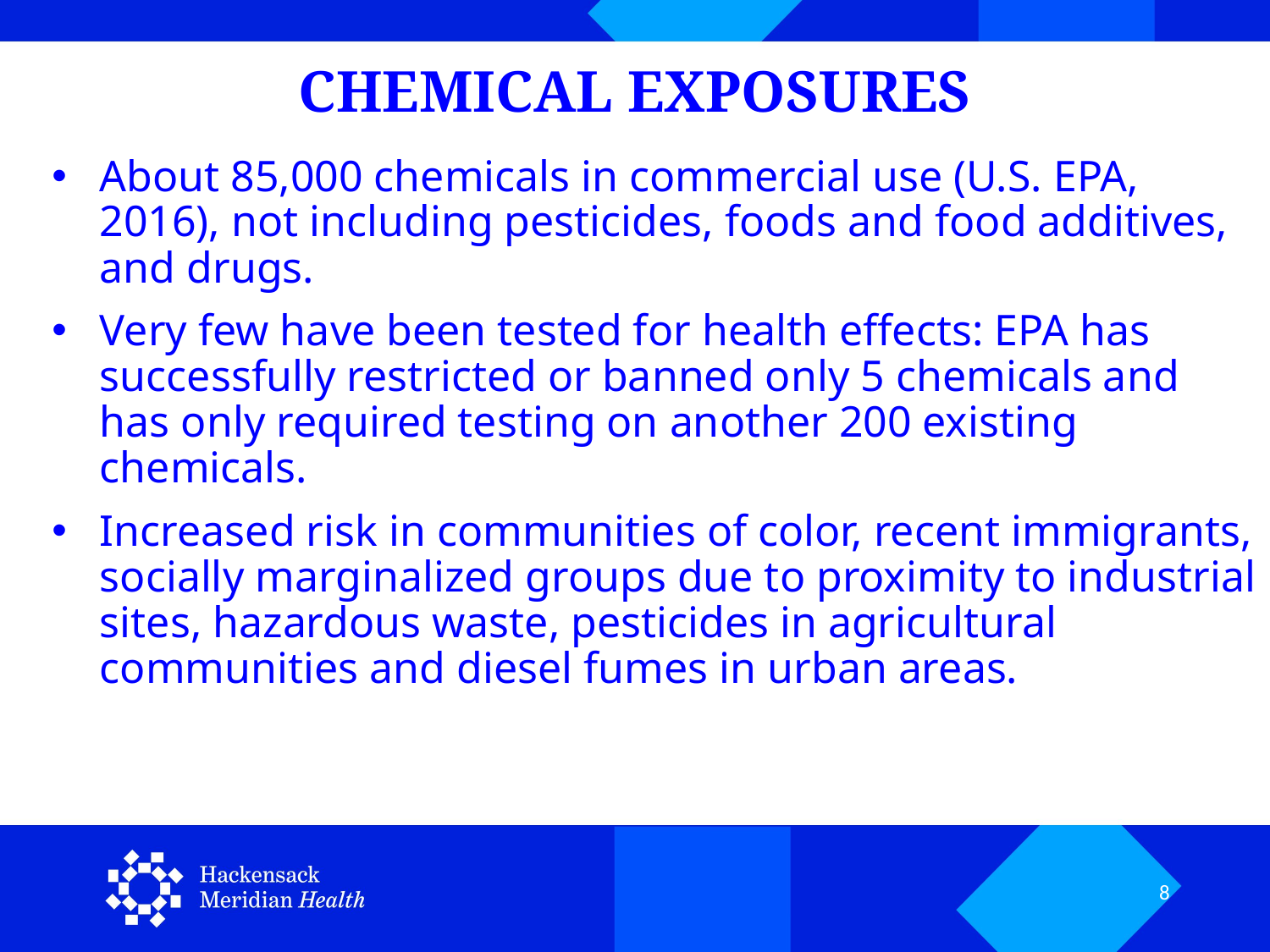

CHEMICAL EXPOSURES
About 85,000 chemicals in commercial use (U.S. EPA, 2016), not including pesticides, foods and food additives, and drugs.
Very few have been tested for health effects: EPA has successfully restricted or banned only 5 chemicals and has only required testing on another 200 existing chemicals.
Increased risk in communities of color, recent immigrants, socially marginalized groups due to proximity to industrial sites, hazardous waste, pesticides in agricultural communities and diesel fumes in urban areas.
8

## Slide 9
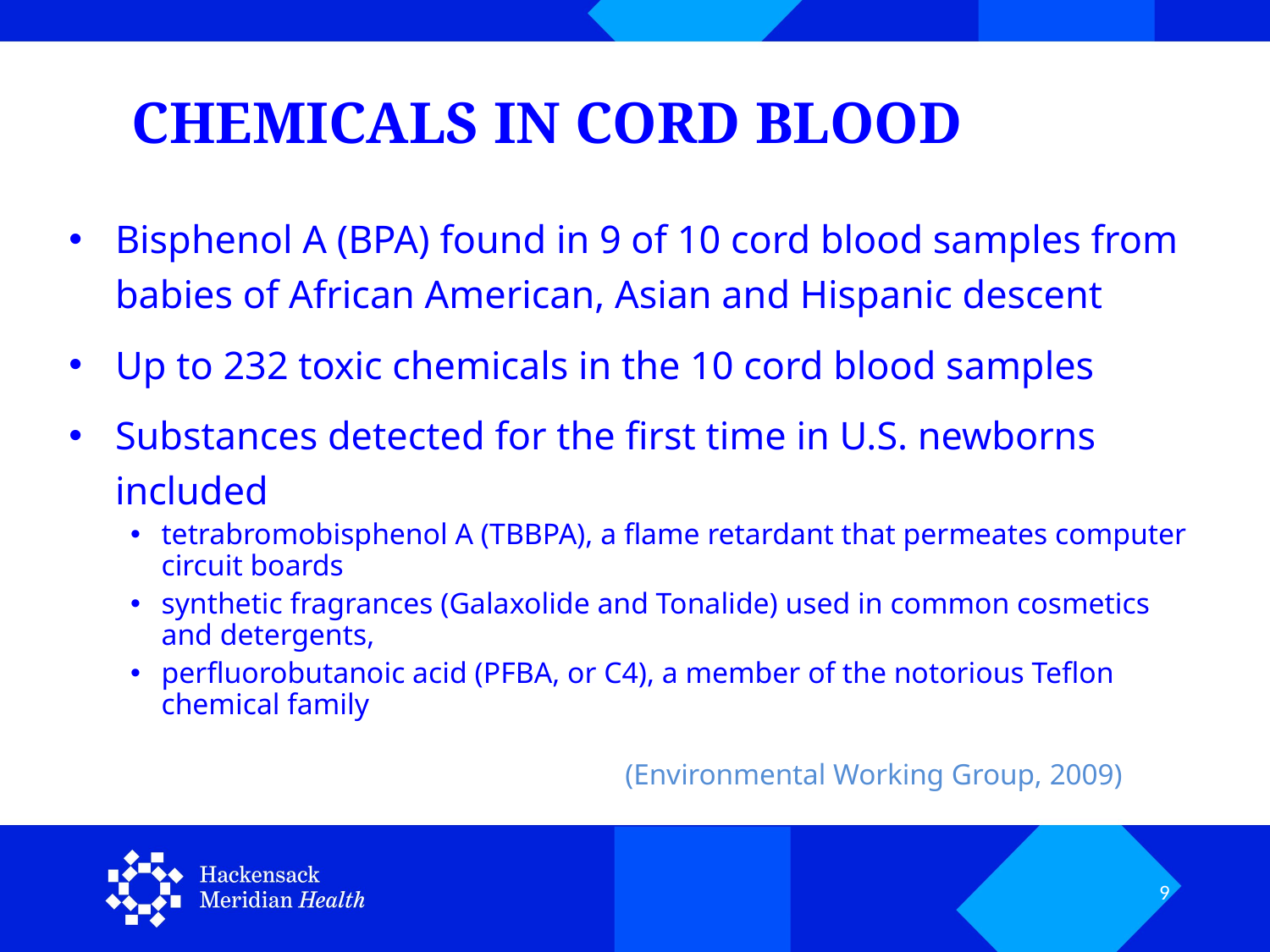

Chemicals in Cord blood
Bisphenol A (BPA) found in 9 of 10 cord blood samples from babies of African American, Asian and Hispanic descent
Up to 232 toxic chemicals in the 10 cord blood samples
Substances detected for the first time in U.S. newborns included
tetrabromobisphenol A (TBBPA), a flame retardant that permeates computer circuit boards
synthetic fragrances (Galaxolide and Tonalide) used in common cosmetics and detergents,
perfluorobutanoic acid (PFBA, or C4), a member of the notorious Teflon chemical family
		(Environmental Working Group, 2009)
9

## Slide 10
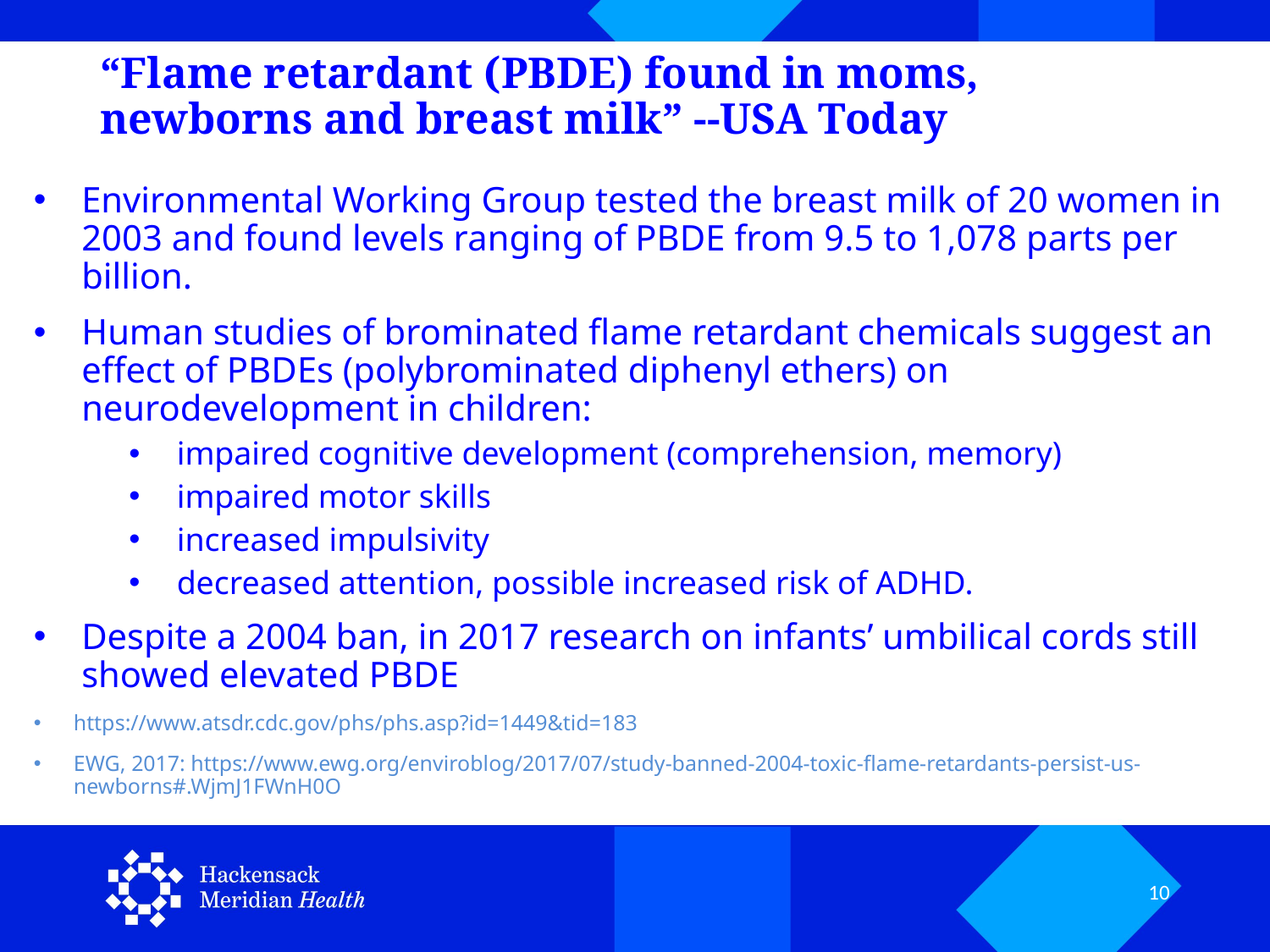

“Flame retardant (PBDE) found in moms, newborns and breast milk” --USA Today
Environmental Working Group tested the breast milk of 20 women in 2003 and found levels ranging of PBDE from 9.5 to 1,078 parts per billion.
Human studies of brominated flame retardant chemicals suggest an effect of PBDEs (polybrominated diphenyl ethers) on neurodevelopment in children:
impaired cognitive development (comprehension, memory)
impaired motor skills
increased impulsivity
decreased attention, possible increased risk of ADHD.
Despite a 2004 ban, in 2017 research on infants’ umbilical cords still showed elevated PBDE
https://www.atsdr.cdc.gov/phs/phs.asp?id=1449&tid=183
EWG, 2017: https://www.ewg.org/enviroblog/2017/07/study-banned-2004-toxic-flame-retardants-persist-us-newborns#.WjmJ1FWnH0O
10

## Slide 11
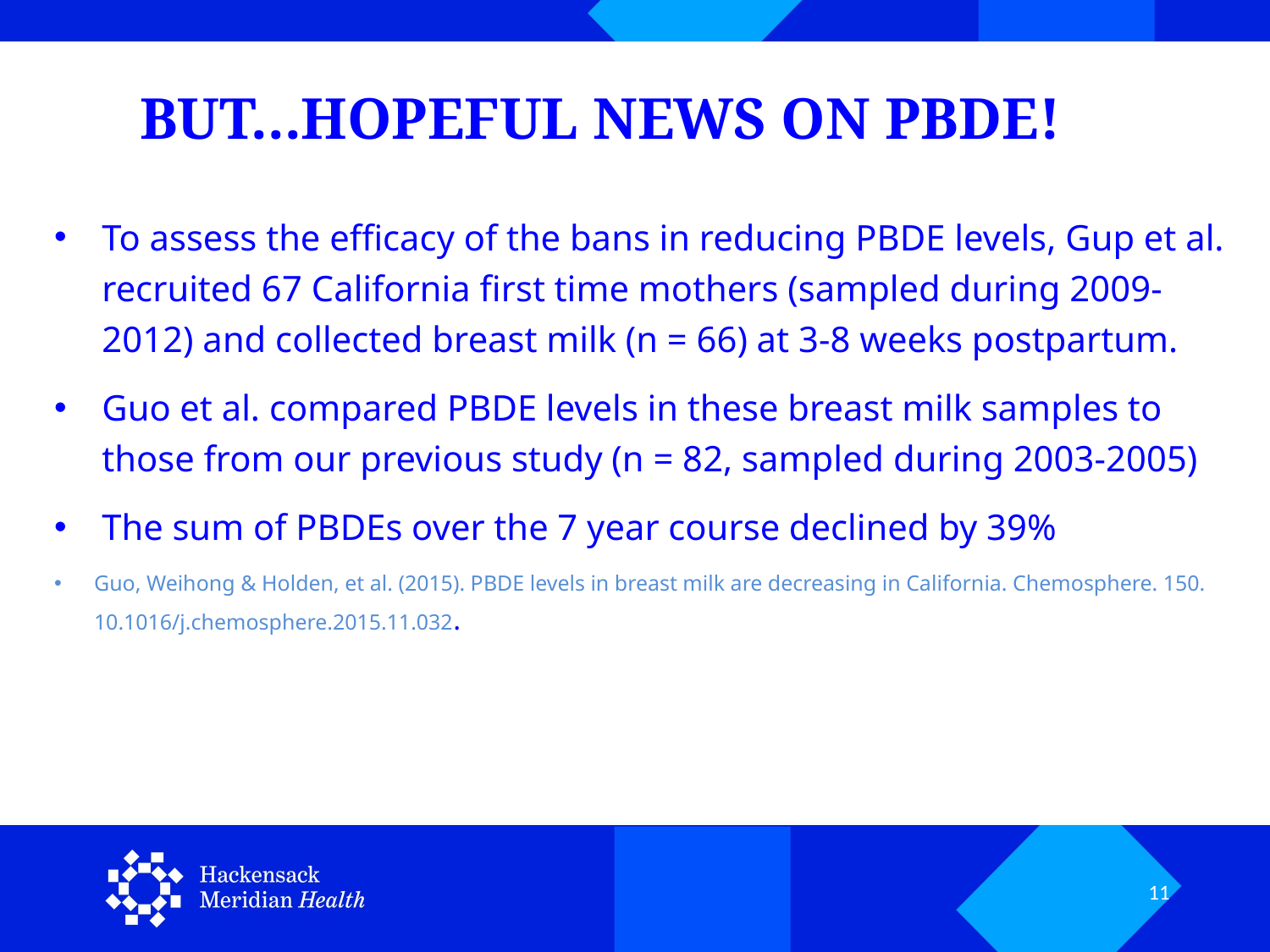

# BUT...HOPEFUL NEWS ON PBDE!
To assess the efficacy of the bans in reducing PBDE levels, Gup et al. recruited 67 California first time mothers (sampled during 2009-2012) and collected breast milk (n = 66) at 3-8 weeks postpartum.
Guo et al. compared PBDE levels in these breast milk samples to those from our previous study (n = 82, sampled during 2003-2005)
The sum of PBDEs over the 7 year course declined by 39%
Guo, Weihong & Holden, et al. (2015). PBDE levels in breast milk are decreasing in California. Chemosphere. 150. 10.1016/j.chemosphere.2015.11.032.
11

## Slide 12
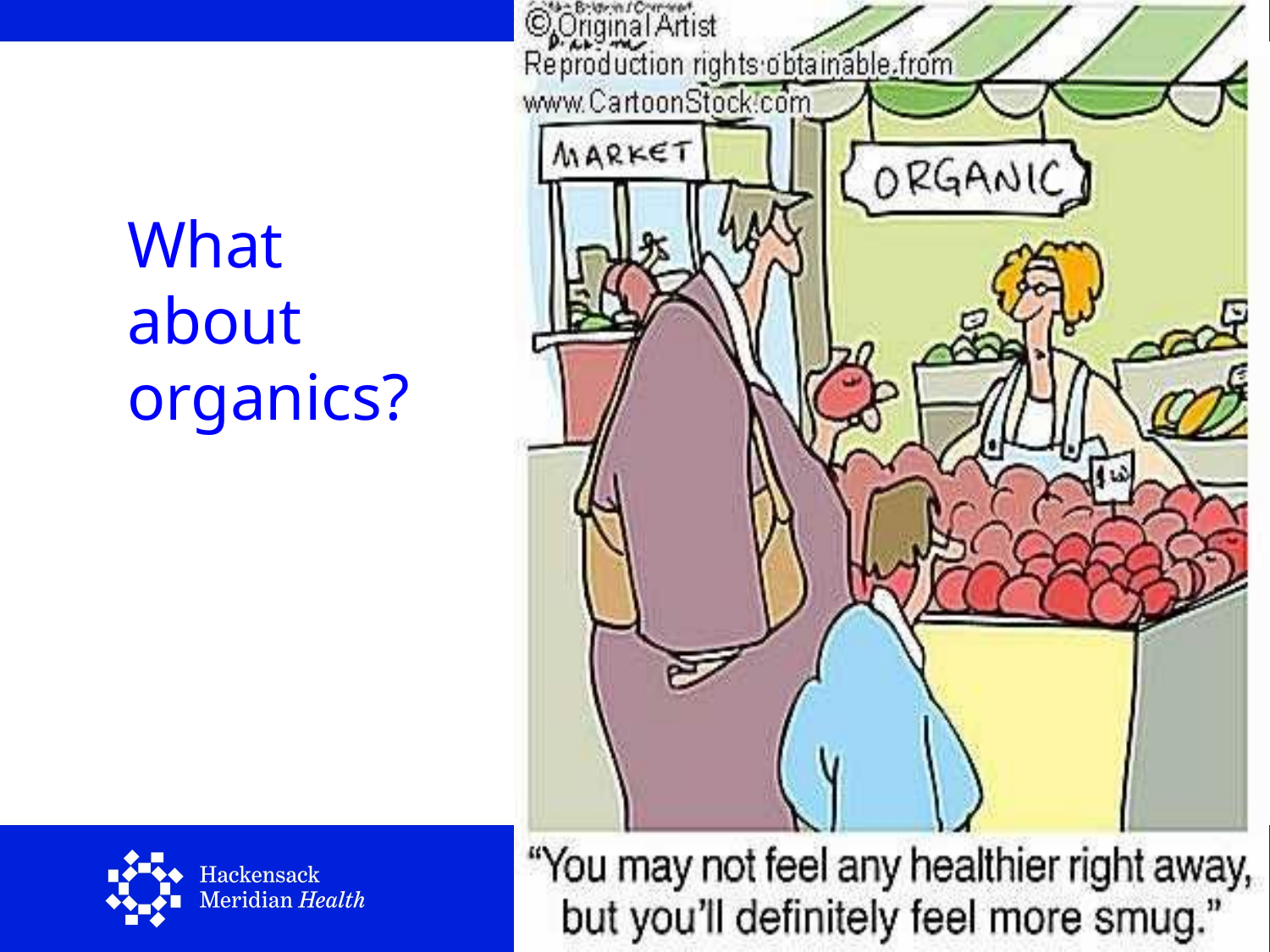

What
about
organics?
12

## Slide 13
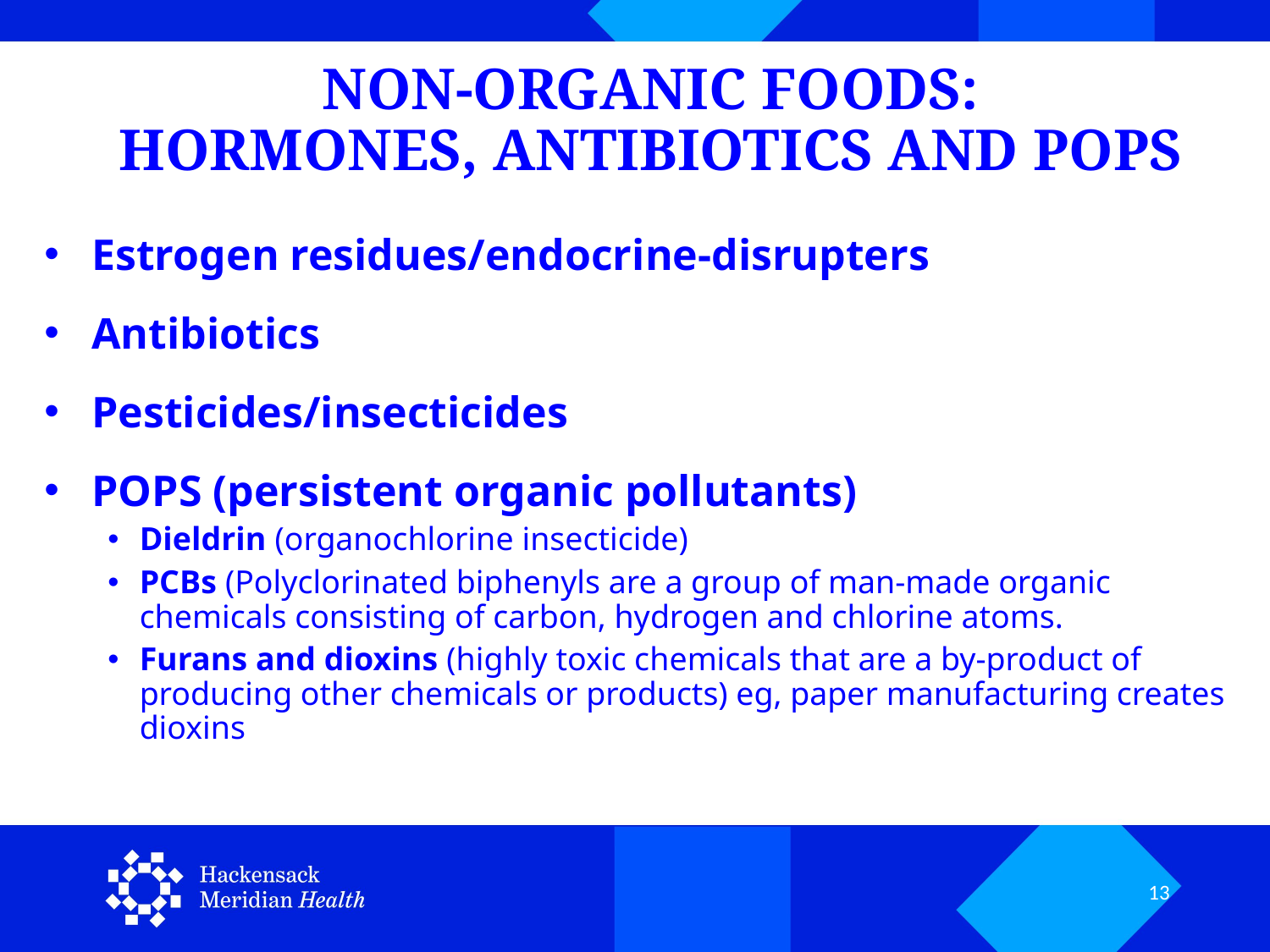

NON-ORGANIC FOODS:
HORMONES, ANTIBIOTICS AND POPS
Estrogen residues/endocrine-disrupters
Antibiotics
Pesticides/insecticides
POPS (persistent organic pollutants)
Dieldrin (organochlorine insecticide)
PCBs (Polyclorinated biphenyls are a group of man-made organic chemicals consisting of carbon, hydrogen and chlorine atoms.
Furans and dioxins (highly toxic chemicals that are a by-product of producing other chemicals or products) eg, paper manufacturing creates dioxins
13

## Slide 14
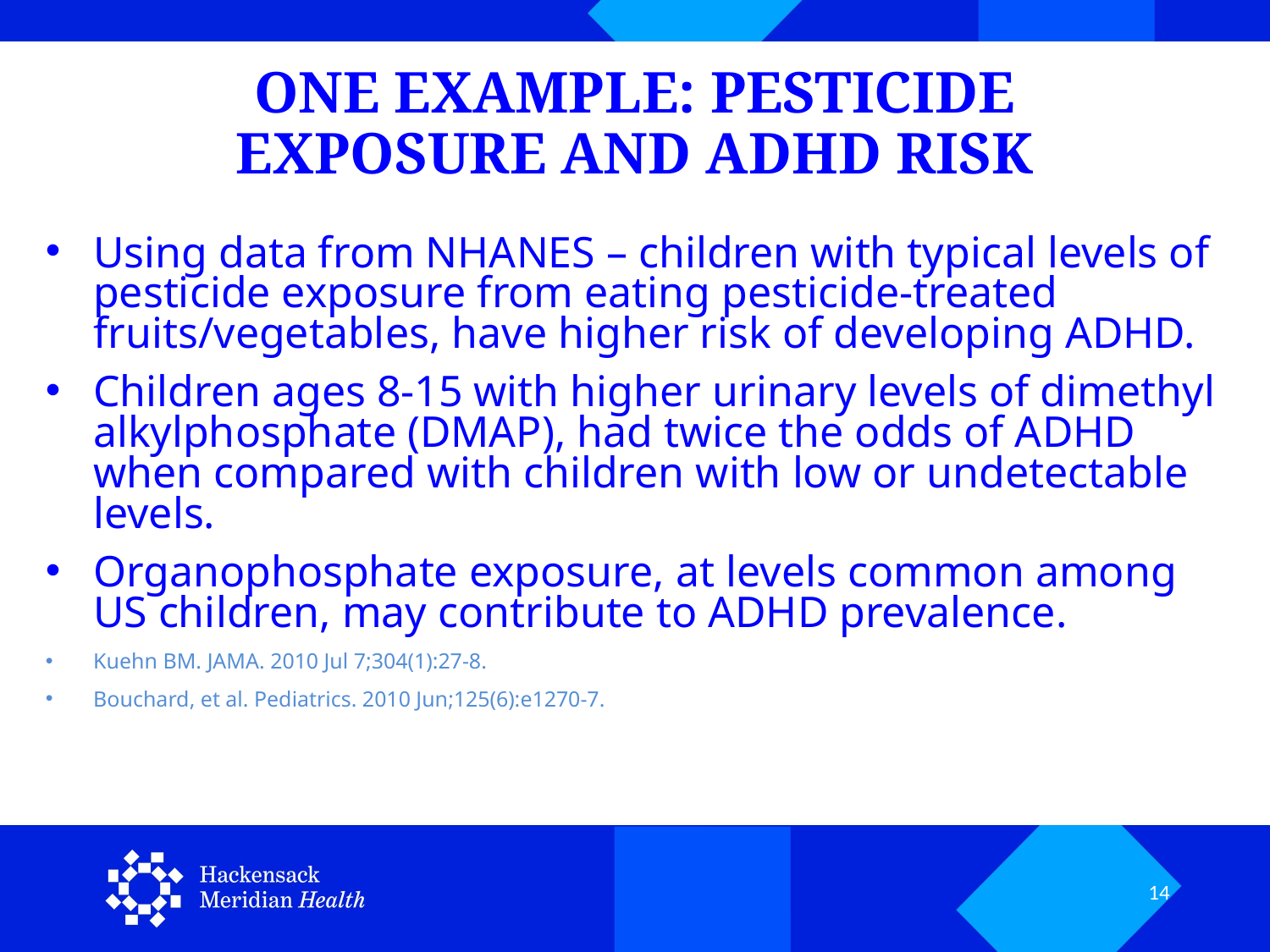

ONE EXAMPLE: PESTICIDE EXPOSURE AND ADHD RISK
Using data from NHANES – children with typical levels of pesticide exposure from eating pesticide-treated fruits/vegetables, have higher risk of developing ADHD.
Children ages 8-15 with higher urinary levels of dimethyl alkylphosphate (DMAP), had twice the odds of ADHD when compared with children with low or undetectable levels.
Organophosphate exposure, at levels common among US children, may contribute to ADHD prevalence.
Kuehn BM. JAMA. 2010 Jul 7;304(1):27‐8.
Bouchard, et al. Pediatrics. 2010 Jun;125(6):e1270‐7.
14

## Slide 15
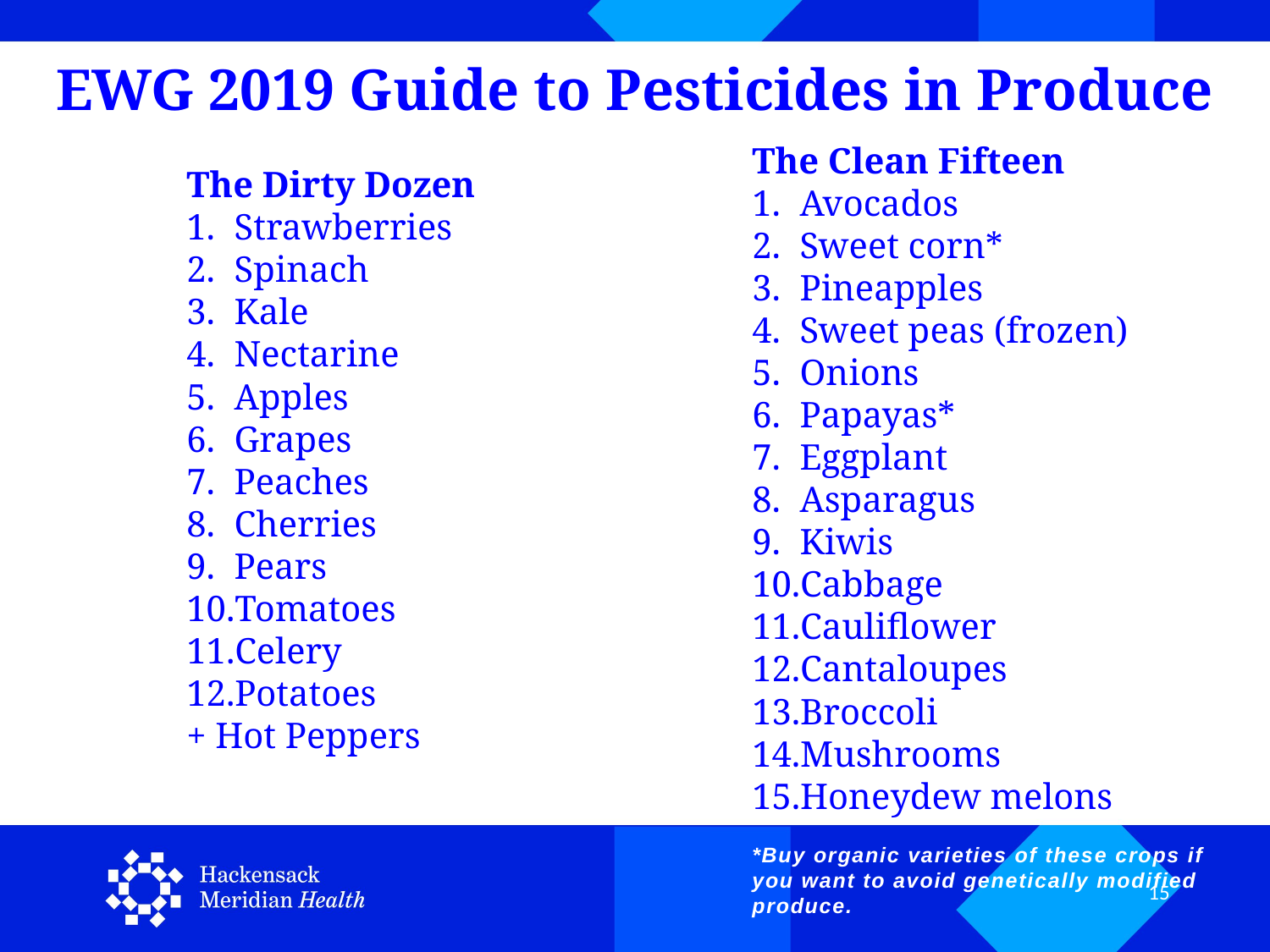

EWG 2019 Guide to Pesticides in Produce
The Clean Fifteen
Avocados
Sweet corn*
Pineapples
Sweet peas (frozen)
Onions
Papayas*
Eggplant
Asparagus
Kiwis
Cabbage
Cauliflower
Cantaloupes
Broccoli
Mushrooms
Honeydew melons
*Buy organic varieties of these crops if you want to avoid genetically modified produce.
The Dirty Dozen
Strawberries
Spinach
Kale
Nectarine
Apples
Grapes
Peaches
Cherries
Pears
Tomatoes
Celery
Potatoes
+ Hot Peppers
15

## Slide 16
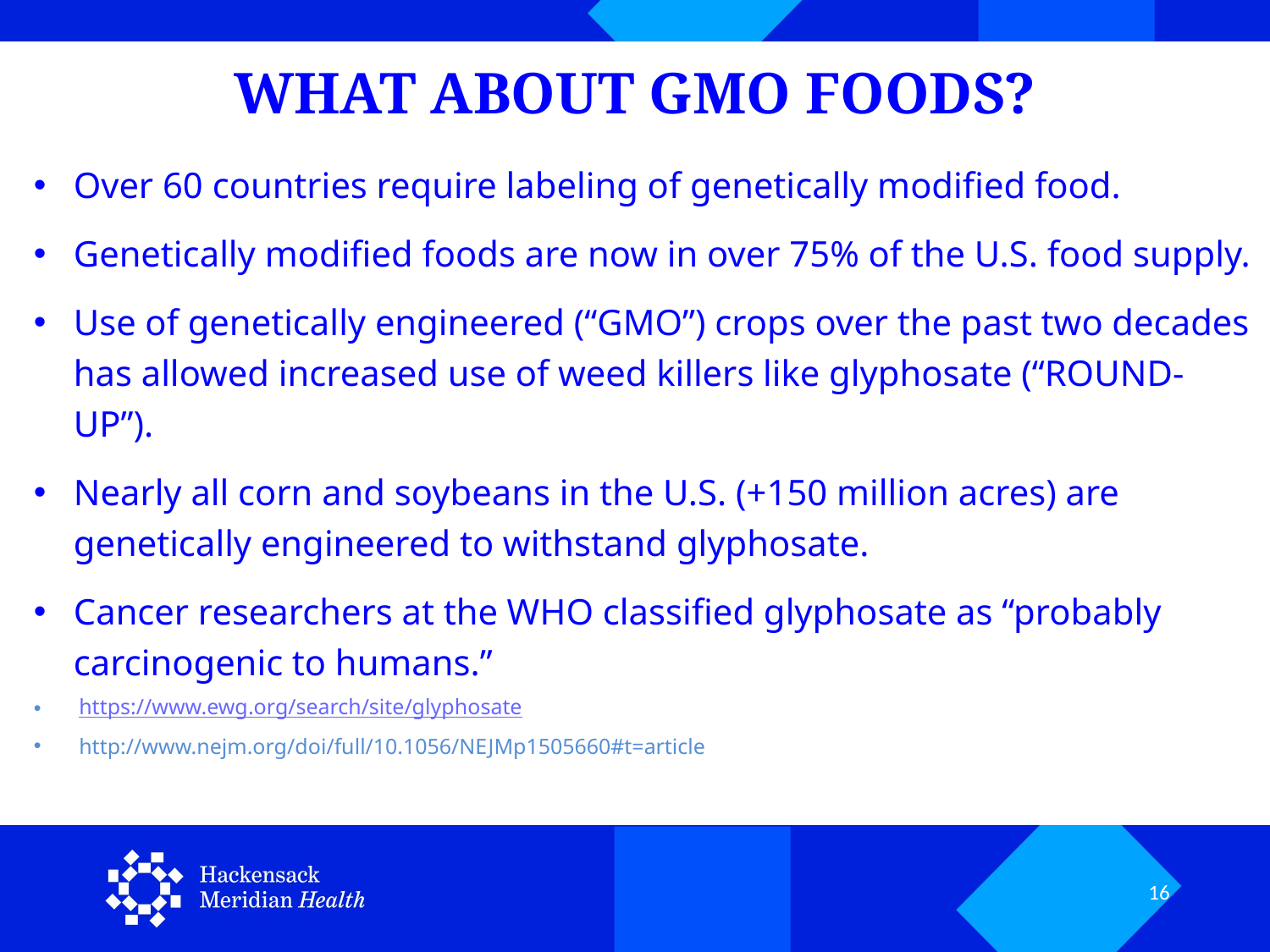

WHAT ABOUT GMO FOODS?
Over 60 countries require labeling of genetically modified food.
Genetically modified foods are now in over 75% of the U.S. food supply.
Use of genetically engineered (“GMO”) crops over the past two decades has allowed increased use of weed killers like glyphosate (“ROUND-UP”).
Nearly all corn and soybeans in the U.S. (+150 million acres) are genetically engineered to withstand glyphosate.
Cancer researchers at the WHO classified glyphosate as “probably carcinogenic to humans.”
 https://www.ewg.org/search/site/glyphosate
 http://www.nejm.org/doi/full/10.1056/NEJMp1505660#t=article
16

## Slide 17
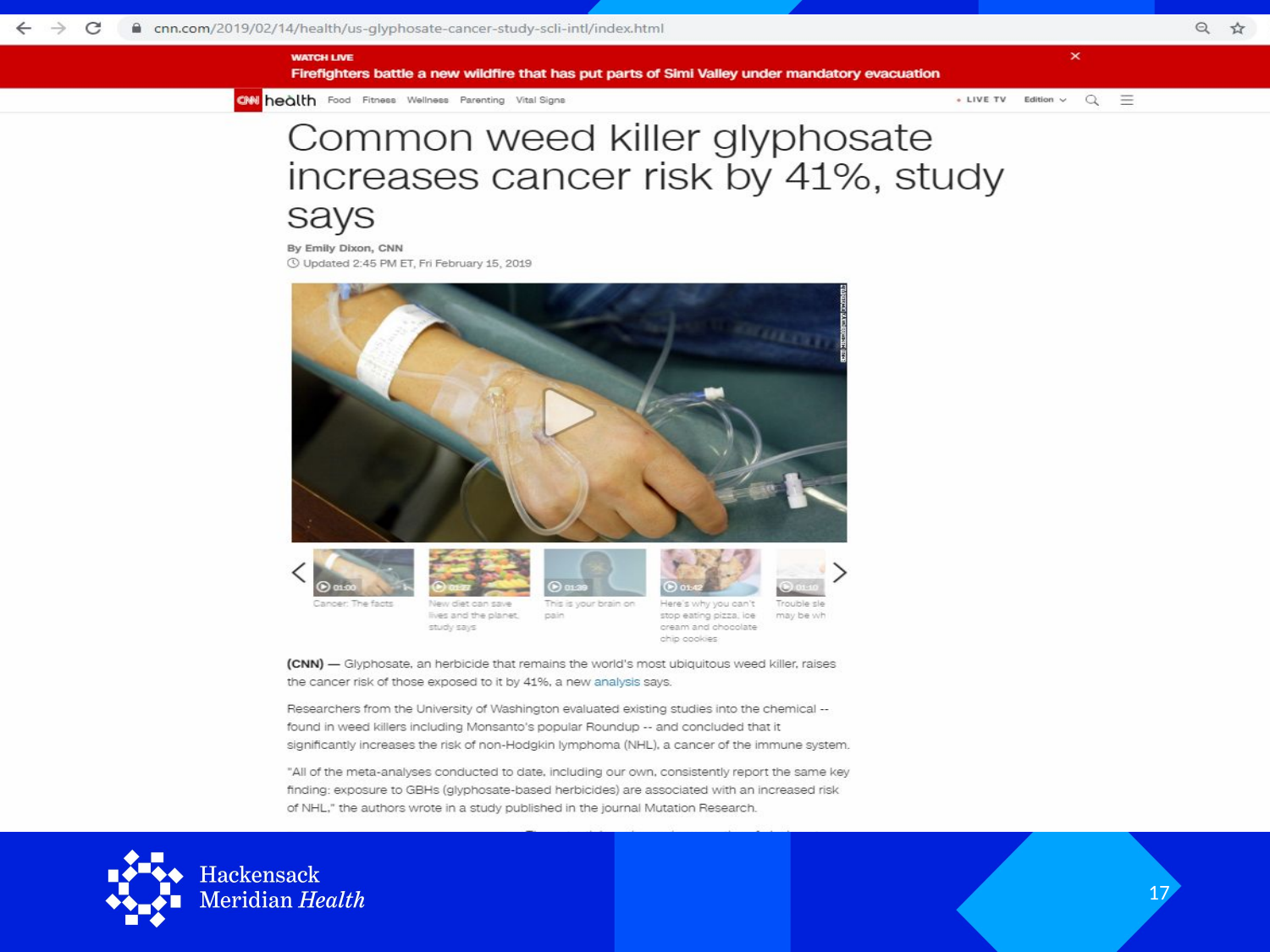

17

## Slide 18
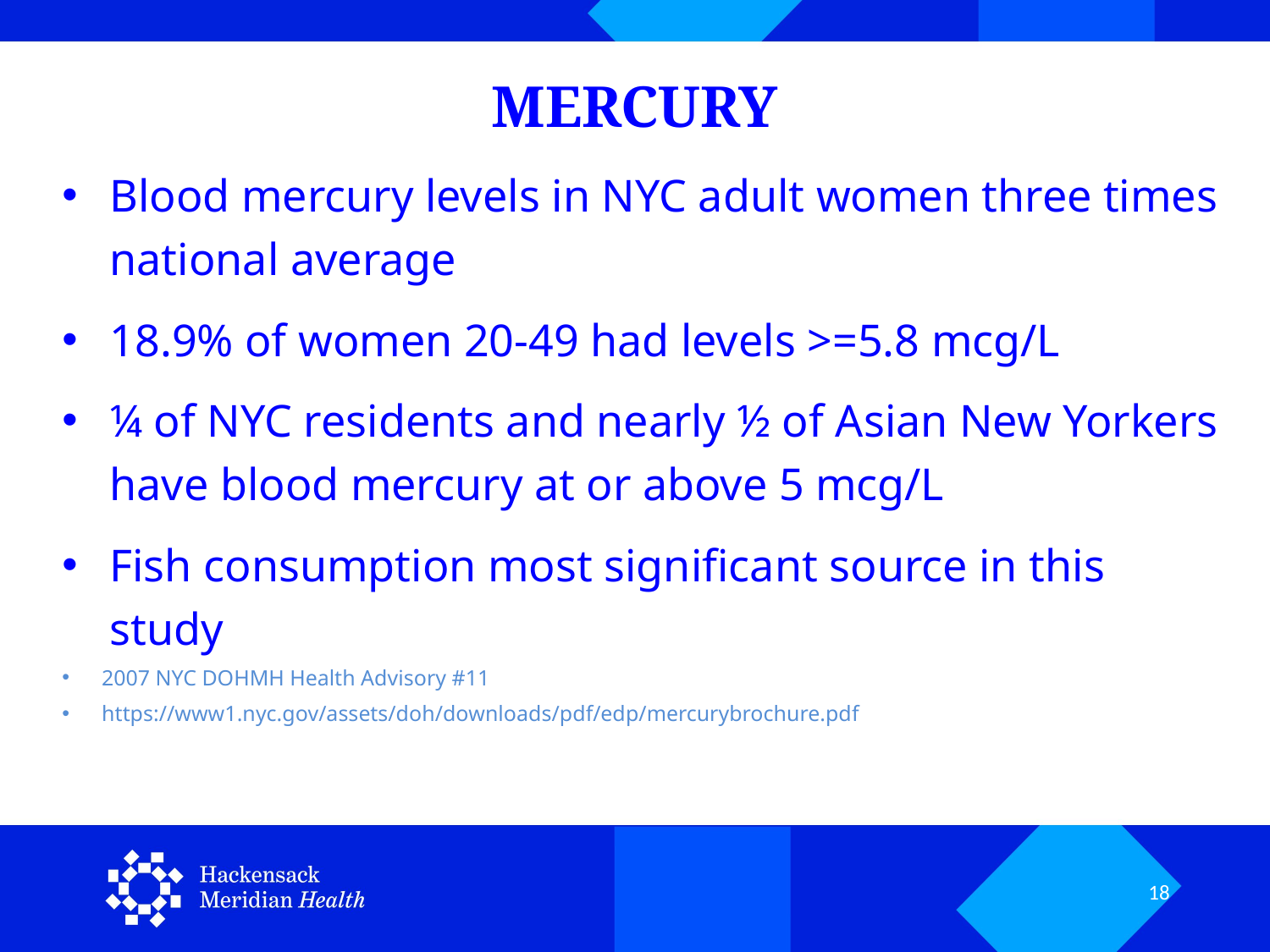

MERCURY
Blood mercury levels in NYC adult women three times national average
18.9% of women 20-49 had levels >=5.8 mcg/L
¼ of NYC residents and nearly ½ of Asian New Yorkers have blood mercury at or above 5 mcg/L
Fish consumption most significant source in this study
2007 NYC DOHMH Health Advisory #11
https://www1.nyc.gov/assets/doh/downloads/pdf/edp/mercurybrochure.pdf
18

## Slide 19
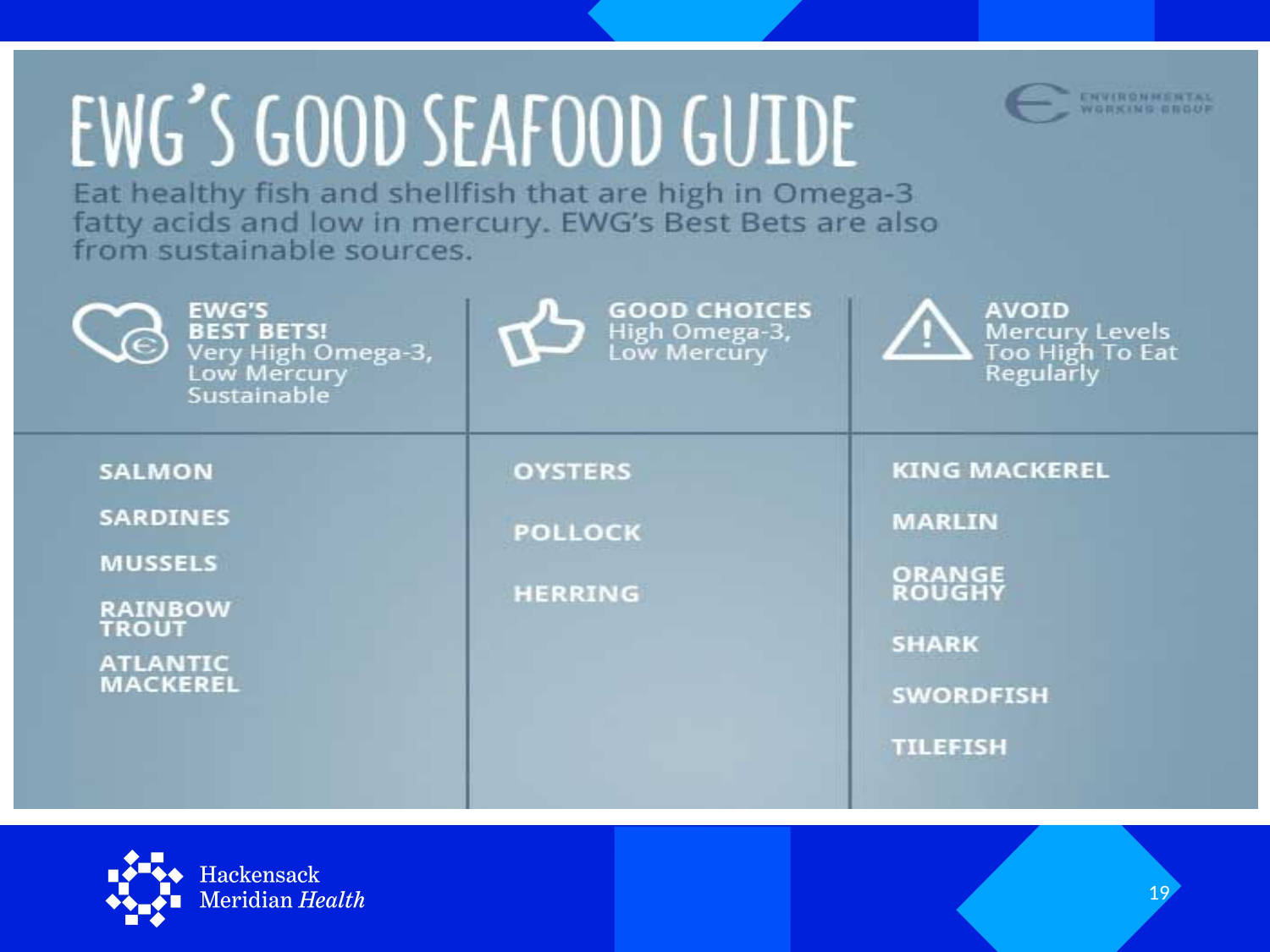

19

## Slide 20
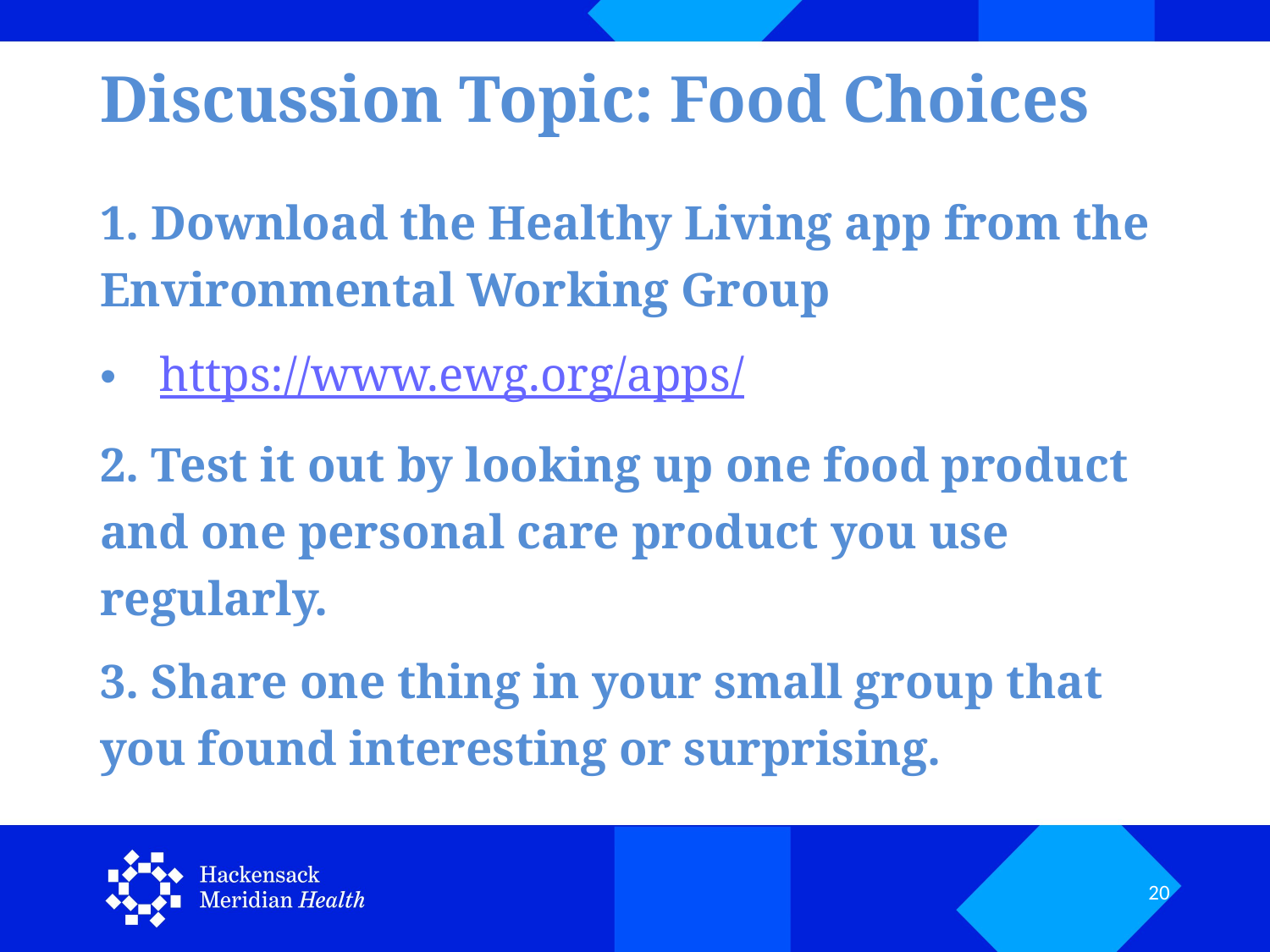

# Discussion Topic: Food Choices
1. Download the Healthy Living app from the Environmental Working Group
https://www.ewg.org/apps/
2. Test it out by looking up one food product and one personal care product you use regularly.
3. Share one thing in your small group that you found interesting or surprising.
20

## Slide 21
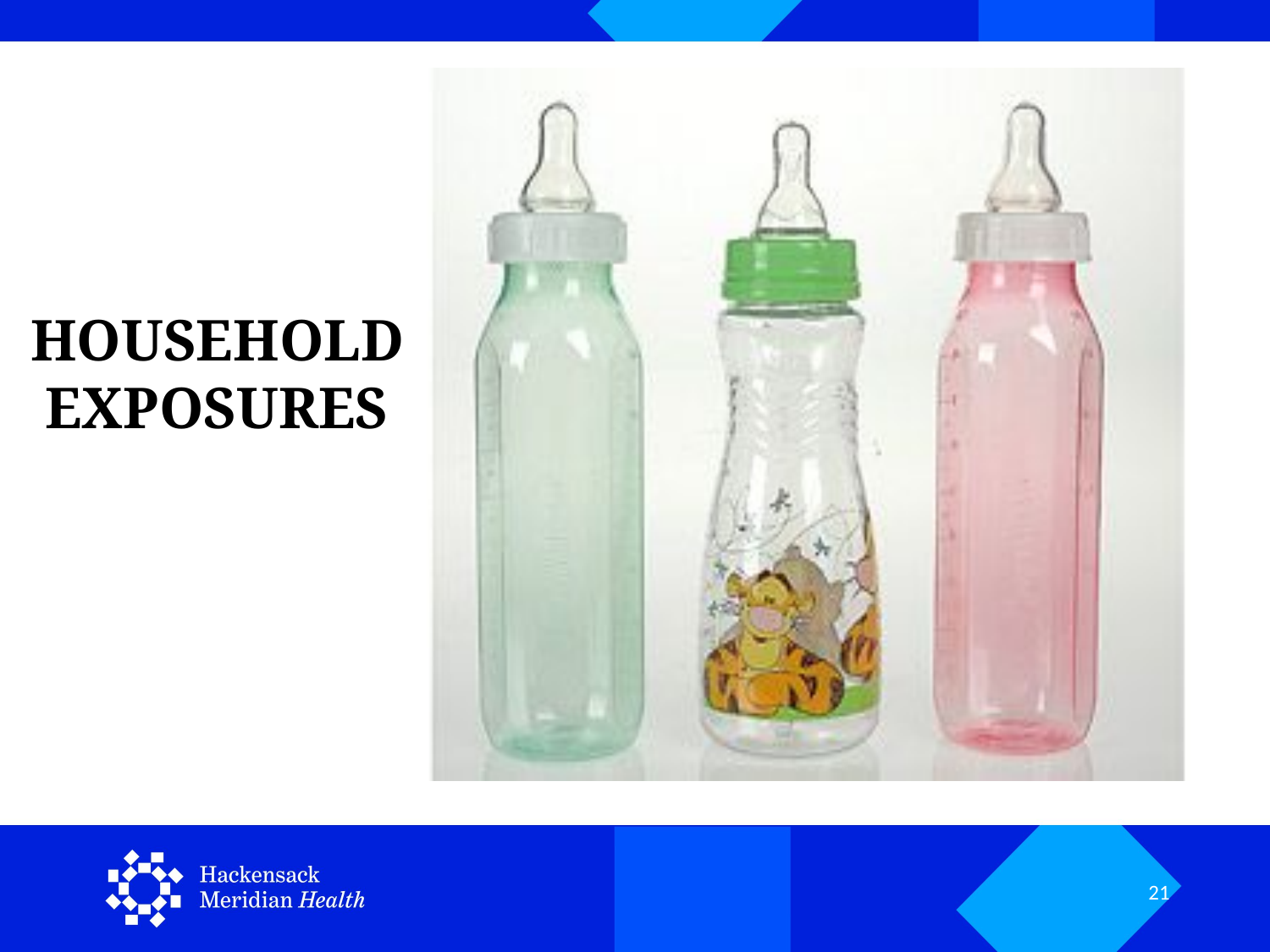

HOUSEHOLD
 EXPOSURES
21

## Slide 22
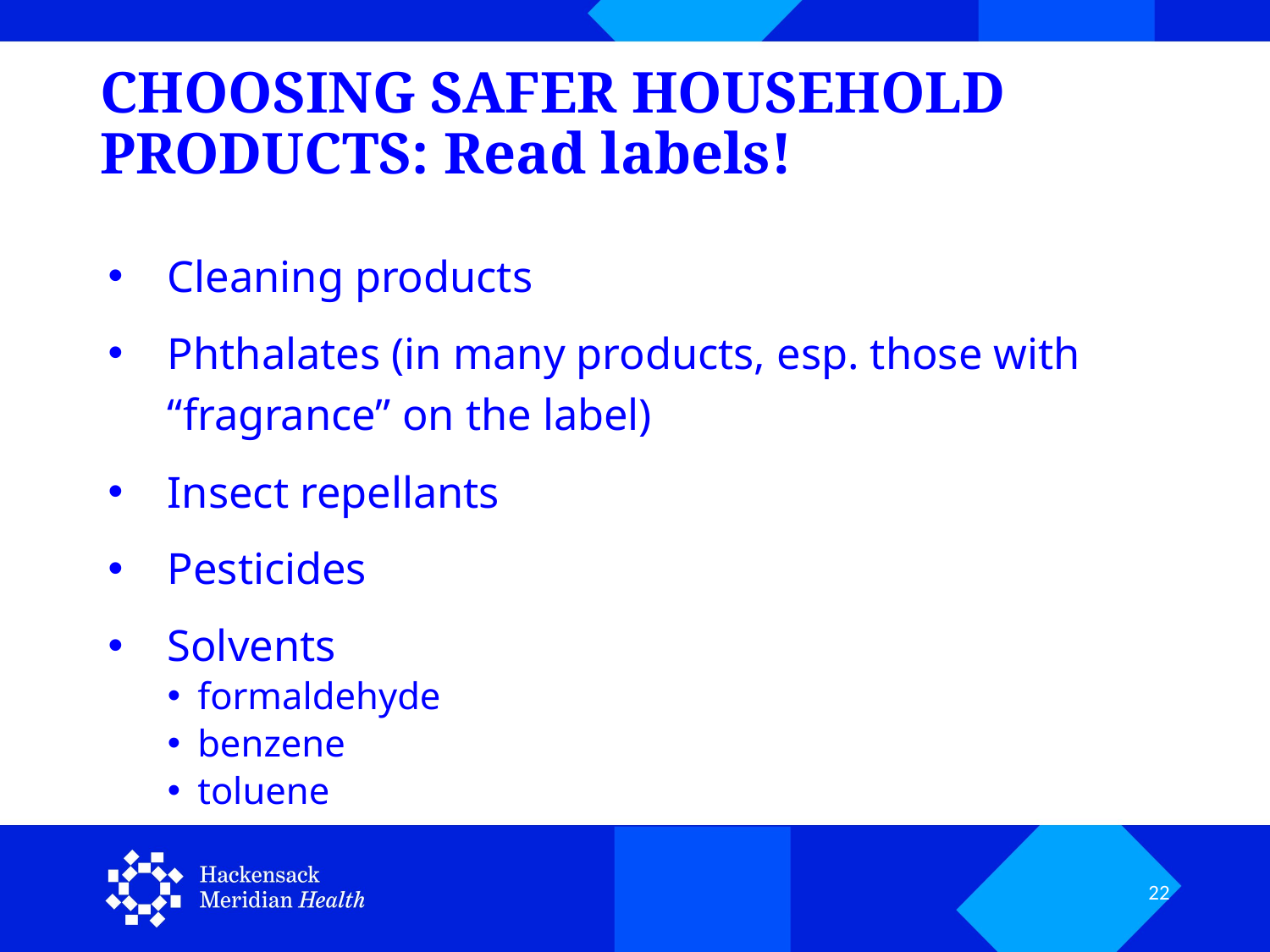

CHOOSING SAFER HOUSEHOLD PRODUCTS: Read labels!
Cleaning products
Phthalates (in many products, esp. those with “fragrance” on the label)
Insect repellants
Pesticides
Solvents
formaldehyde
benzene
toluene
22

## Slide 23
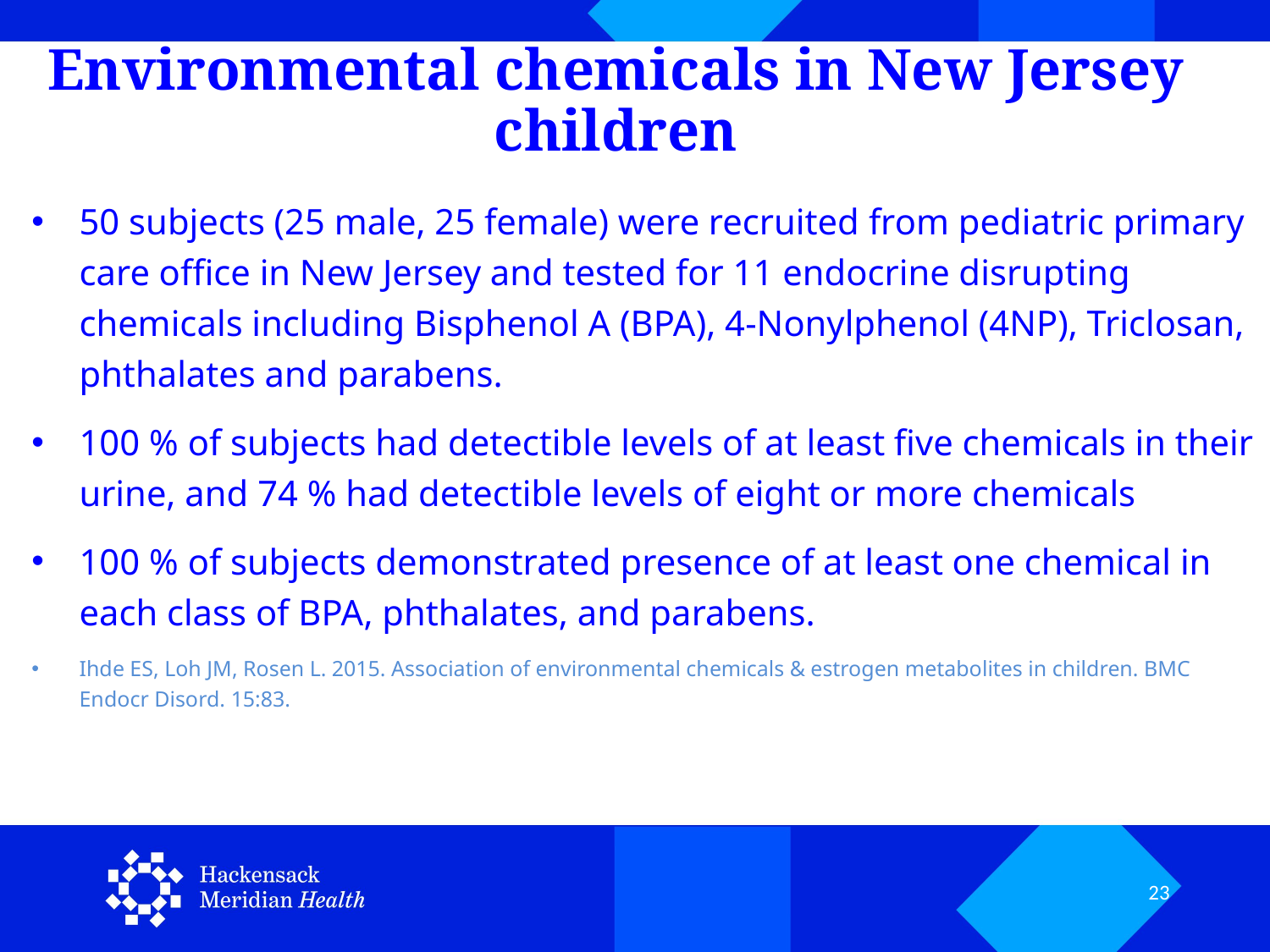

Environmental chemicals in New Jersey children
50 subjects (25 male, 25 female) were recruited from pediatric primary care office in New Jersey and tested for 11 endocrine disrupting chemicals including Bisphenol A (BPA), 4-Nonylphenol (4NP), Triclosan, phthalates and parabens.
100 % of subjects had detectible levels of at least five chemicals in their urine, and 74 % had detectible levels of eight or more chemicals
100 % of subjects demonstrated presence of at least one chemical in each class of BPA, phthalates, and parabens.
Ihde ES, Loh JM, Rosen L. 2015. Association of environmental chemicals & estrogen metabolites in children. BMC Endocr Disord. 15:83.
23

## Slide 24
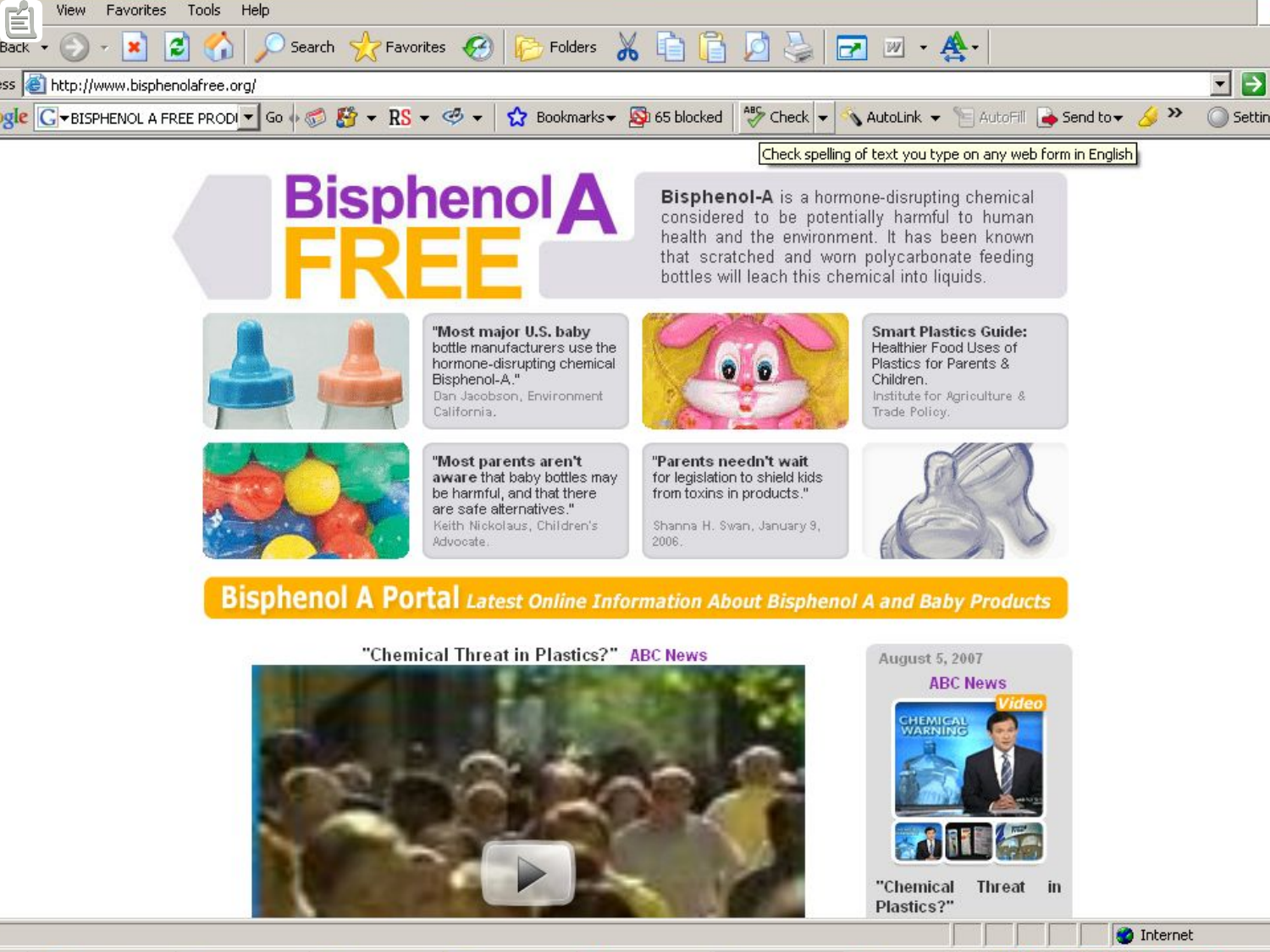

24

## Slide 25
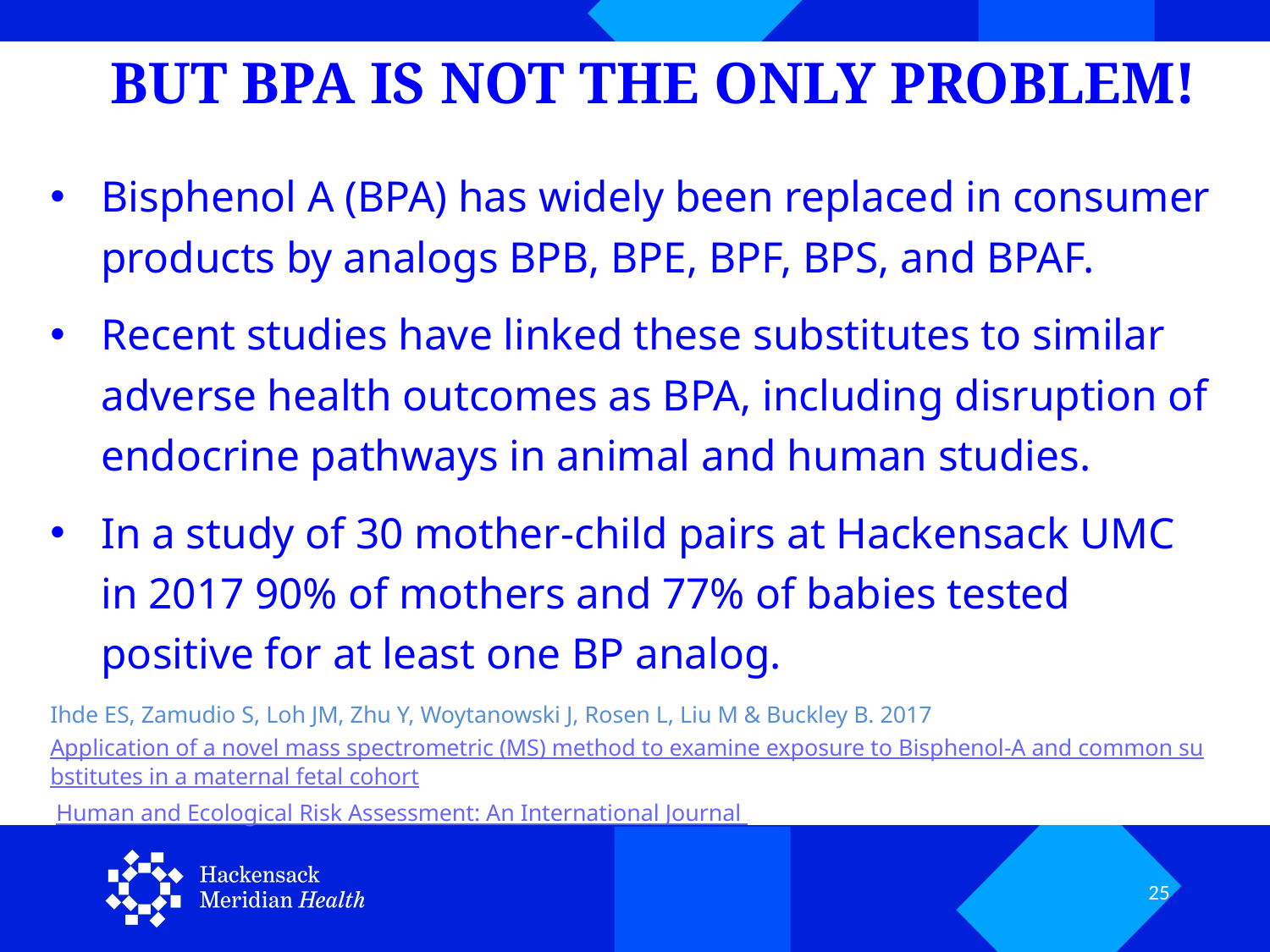

BUT BPA IS NOT THE ONLY PROBLEM!
Bisphenol A (BPA) has widely been replaced in consumer products by analogs BPB, BPE, BPF, BPS, and BPAF.
Recent studies have linked these substitutes to similar adverse health outcomes as BPA, including disruption of endocrine pathways in animal and human studies.
In a study of 30 mother-child pairs at Hackensack UMC in 2017 90% of mothers and 77% of babies tested positive for at least one BP analog.
Ihde ES, Zamudio S, Loh JM, Zhu Y, Woytanowski J, Rosen L, Liu M & Buckley B. 2017Application of a novel mass spectrometric (MS) method to examine exposure to Bisphenol-A and common substitutes in a maternal fetal cohort Human and Ecological Risk Assessment: An International Journal
25

## Slide 26
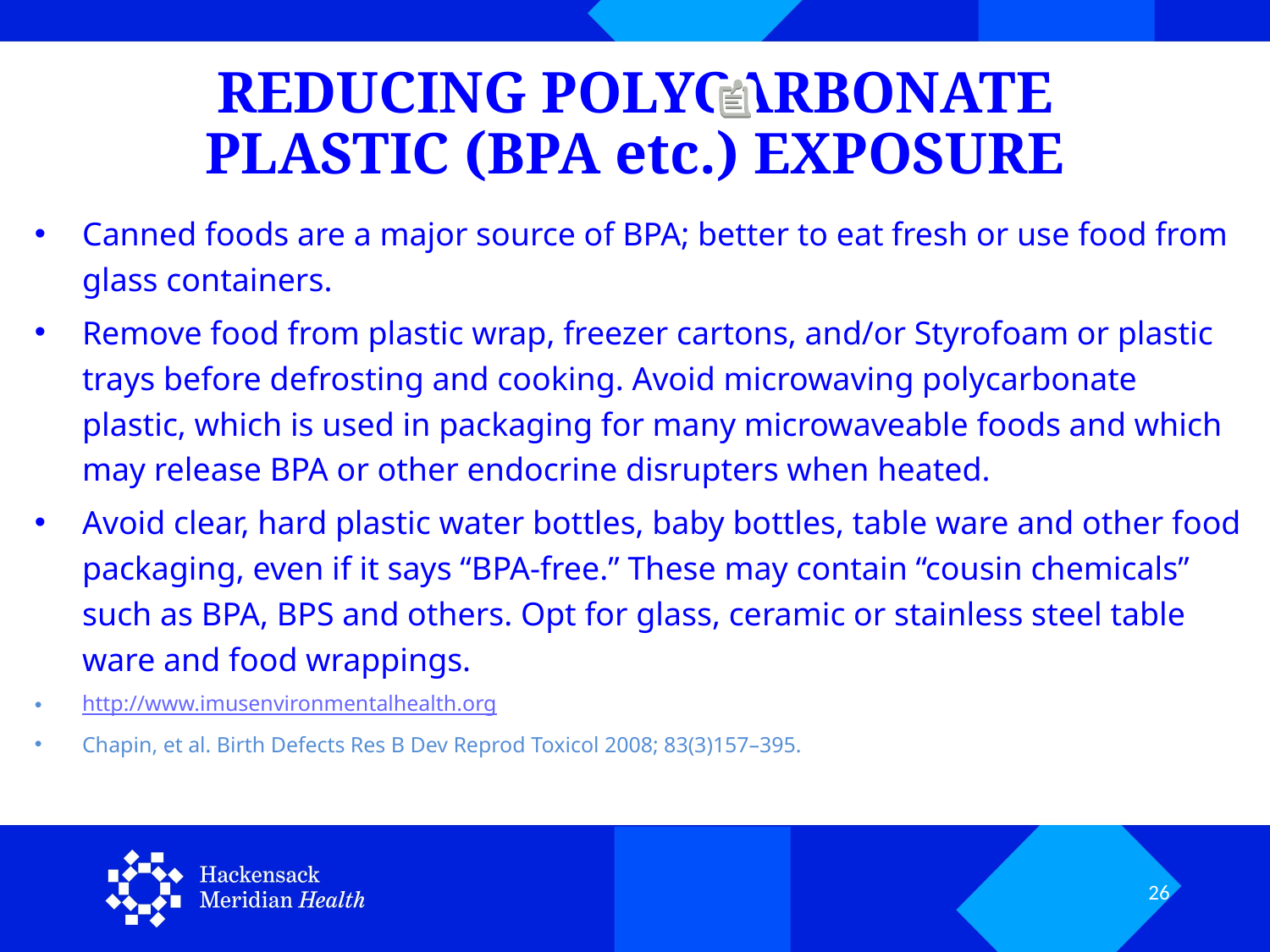

REDUCING POLYCARBONATE PLASTIC (BPA etc.) EXPOSURE
Canned foods are a major source of BPA; better to eat fresh or use food from glass containers.
Remove food from plastic wrap, freezer cartons, and/or Styrofoam or plastic trays before defrosting and cooking. Avoid microwaving polycarbonate plastic, which is used in packaging for many microwaveable foods and which may release BPA or other endocrine disrupters when heated.
Avoid clear, hard plastic water bottles, baby bottles, table ware and other food packaging, even if it says “BPA-free.” These may contain “cousin chemicals” such as BPA, BPS and others. Opt for glass, ceramic or stainless steel table ware and food wrappings.
http://www.imusenvironmentalhealth.org
Chapin, et al. Birth Defects Res B Dev Reprod Toxicol 2008; 83(3)157–395.
26

## Slide 27
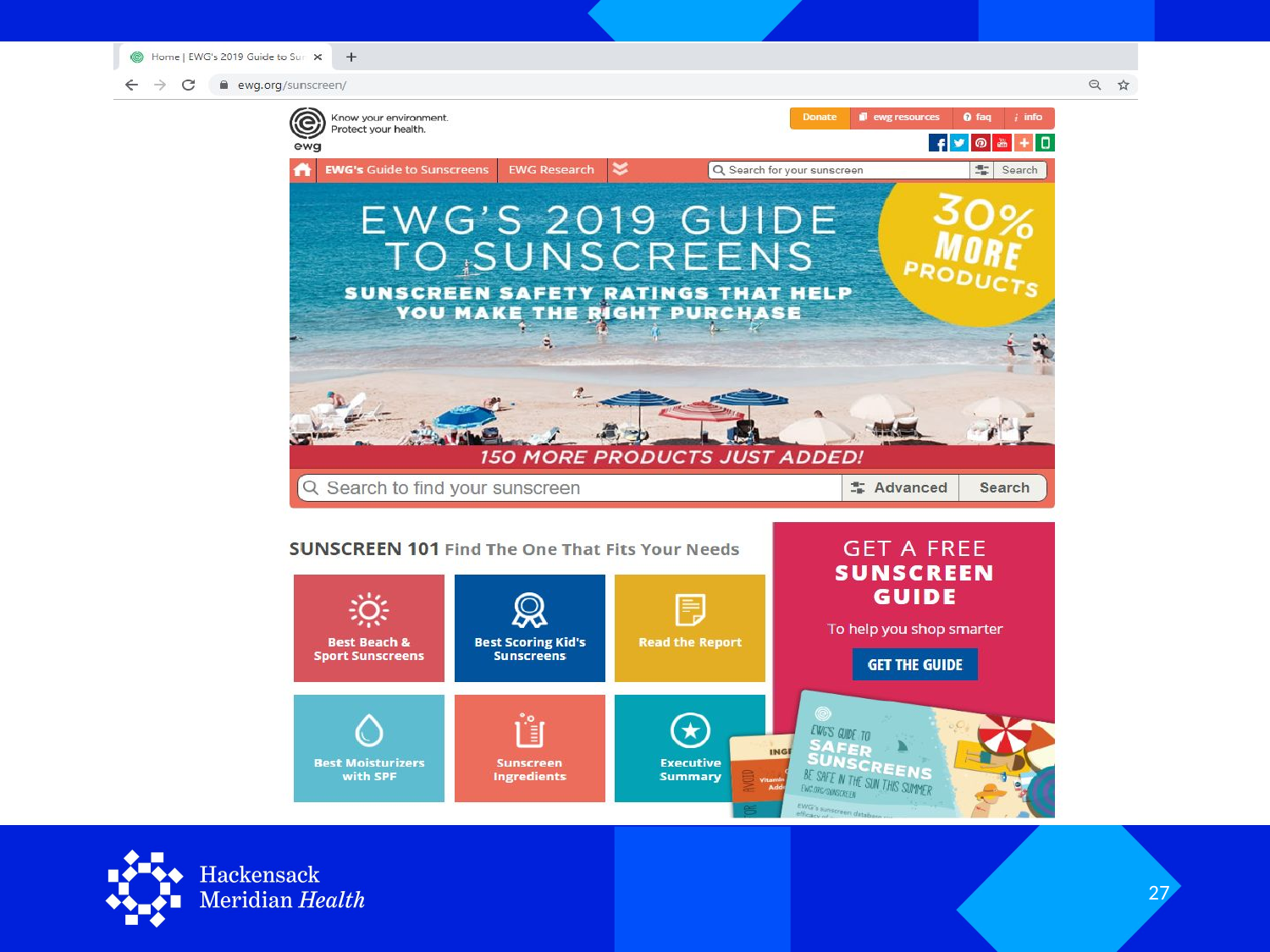

27

## Slide 28
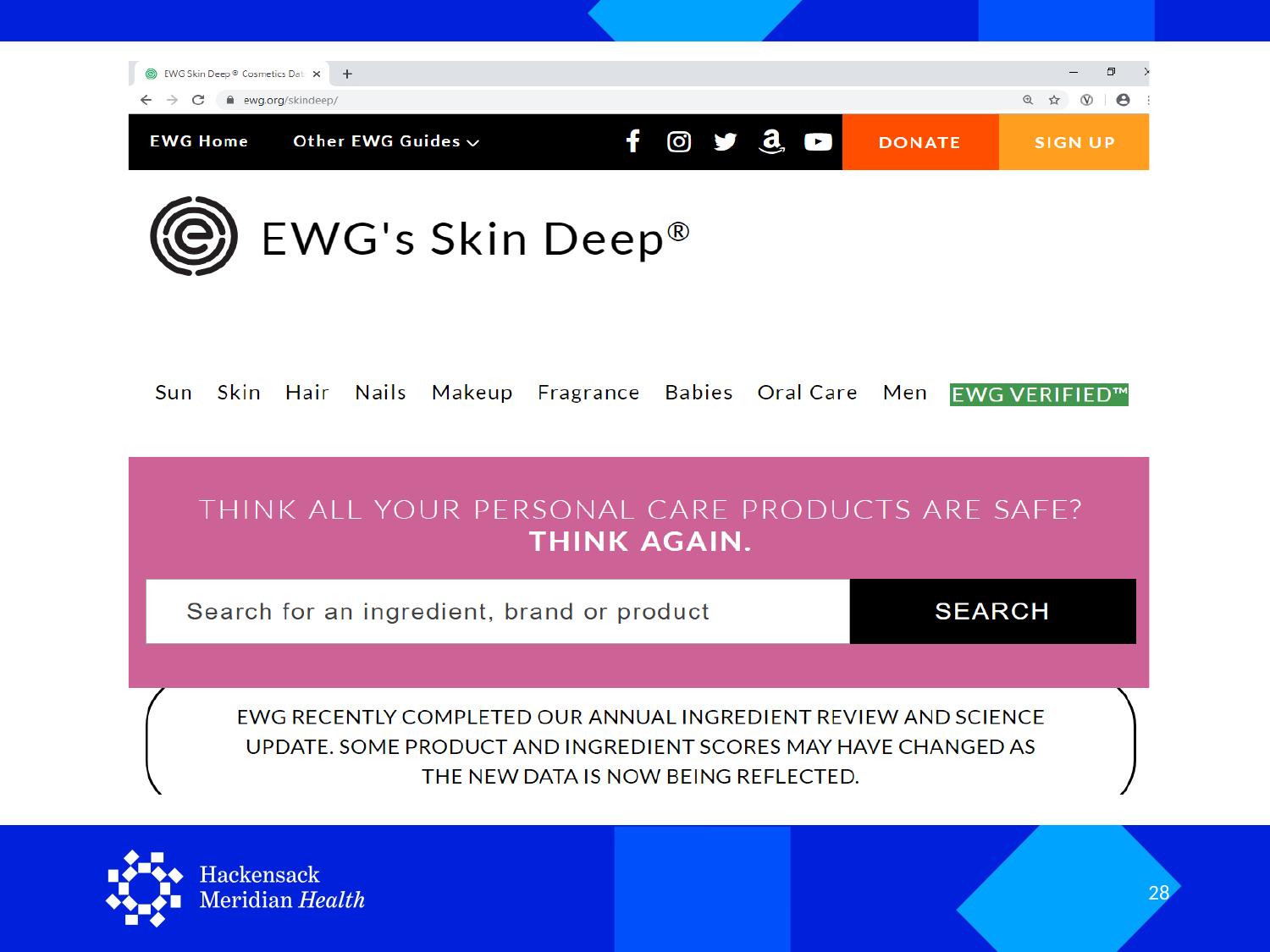

28

## Slide 29
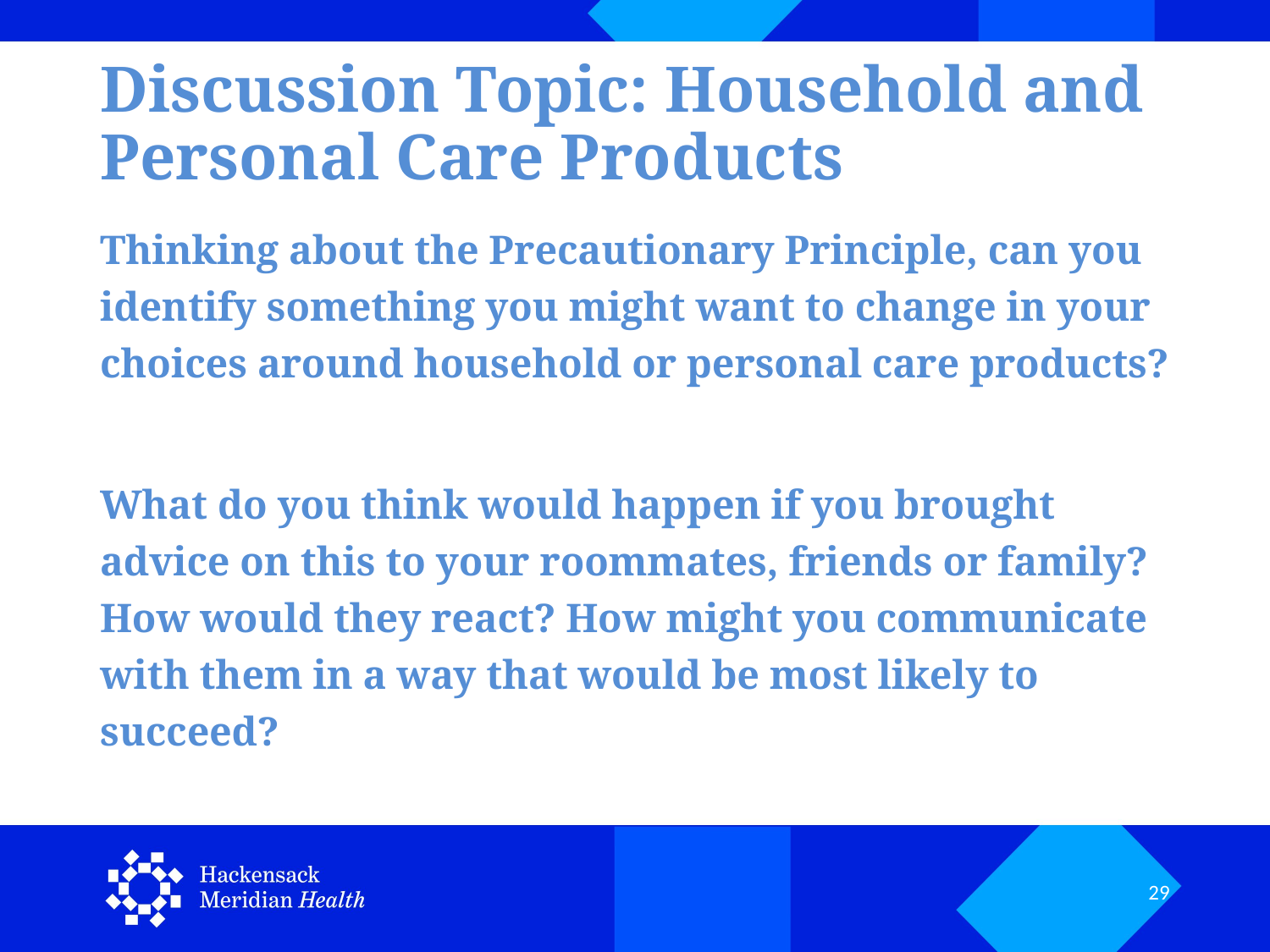

# Discussion Topic: Household and Personal Care Products
Thinking about the Precautionary Principle, can you identify something you might want to change in your choices around household or personal care products?
What do you think would happen if you brought advice on this to your roommates, friends or family? How would they react? How might you communicate with them in a way that would be most likely to succeed?
29

## Slide 30
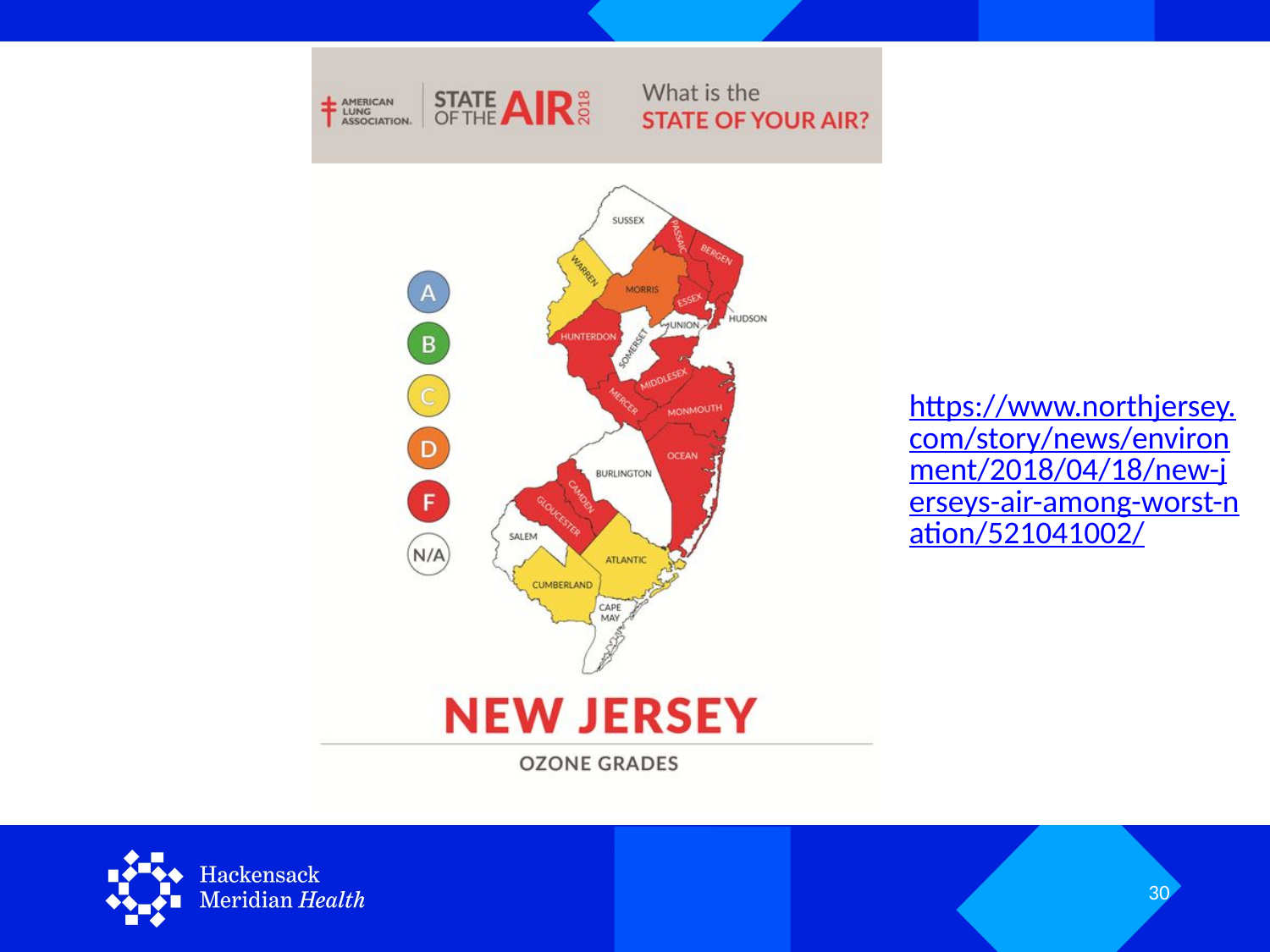

https://www.northjersey.com/story/news/environment/2018/04/18/new-jerseys-air-among-worst-nation/521041002/
30

## Slide 31
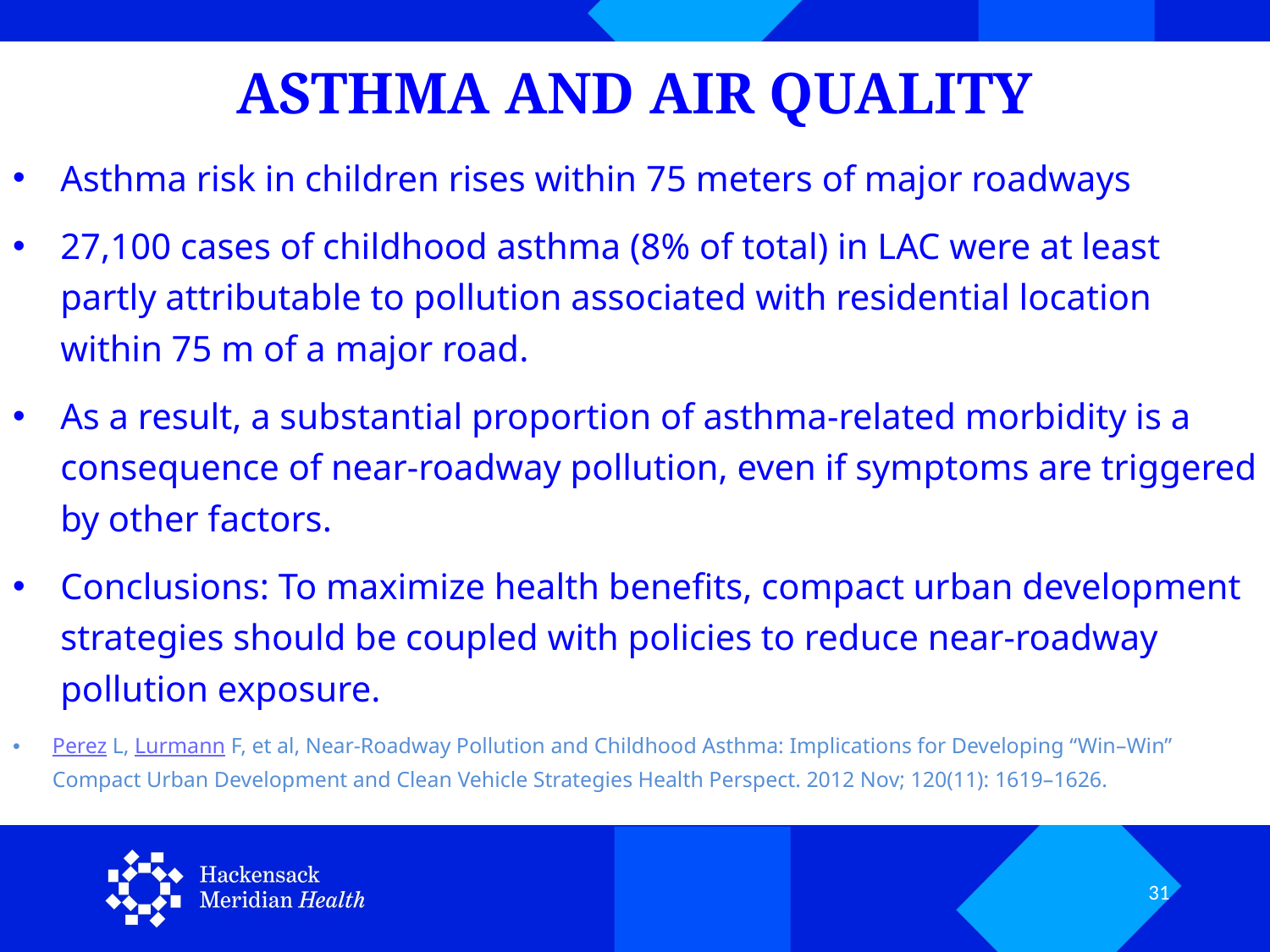

# ASTHMA AND AIR QUALITY
Asthma risk in children rises within 75 meters of major roadways
27,100 cases of childhood asthma (8% of total) in LAC were at least partly attributable to pollution associated with residential location within 75 m of a major road.
As a result, a substantial proportion of asthma-related morbidity is a consequence of near-roadway pollution, even if symptoms are triggered by other factors.
Conclusions: To maximize health benefits, compact urban development strategies should be coupled with policies to reduce near-roadway pollution exposure.
Perez L, Lurmann F, et al, Near-Roadway Pollution and Childhood Asthma: Implications for Developing “Win–Win” Compact Urban Development and Clean Vehicle Strategies Health Perspect. 2012 Nov; 120(11): 1619–1626.
31

## Slide 32
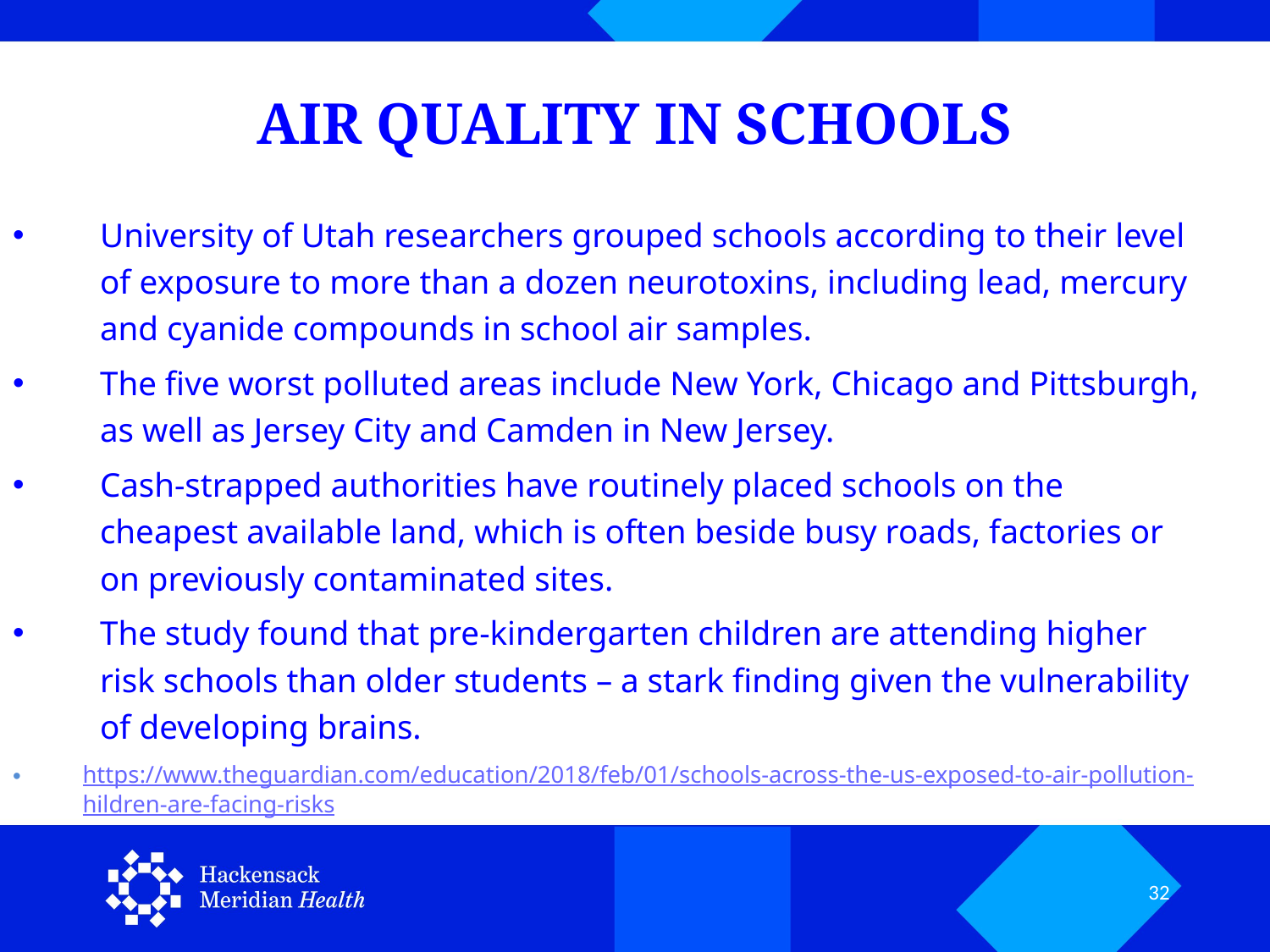

# AIR QUALITY IN SCHOOLS
University of Utah researchers grouped schools according to their level of exposure to more than a dozen neurotoxins, including lead, mercury and cyanide compounds in school air samples.
The five worst polluted areas include New York, Chicago and Pittsburgh, as well as Jersey City and Camden in New Jersey.
Cash-strapped authorities have routinely placed schools on the cheapest available land, which is often beside busy roads, factories or on previously contaminated sites.
The study found that pre-kindergarten children are attending higher risk schools than older students – a stark finding given the vulnerability of developing brains.
https://www.theguardian.com/education/2018/feb/01/schools-across-the-us-exposed-to-air-pollution-hildren-are-facing-risks
32

## Slide 33
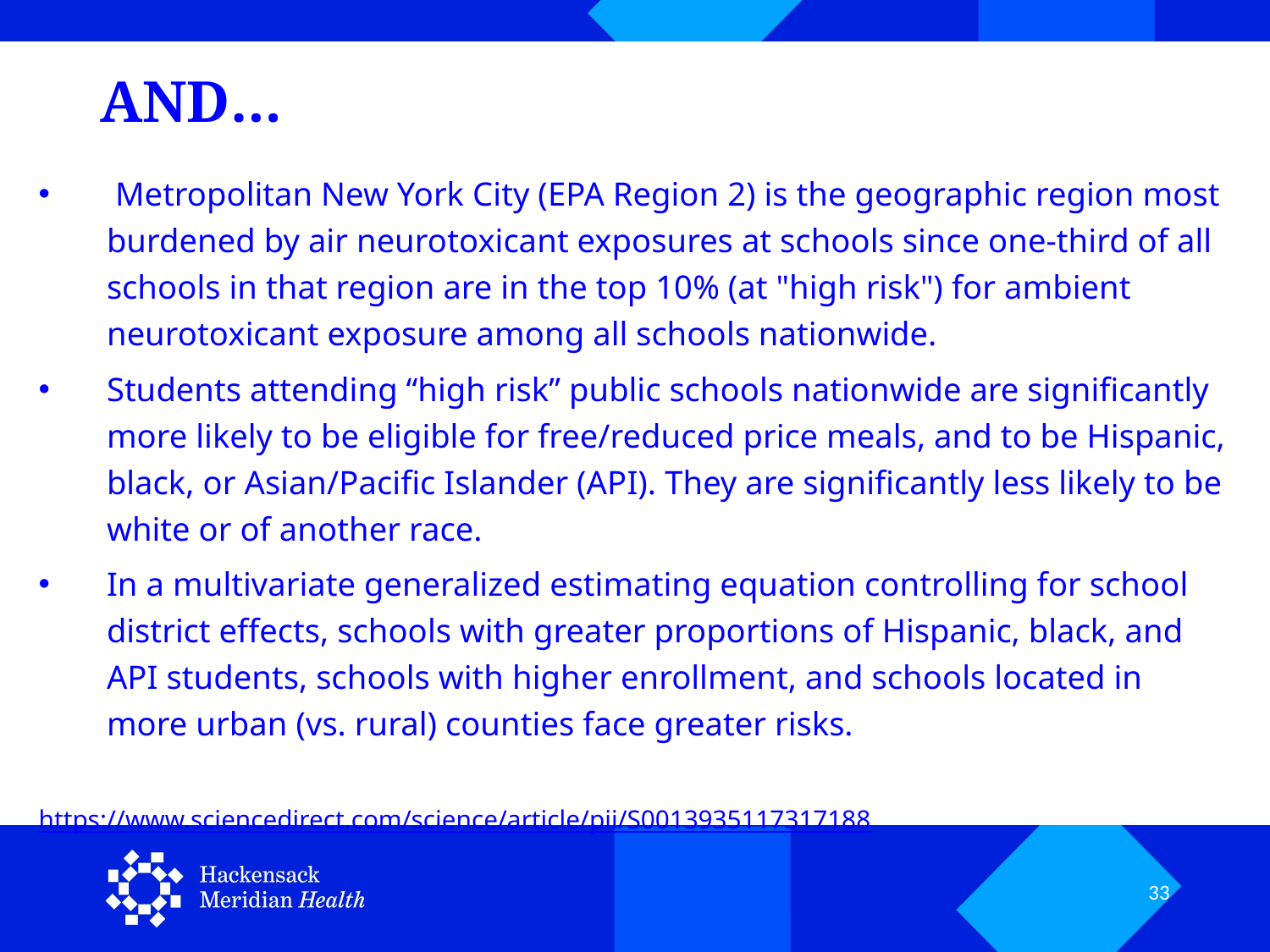

# AND…
 Metropolitan New York City (EPA Region 2) is the geographic region most burdened by air neurotoxicant exposures at schools since one-third of all schools in that region are in the top 10% (at "high risk") for ambient neurotoxicant exposure among all schools nationwide.
Students attending “high risk” public schools nationwide are significantly more likely to be eligible for free/reduced price meals, and to be Hispanic, black, or Asian/Pacific Islander (API). They are significantly less likely to be white or of another race.
In a multivariate generalized estimating equation controlling for school district effects, schools with greater proportions of Hispanic, black, and API students, schools with higher enrollment, and schools located in more urban (vs. rural) counties face greater risks.
https://www.sciencedirect.com/science/article/pii/S0013935117317188
33

## Slide 34
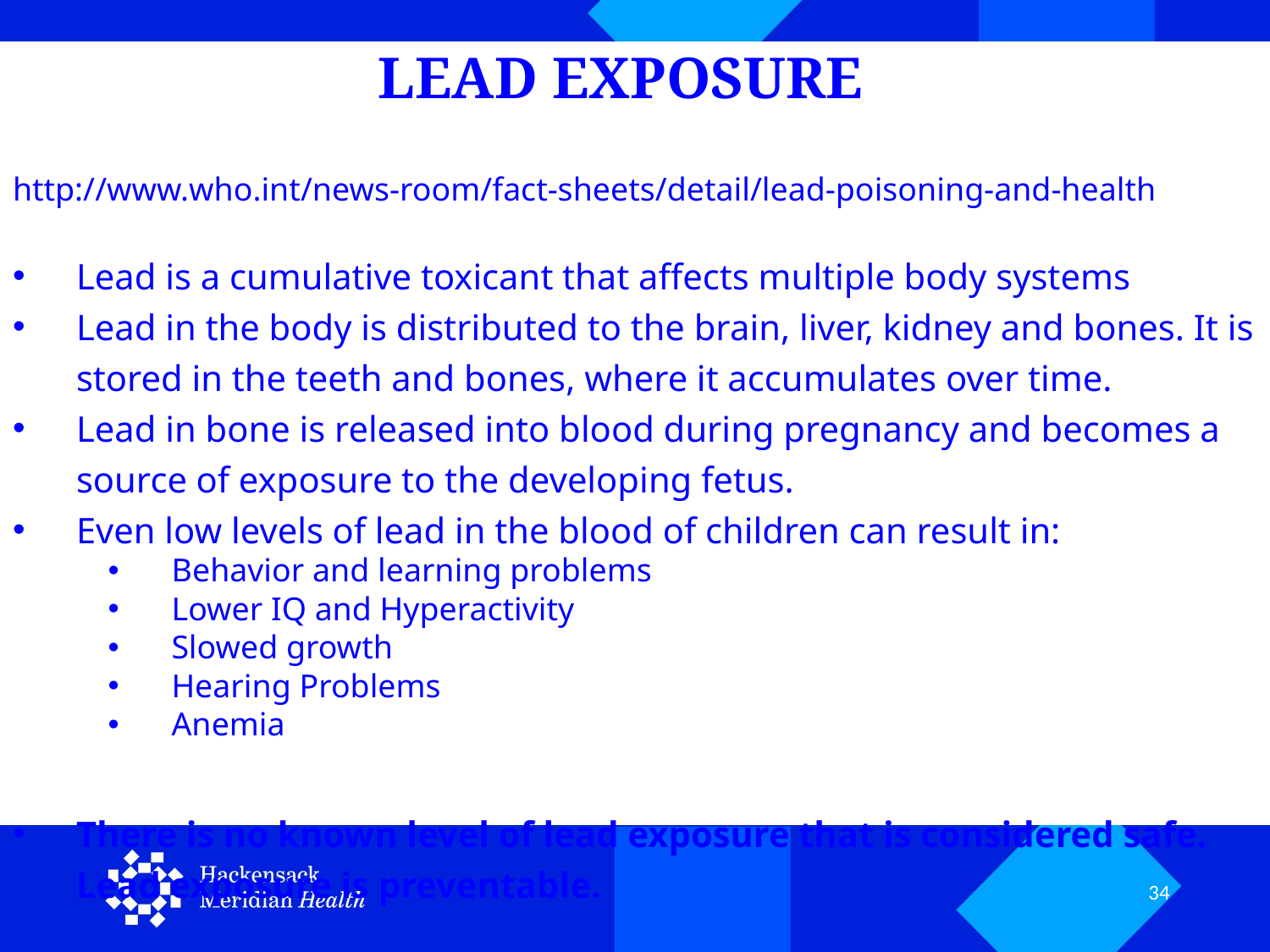

# LEAD EXPOSURE
http://www.who.int/news-room/fact-sheets/detail/lead-poisoning-and-health
Lead is a cumulative toxicant that affects multiple body systems
Lead in the body is distributed to the brain, liver, kidney and bones. It is stored in the teeth and bones, where it accumulates over time.
Lead in bone is released into blood during pregnancy and becomes a source of exposure to the developing fetus.
Even low levels of lead in the blood of children can result in:
Behavior and learning problems
Lower IQ and Hyperactivity
Slowed growth
Hearing Problems
Anemia
There is no known level of lead exposure that is considered safe. Lead exposure is preventable.
34

## Slide 35
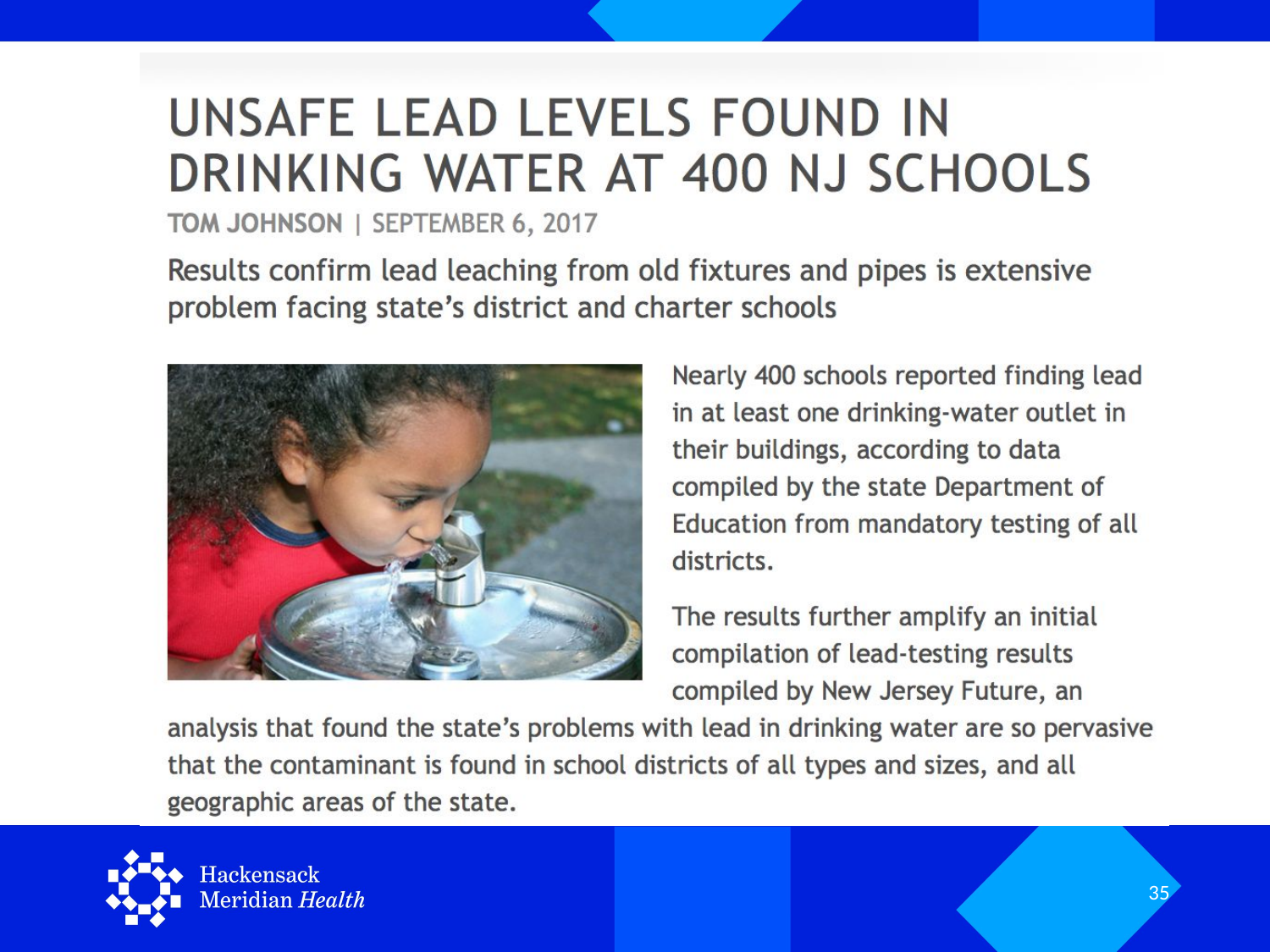

35

## Slide 36
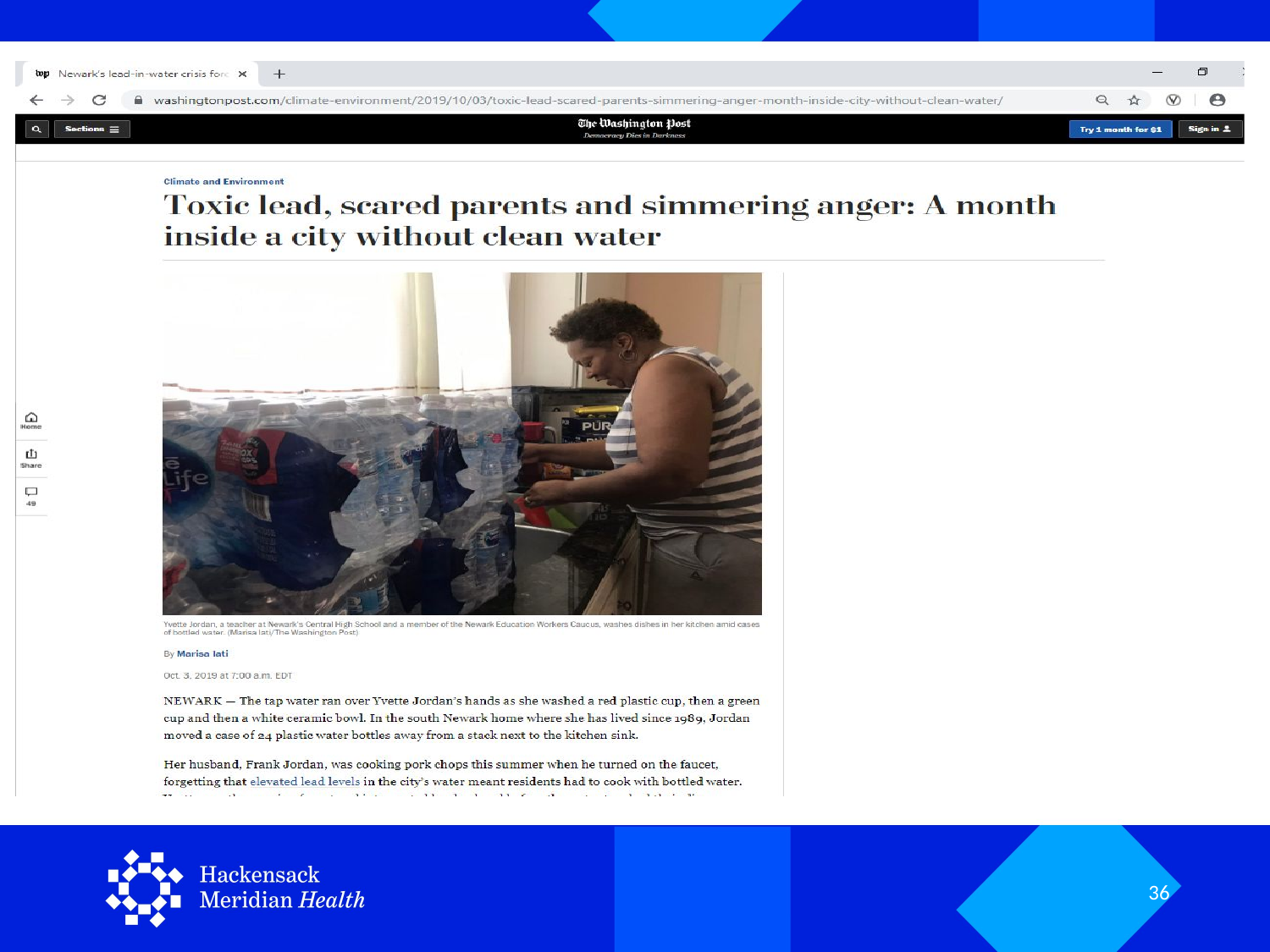

36

## Slide 37
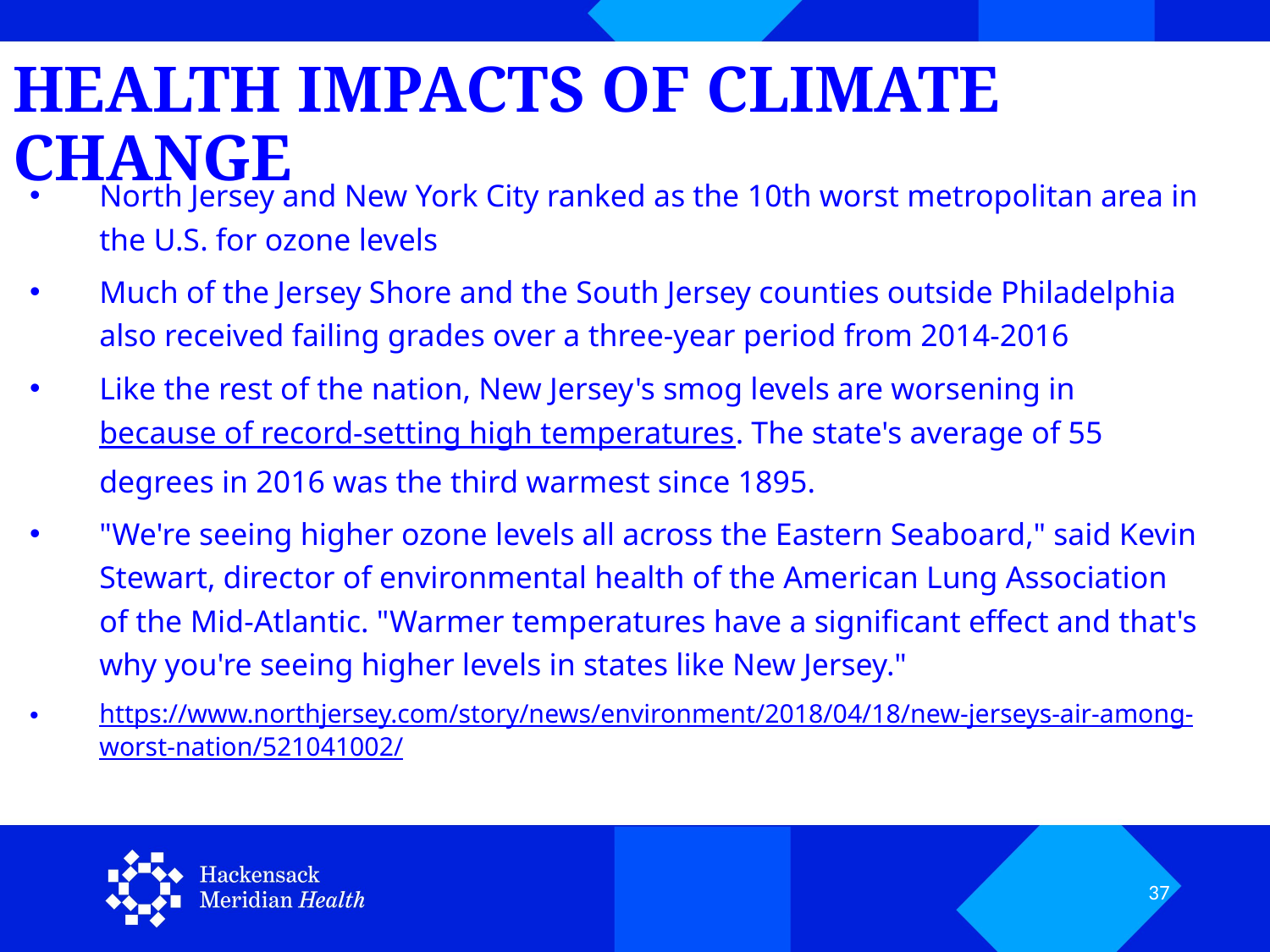

# HEALTH IMPACTS OF CLIMATE CHANGE
North Jersey and New York City ranked as the 10th worst metropolitan area in the U.S. for ozone levels
Much of the Jersey Shore and the South Jersey counties outside Philadelphia also received failing grades over a three-year period from 2014-2016
Like the rest of the nation, New Jersey's smog levels are worsening in  because of record-setting high temperatures. The state's average of 55 degrees in 2016 was the third warmest since 1895.
"We're seeing higher ozone levels all across the Eastern Seaboard," said Kevin Stewart, director of environmental health of the American Lung Association of the Mid-Atlantic. "Warmer temperatures have a significant effect and that's why you're seeing higher levels in states like New Jersey."
https://www.northjersey.com/story/news/environment/2018/04/18/new-jerseys-air-among-worst-nation/521041002/
37

## Slide 38
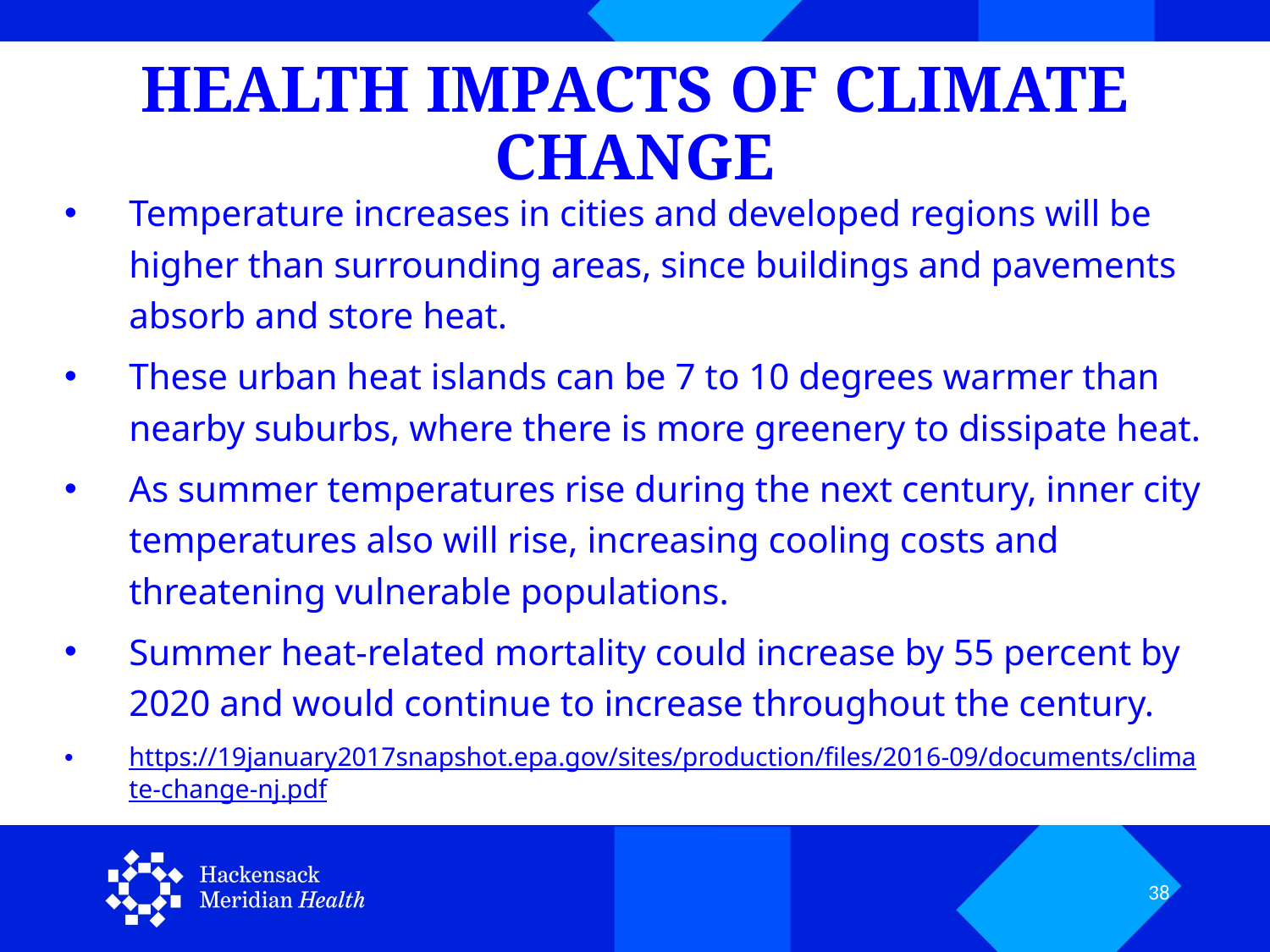

# HEALTH IMPACTS OF CLIMATE CHANGE
Temperature increases in cities and developed regions will be higher than surrounding areas, since buildings and pavements absorb and store heat.
These urban heat islands can be 7 to 10 degrees warmer than nearby suburbs, where there is more greenery to dissipate heat.
As summer temperatures rise during the next century, inner city temperatures also will rise, increasing cooling costs and threatening vulnerable populations.
Summer heat-related mortality could increase by 55 percent by 2020 and would continue to increase throughout the century.
https://19january2017snapshot.epa.gov/sites/production/files/2016-09/documents/climate-change-nj.pdf
38

## Slide 39
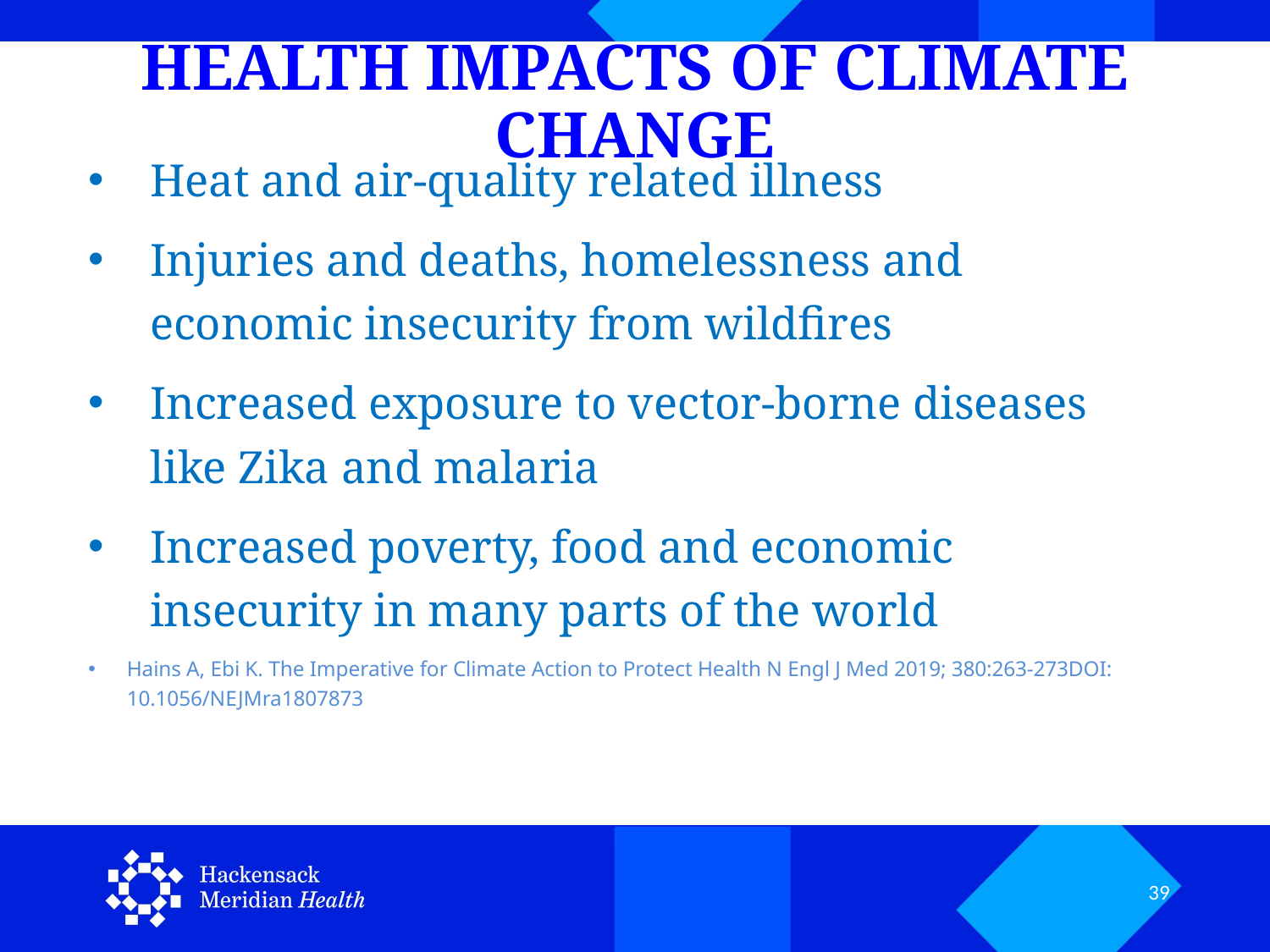

# HEALTH IMPACTS OF CLIMATE CHANGE
Heat and air-quality related illness
Injuries and deaths, homelessness and economic insecurity from wildfires
Increased exposure to vector-borne diseases like Zika and malaria
Increased poverty, food and economic insecurity in many parts of the world
Hains A, Ebi K. The Imperative for Climate Action to Protect Health N Engl J Med 2019; 380:263-273DOI: 10.1056/NEJMra1807873
39

## Slide 40
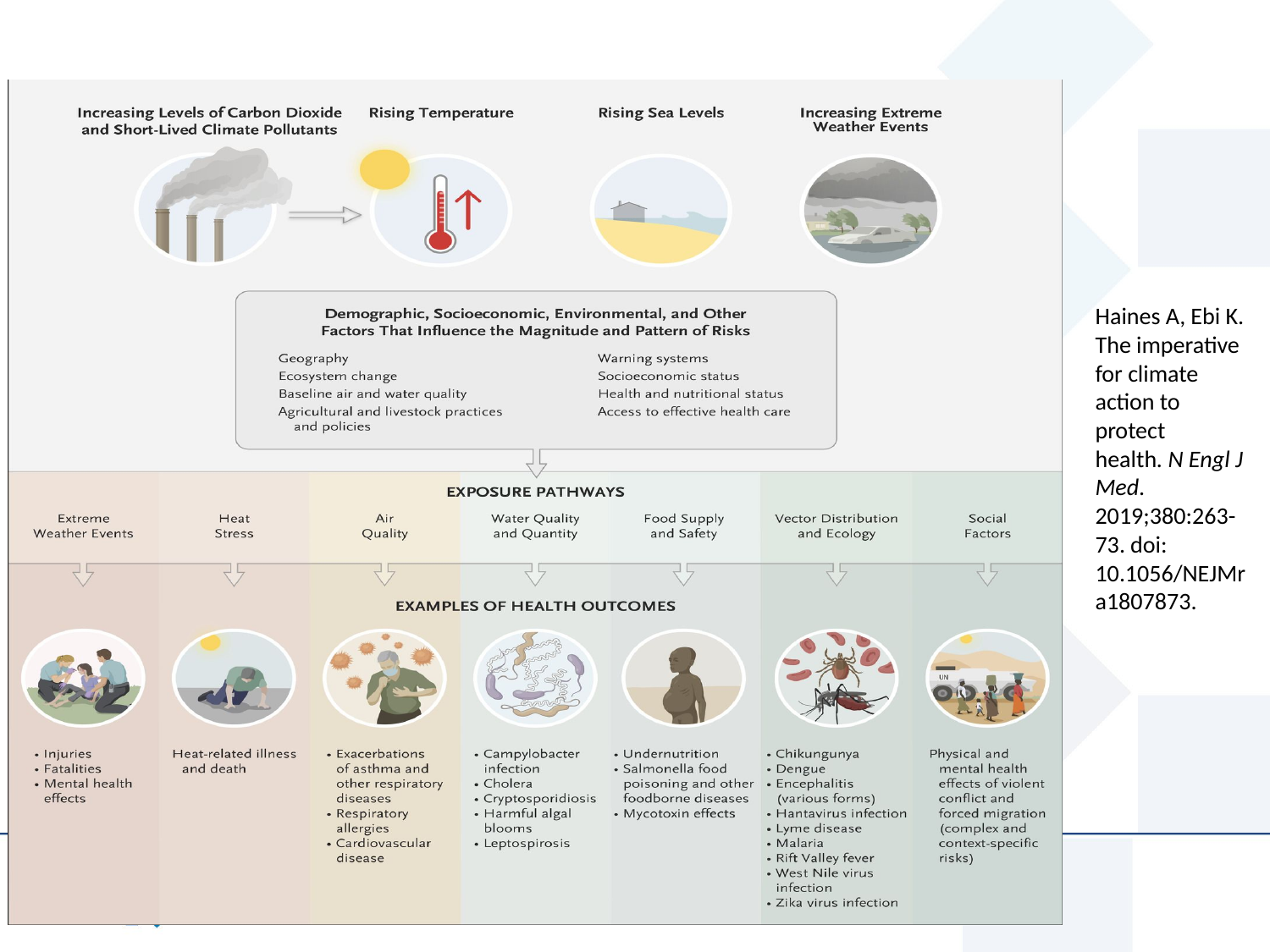

#
Haines A, Ebi K. The imperative for climate action to protect health. N Engl J Med. 2019;380:263-73. doi: 10.1056/NEJMra1807873.

## Slide 41
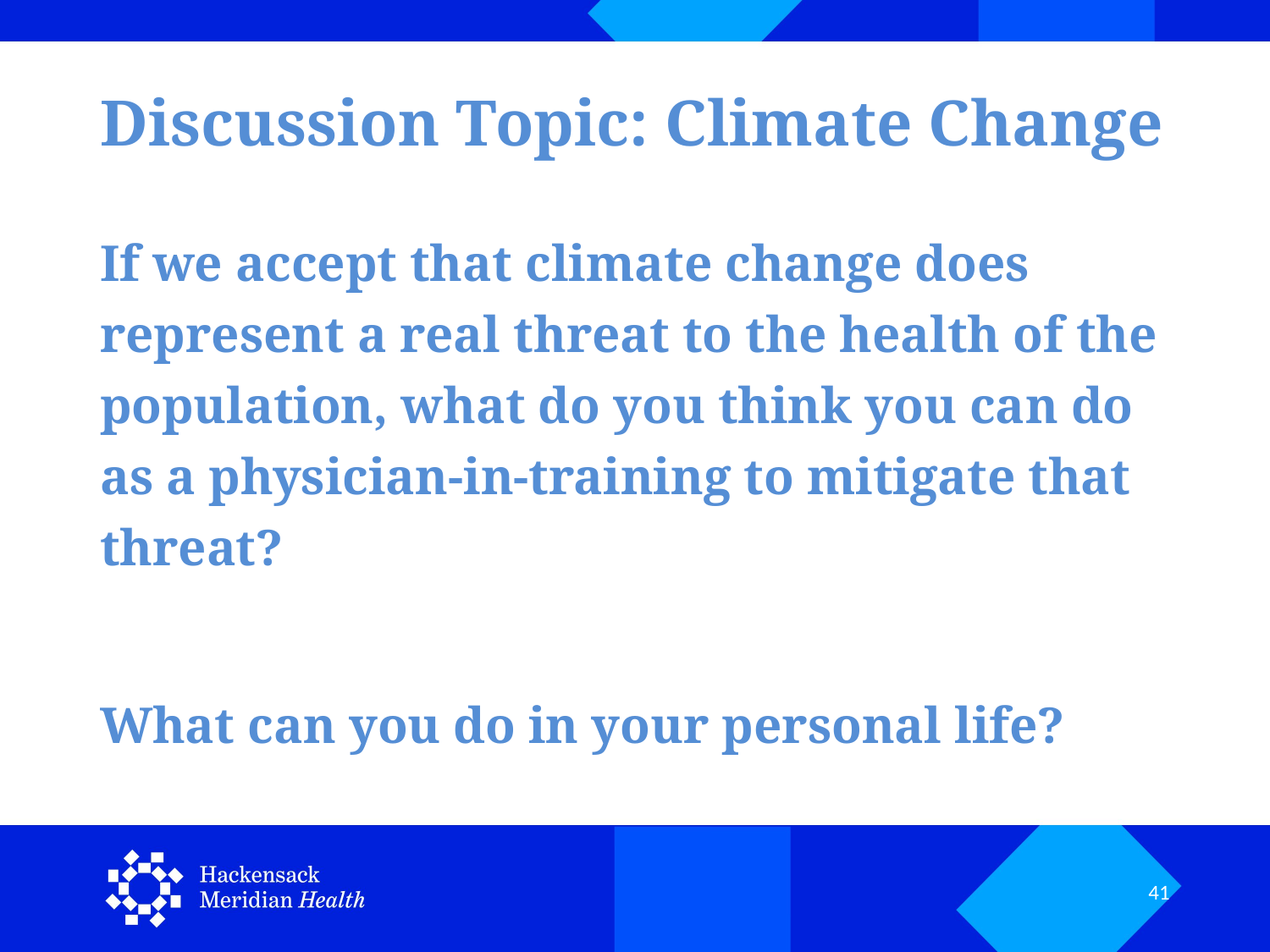

# Discussion Topic: Climate Change
If we accept that climate change does represent a real threat to the health of the population, what do you think you can do as a physician-in-training to mitigate that threat?
What can you do in your personal life?
41

## Slide 42
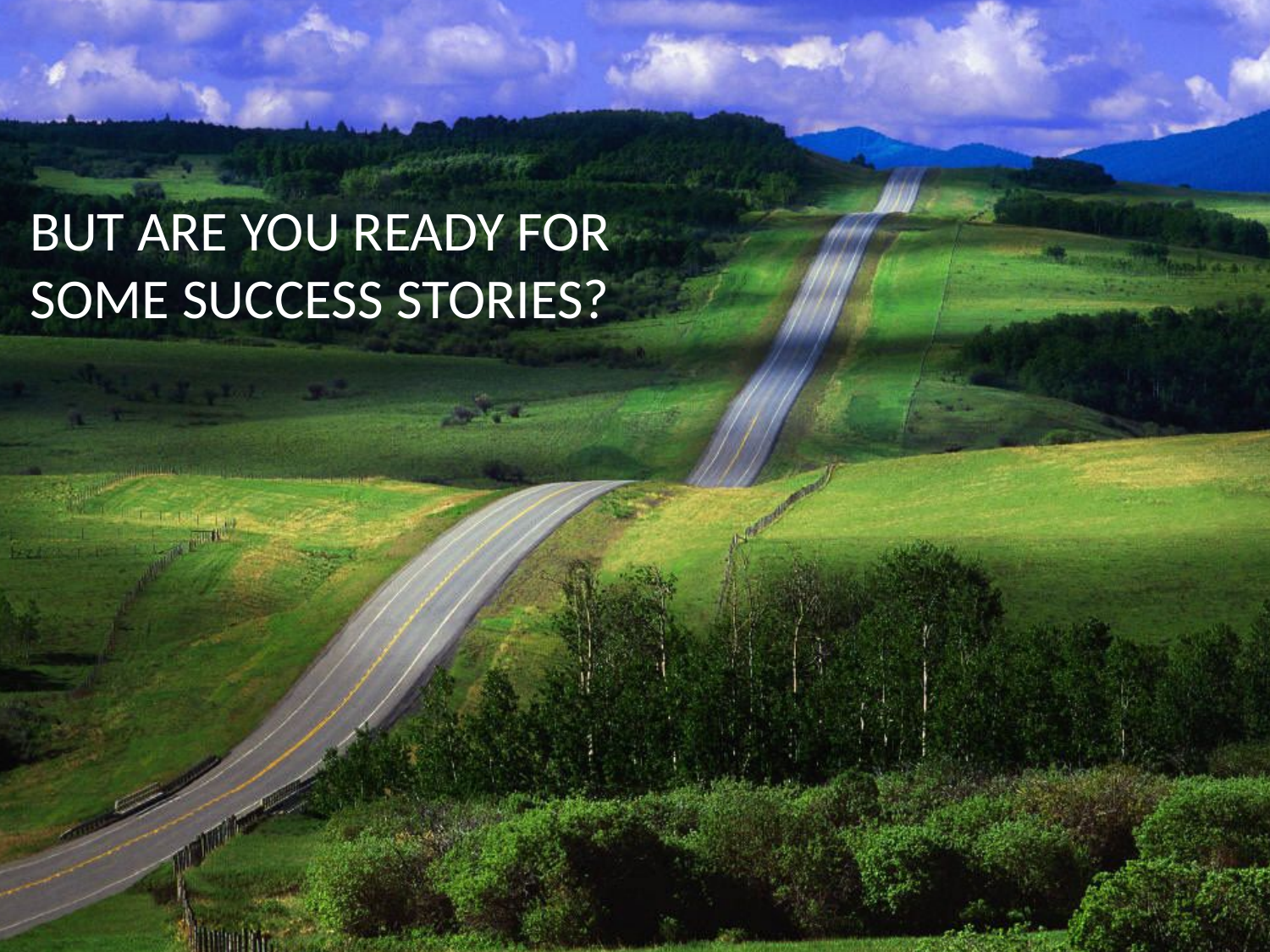

BUT ARE YOU READY FOR
SOME SUCCESS STORIES?
42

## Slide 43
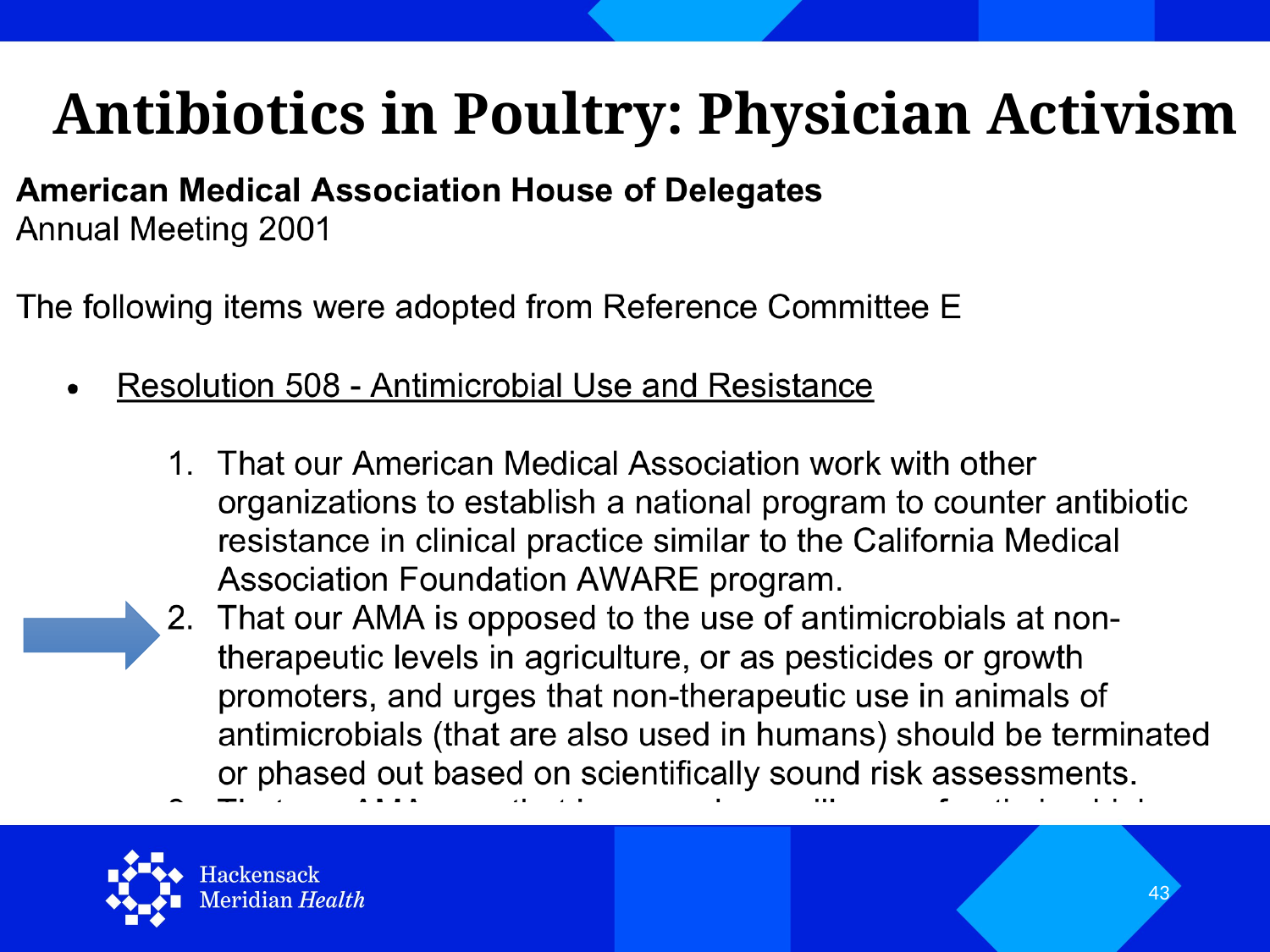

Antibiotics in Poultry: Physician Activism
43

## Slide 44
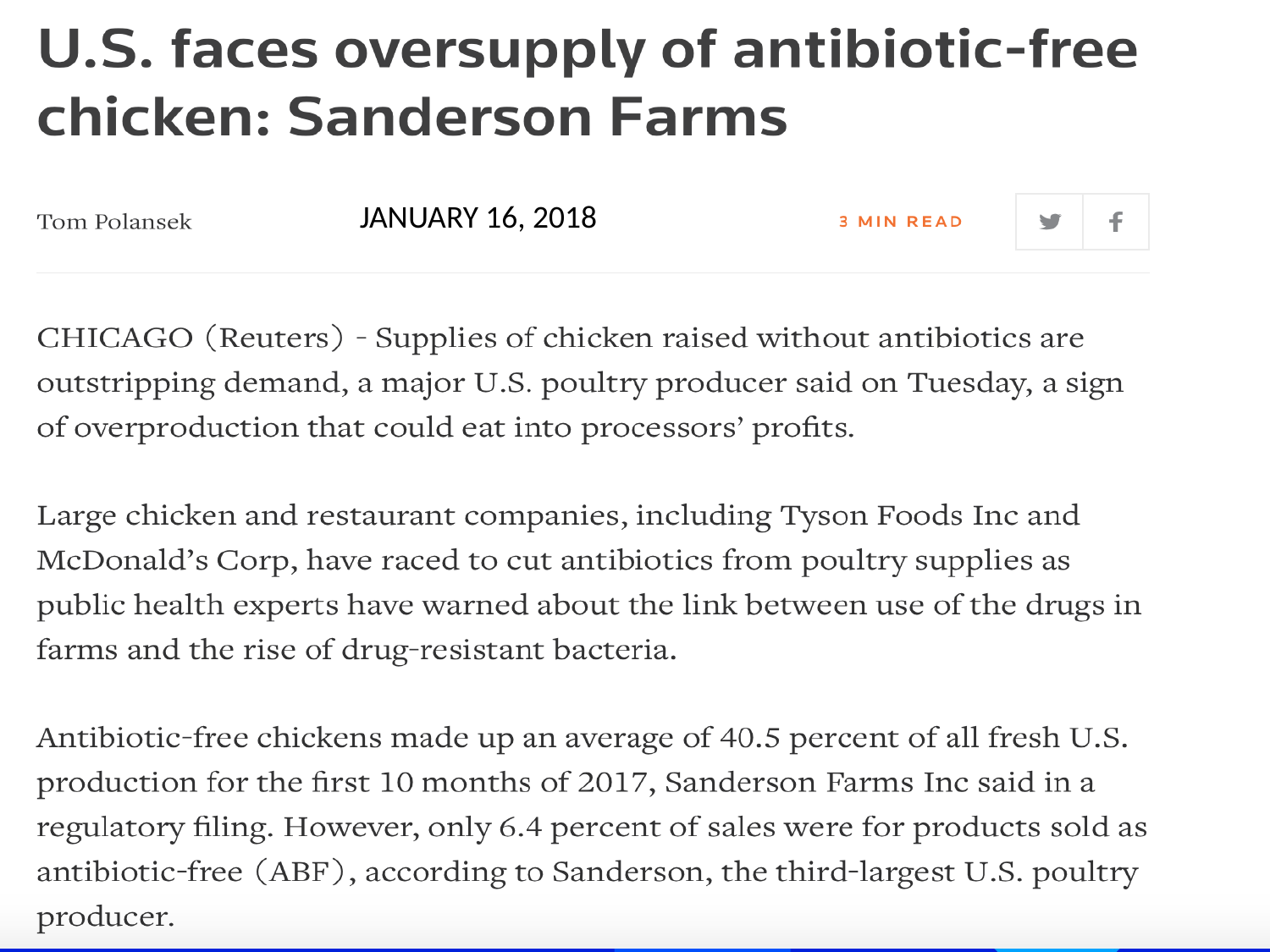

JANUARY 16, 2018
44

## Slide 45
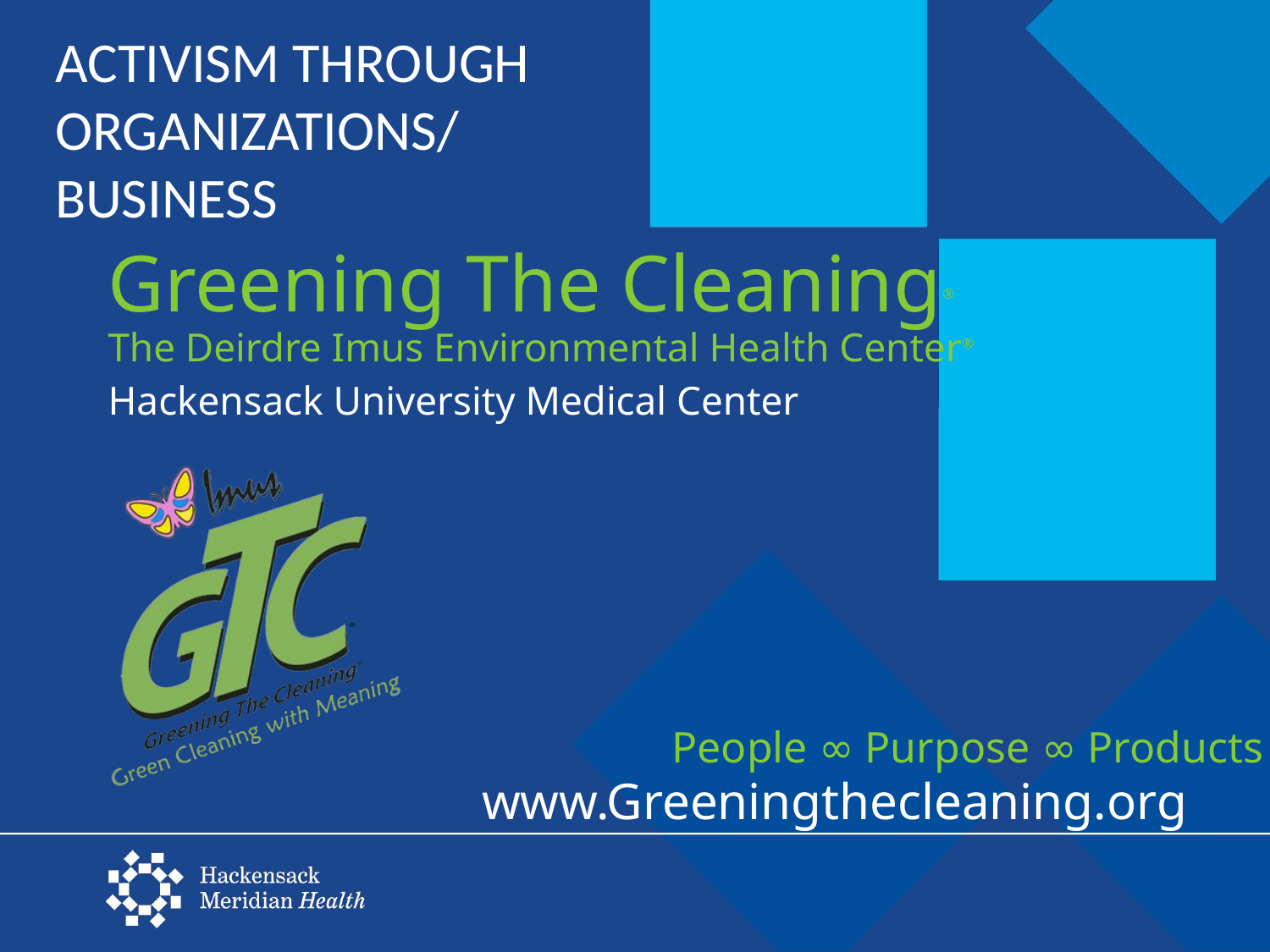

ACTIVISM THROUGH
ORGANIZATIONS/
BUSINESS
# Greening The Cleaning® The Deirdre Imus Environmental Health Center®
Hackensack University Medical Center
People ∞ Purpose ∞ Products
www.Greeningthecleaning.org

## Slide 46
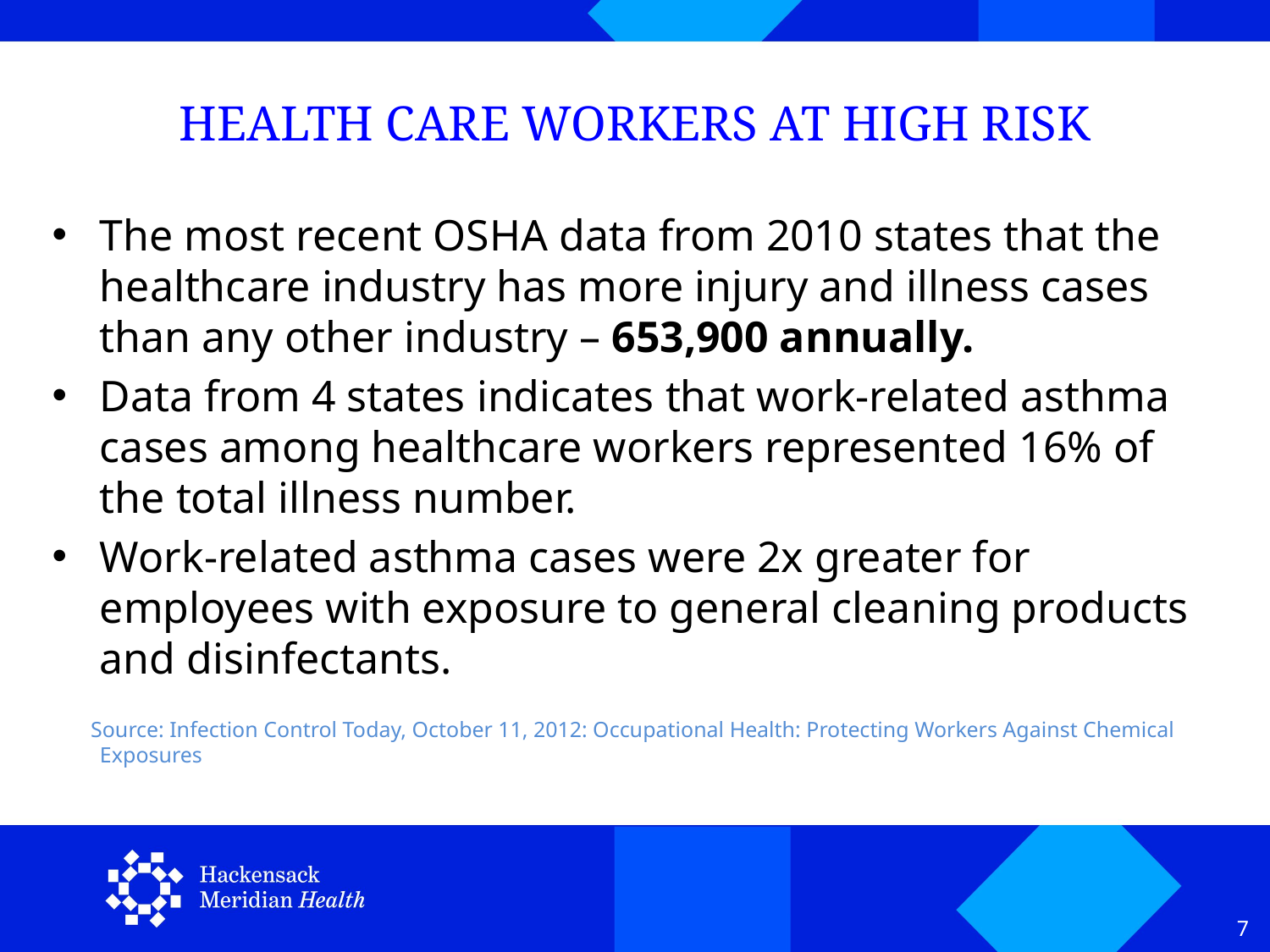

# HEALTH CARE WORKERS AT HIGH RISK
The most recent OSHA data from 2010 states that the healthcare industry has more injury and illness cases than any other industry – 653,900 annually.
Data from 4 states indicates that work-related asthma cases among healthcare workers represented 16% of the total illness number.
Work-related asthma cases were 2x greater for employees with exposure to general cleaning products and disinfectants.
 Source: Infection Control Today, October 11, 2012: Occupational Health: Protecting Workers Against Chemical Exposures
7

## Slide 47
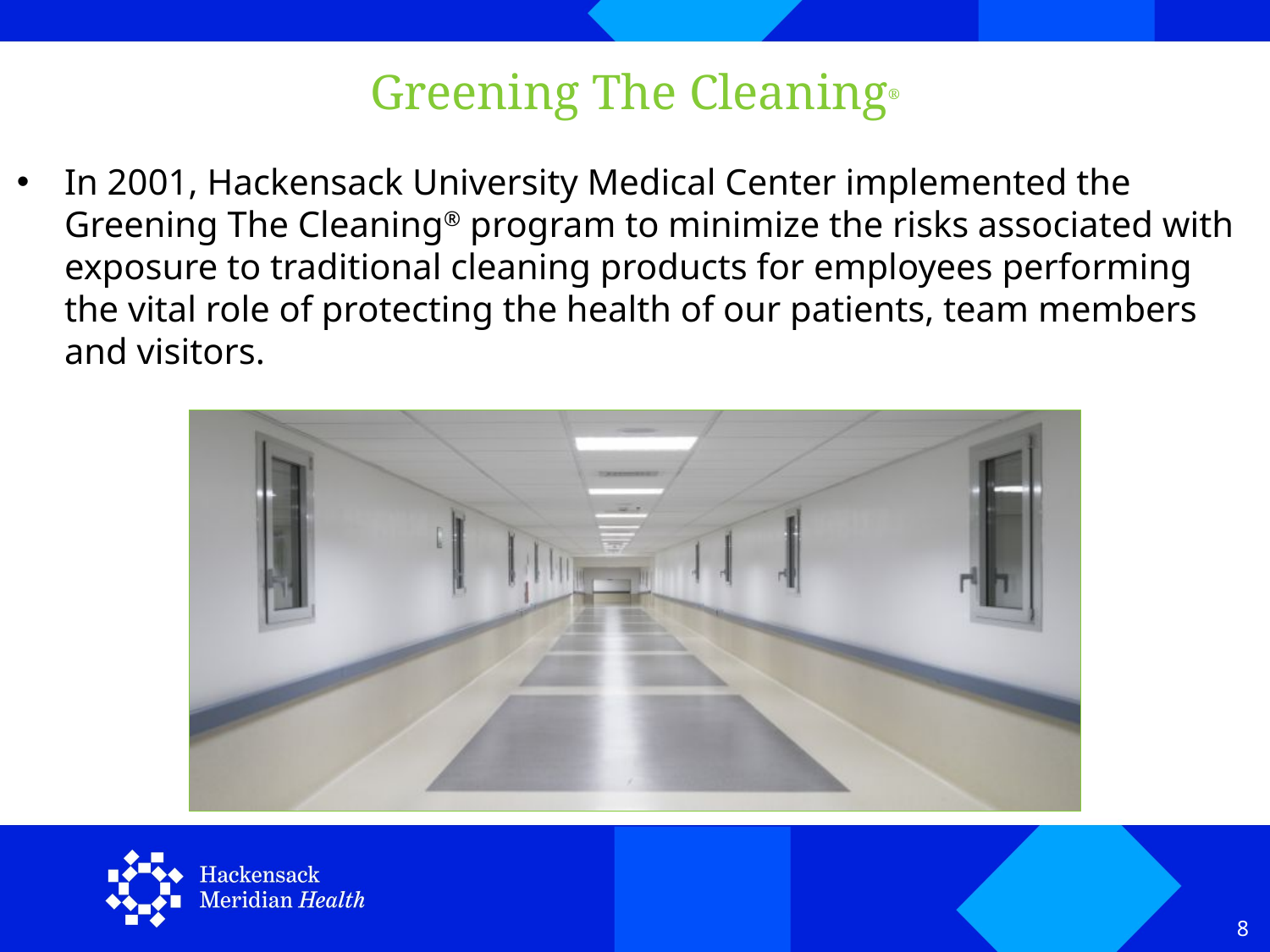

# Greening The Cleaning®
In 2001, Hackensack University Medical Center implemented the Greening The Cleaning® program to minimize the risks associated with exposure to traditional cleaning products for employees performing the vital role of protecting the health of our patients, team members and visitors.
8

## Slide 48
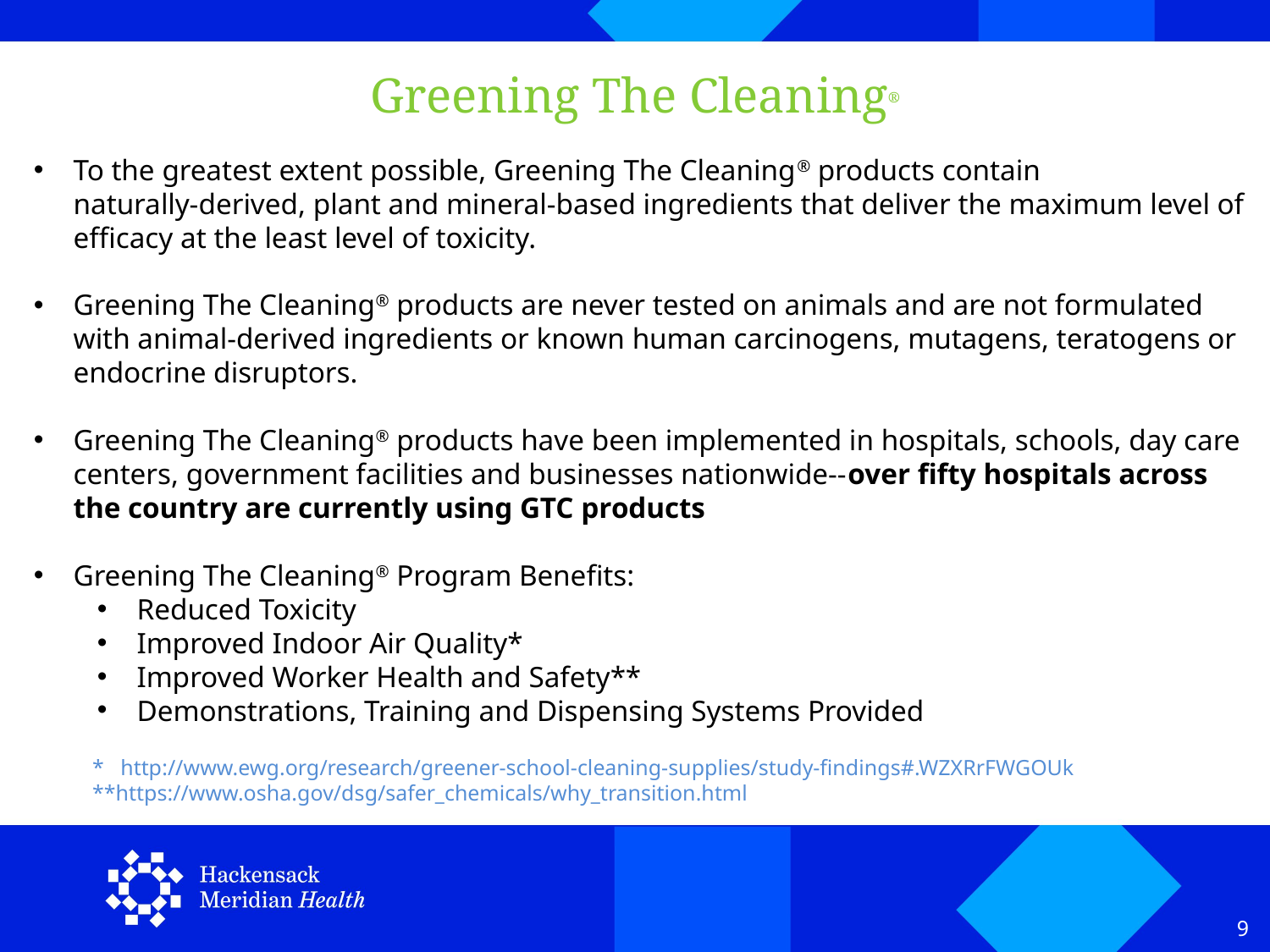

# Greening The Cleaning®
To the greatest extent possible, Greening The Cleaning® products contain
	naturally-derived, plant and mineral-based ingredients that deliver the maximum level of efficacy at the least level of toxicity.
Greening The Cleaning® products are never tested on animals and are not formulated with animal-derived ingredients or known human carcinogens, mutagens, teratogens or endocrine disruptors.
Greening The Cleaning® products have been implemented in hospitals, schools, day care centers, government facilities and businesses nationwide--over fifty hospitals across the country are currently using GTC products
Greening The Cleaning® Program Benefits:
Reduced Toxicity
Improved Indoor Air Quality*
Improved Worker Health and Safety**
Demonstrations, Training and Dispensing Systems Provided
* http://www.ewg.org/research/greener-school-cleaning-supplies/study-findings#.WZXRrFWGOUk
**https://www.osha.gov/dsg/safer_chemicals/why_transition.html
9

## Slide 49
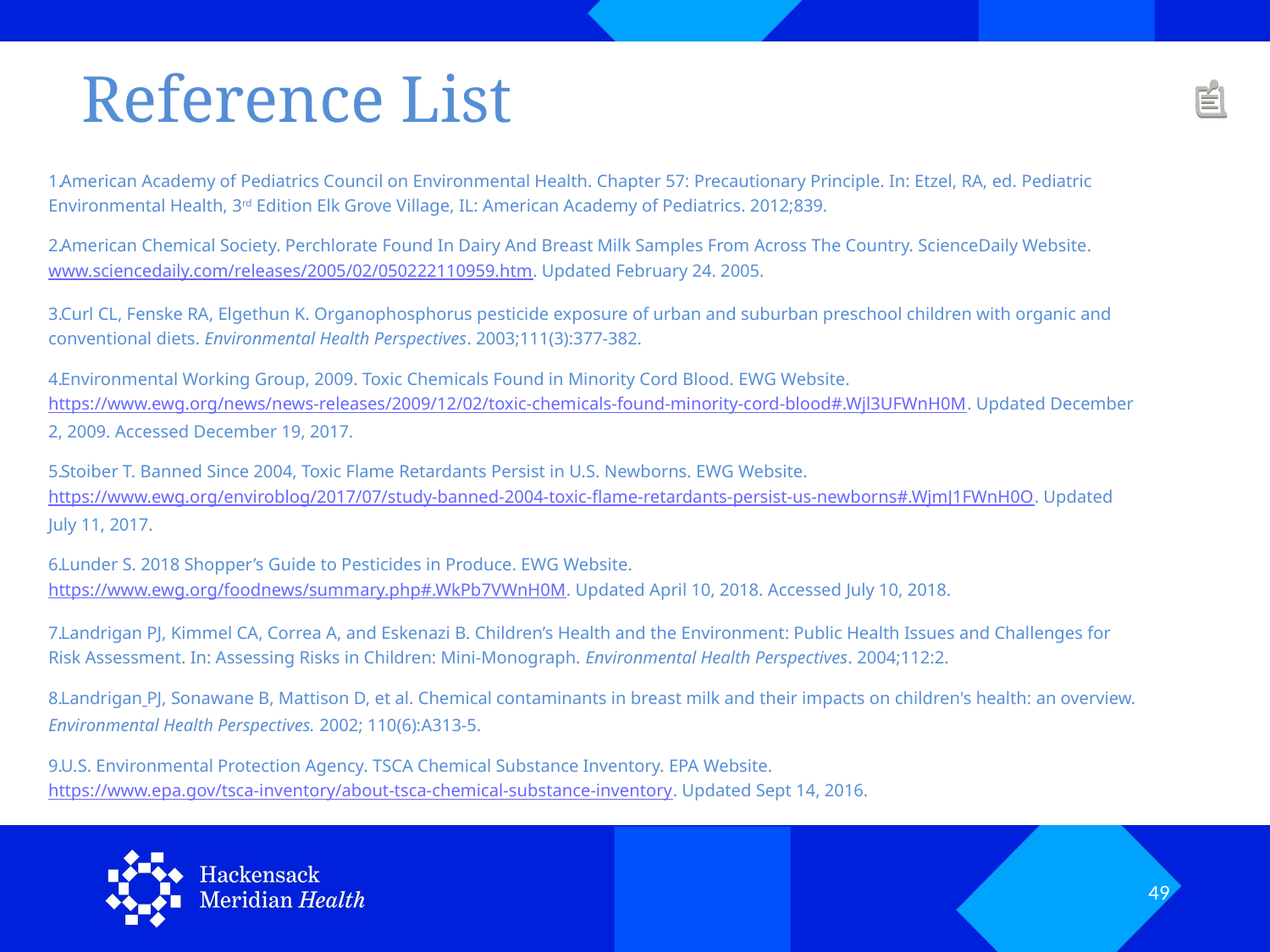

# Reference List
American Academy of Pediatrics Council on Environmental Health. Chapter 57: Precautionary Principle. In: Etzel, RA, ed. Pediatric Environmental Health, 3rd Edition Elk Grove Village, IL: American Academy of Pediatrics. 2012;839.
American Chemical Society. Perchlorate Found In Dairy And Breast Milk Samples From Across The Country. ScienceDaily Website. www.sciencedaily.com/releases/2005/02/050222110959.htm. Updated February 24. 2005.
Curl CL, Fenske RA, Elgethun K. Organophosphorus pesticide exposure of urban and suburban preschool children with organic and conventional diets. Environmental Health Perspectives. 2003;111(3):377-382.
Environmental Working Group, 2009. Toxic Chemicals Found in Minority Cord Blood. EWG Website. https://www.ewg.org/news/news-releases/2009/12/02/toxic-chemicals-found-minority-cord-blood#.Wjl3UFWnH0M. Updated December 2, 2009. Accessed December 19, 2017.
Stoiber T. Banned Since 2004, Toxic Flame Retardants Persist in U.S. Newborns. EWG Website. https://www.ewg.org/enviroblog/2017/07/study-banned-2004-toxic-flame-retardants-persist-us-newborns#.WjmJ1FWnH0O. Updated July 11, 2017.
Lunder S. 2018 Shopper’s Guide to Pesticides in Produce. EWG Website. https://www.ewg.org/foodnews/summary.php#.WkPb7VWnH0M. Updated April 10, 2018. Accessed July 10, 2018.
Landrigan PJ, Kimmel CA, Correa A, and Eskenazi B. Children’s Health and the Environment: Public Health Issues and Challenges for Risk Assessment. In: Assessing Risks in Children: Mini-Monograph. Environmental Health Perspectives. 2004;112:2.
Landrigan PJ, Sonawane B, Mattison D, et al. Chemical contaminants in breast milk and their impacts on children's health: an overview. Environmental Health Perspectives. 2002; 110(6):A313-5.
U.S. Environmental Protection Agency. TSCA Chemical Substance Inventory. EPA Website. https://www.epa.gov/tsca-inventory/about-tsca-chemical-substance-inventory. Updated Sept 14, 2016.
49

## Slide 50
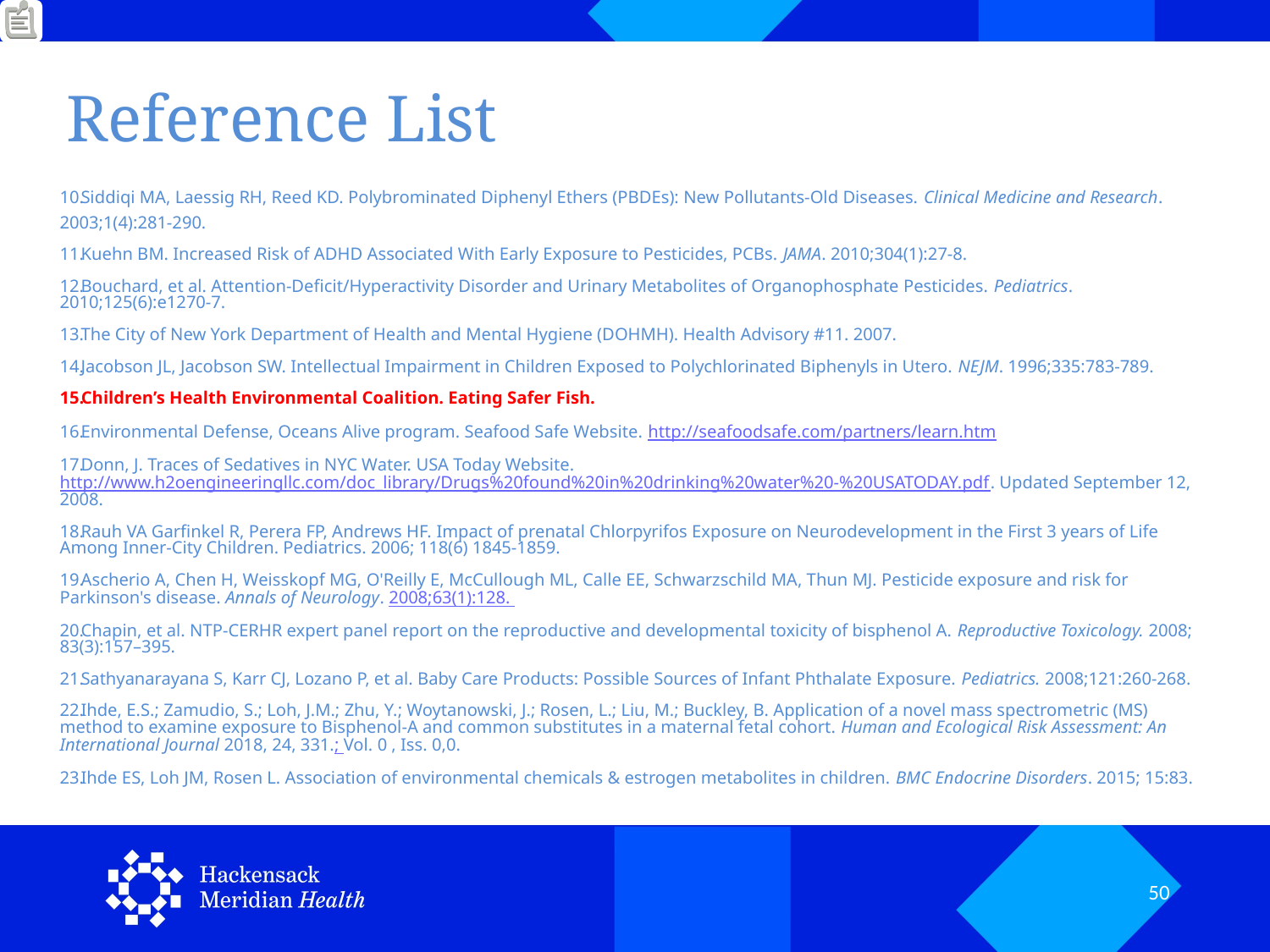

# Reference List
Siddiqi MA, Laessig RH, Reed KD. Polybrominated Diphenyl Ethers (PBDEs): New Pollutants-Old Diseases. Clinical Medicine and Research. 2003;1(4):281-290.
Kuehn BM. Increased Risk of ADHD Associated With Early Exposure to Pesticides, PCBs. JAMA. 2010;304(1):27‐8.
Bouchard, et al. Attention-Deficit/Hyperactivity Disorder and Urinary Metabolites of Organophosphate Pesticides. Pediatrics. 2010;125(6):e1270‐7.
The City of New York Department of Health and Mental Hygiene (DOHMH). Health Advisory #11. 2007.
Jacobson JL, Jacobson SW. Intellectual Impairment in Children Exposed to Polychlorinated Biphenyls in Utero. NEJM. 1996;335:783-789.
Children’s Health Environmental Coalition. Eating Safer Fish.
Environmental Defense, Oceans Alive program. Seafood Safe Website. http://seafoodsafe.com/partners/learn.htm
Donn, J. Traces of Sedatives in NYC Water. USA Today Website. http://www.h2oengineeringllc.com/doc_library/Drugs%20found%20in%20drinking%20water%20-%20USATODAY.pdf. Updated September 12, 2008.
Rauh VA Garfinkel R, Perera FP, Andrews HF. Impact of prenatal Chlorpyrifos Exposure on Neurodevelopment in the First 3 years of Life Among Inner-City Children. Pediatrics. 2006; 118(6) 1845-1859.
Ascherio A, Chen H, Weisskopf MG, O'Reilly E, McCullough ML, Calle EE, Schwarzschild MA, Thun MJ. Pesticide exposure and risk for Parkinson's disease. Annals of Neurology. 2008;63(1):128.
Chapin, et al. NTP-CERHR expert panel report on the reproductive and developmental toxicity of bisphenol A. Reproductive Toxicology. 2008; 83(3):157–395.
Sathyanarayana S, Karr CJ, Lozano P, et al. Baby Care Products: Possible Sources of Infant Phthalate Exposure. Pediatrics. 2008;121:260-268.
Ihde, E.S.; Zamudio, S.; Loh, J.M.; Zhu, Y.; Woytanowski, J.; Rosen, L.; Liu, M.; Buckley, B. Application of a novel mass spectrometric (MS) method to examine exposure to Bisphenol-A and common substitutes in a maternal fetal cohort. Human and Ecological Risk Assessment: An International Journal 2018, 24, 331.; Vol. 0 , Iss. 0,0.
Ihde ES, Loh JM, Rosen L. Association of environmental chemicals & estrogen metabolites in children. BMC Endocrine Disorders. 2015; 15:83.
50

## Slide 51
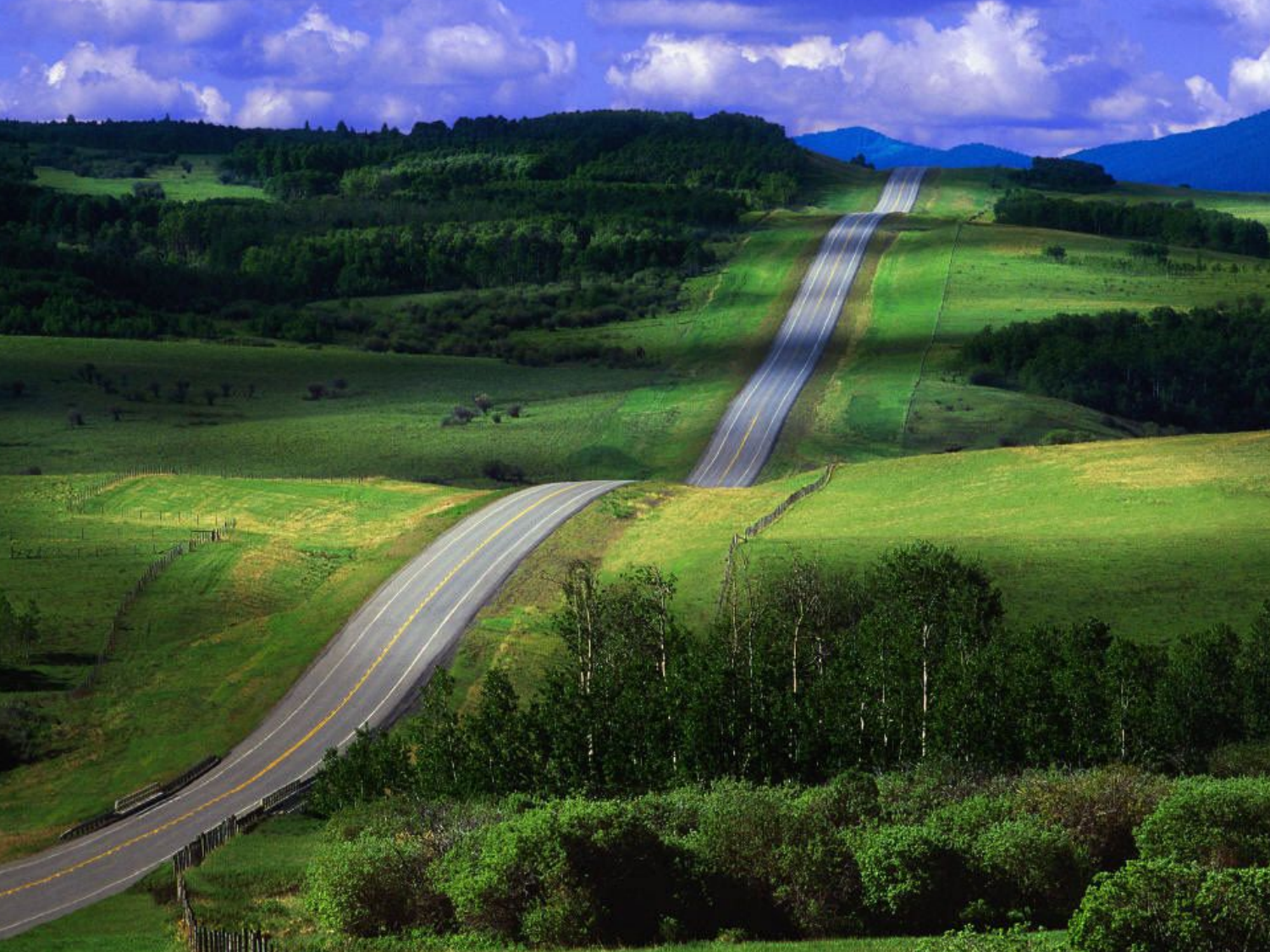

51
