# Supplementary material for: The impact of integrating environmental health into medical school curricula: a survey-based study
Source: BMC Med Educ. 2021 Jan 8;21:40. doi: 10.1186/s12909-020-02458-x (PMC7796639; doi:10.1186/s12909-020-02458-x)
Supplement: Supplementary file 2 — Additional file 2. [file 12909_2020_2458_MOESM2_ESM.docx]

# **Environmental Health Assignment**

**Using the EWG Healthy Living app:**

1. Rate at least five personal care or household products that you use.
2. Rate at least ten food or drink items that you consume regularly.
3. Select 1-2 things you found surprising in each category and one thing in each category you would consider changing based on what you learned.

**Environmental Health Assignment Debrief (15 mins)**

Using the EWG Healthy Living app, rate:

1. At least five personal care or household products that you use

2. At least ten food or drink items that you consume regularly

Make a note of 1-2 things you found surprising in each category and one thing in each category you would consider changing based on what you learned. Be ready to discuss these products in the small group session on 12/11.

Faculty discussion prompts

- Ask students to share insights gained through this assignment. What were your previous thoughts about these products and what are they now?
- Thinking about the stages of change, how ready are you to change this behavior? Has anyone changed behaviors based on these findings? Why or why not?
- Using a scale from 1-10 (with 1 being not confident at all and 10 being completely confident), how confident are you that you can make this change?
  - Then faculty can follow up their score with “Why a *6* and not a *4* (faculty chooses a lesser #)?” This technique helps people move across the spectrum of change (towards action) which faculty can use as a teachable tool, and example.
- How do you approach others about change?
- Discuss barriers and challenges. Consider economic barriers and behavioral barriers.
- Emphasize small, incremental changes, with measurable timely and achievable goals. Emphasize education and awareness as first step.
